# Water-Soluble Chiral Cyclic Peptoids and Their Sodium and Gadolinium Complexes: Study of Conformational and Relaxometric Properties

**DOI:** 10.1021/acs.joc.2c02713

**Published:** 2023-05-08

**Authors:** Assunta D’Amato, Linhai Jiang, Giorgio Della Sala, Kent Kirshenbaum, Chiara Costabile, Chiara Furlan, Eliana Gianolio, Irene Izzo, Francesco De Riccardis

**Affiliations:** † Department of Chemistry and Biology “A. Zambelli”, University of Salerno, Via Giovanni Paolo II, 132, Fisciano, SA 84084, Italy; ‡ Department of Chemistry, New York University, 100 Washington Square East, New York, New York 10003-6688, United States; § Department of Molecular Biotechnology and Health Sciences and Molecular Imaging Center, University of Turin, Via Nizza, 52, 10126 Turin, Italy

## Abstract

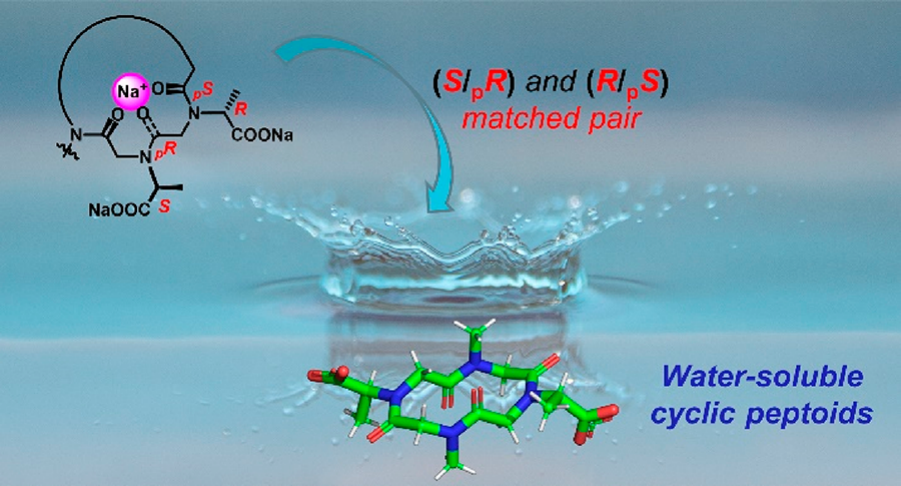

Cyclic peptoids are macrocyclic oligomers of N-substituted glycines with specific folding abilities and excellent metal binding properties. In this work, we show how strategic positioning of chiral (*S*)- and (*R*)-(1-carboxyethyl)glycine units influences the conformational stability of water-soluble macrocyclic peptoids as sodium complexes. The reported results are based on nuclear magnetic resonance spectroscopy, extensive computational studies, and X-ray diffraction analysis using single crystals grown from aqueous solutions. The studies include ^1^H relaxometric investigations of hexameric cyclic peptoids in the presence of the Gd^3+^ ion to assess their thermodynamic stabilities and relaxivities.

## Introduction

Peptoid macrocycles (N-substituted glycine cyclooligomers) make up an emerging family of peptidomimetics that hold substantial promise for application in biomedicine^1,2^ due to their straightforward synthesis,^3,4^ vast chemical diversity, and unique structural attributes.^5−7^

Efforts to control their conformation have been plagued by the weak intramolecular hydrogen bond network^8^ and fast *cis*/*trans* tertiary amide interconversion.^9^ However, covalent constraints induced by head-to-tail cyclization,^10^ metal chelation,^11−13^ and stereoelectronic effects between side chains^14^ and the backbone^15^ play a significant role in rigidifying their structure.^6,16,17^ Interestingly, in contrast with linear congeners,^18−24^ few contributions report structural studies dealing with water-soluble cyclic peptoids.^25^ To fill this gap, we embarked on the synthesis of the first chiral, water-soluble, cyclic tetra-, hexa-, and octamer peptoids incorporating *N*-(*S*)- and *N*-(*R*)-(1-carboxyethyl)glycine monomers (*N*sce and *N*rce, respectively), also mixed with sarcosine, and explored their general conformational properties as sodium salts. We also investigated the complexation and relaxivity properties of cyclohexameric peptoids with the paramagnetic Gd^3+^ ion as a continuation of our work toward the identification of new magnetic resonance imaging probes.^26^

## Results and Discussion

### Design Principles

**Figure 1:**
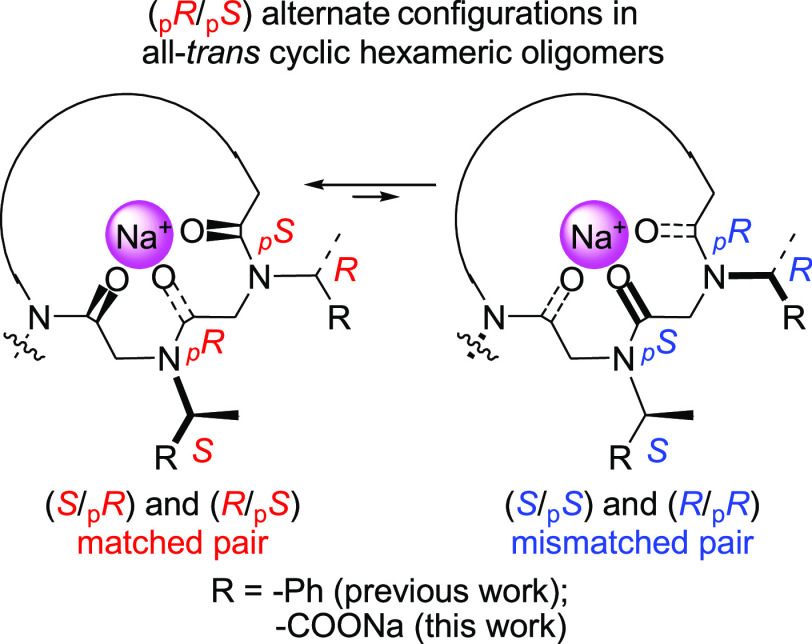
Schematic structures of equilibrating conformational isomers of chiral metalated cyclic peptoids showing matched (*S*/_p_*R*)_*trans*_ (*R*/_p_*S*)_*trans*_ (left) and mismatched (*S*/_p_*S*)_*trans*_ (*R*/_p_*R*)_*trans*_ configurational pairs (right).

Previous studies by our group demonstrated the remarkable templating effect of metals on cyclic peptoids.^11,13,27^ Further stereochemical investigations revealed^17^ that side chains with the *S* configuration induce _p_*R* planar chirality on proximal *trans* amide junctions.^5^ We therefore assumed that, in cyclohexameric all-*trans* peptoid metal complexes, chiral (*S*)- and (*R*)-*N-*(1-carboxyethyl) side chains would evoke _p_*R* and _p_*S* amide bond configurations, respectively [rather than (*S*/_p_*S*)_*trans*_ or (*R*/_p_*R*)_*trans*_ mismatched pairs (Figure 1)].

Weaker metal/carbonyl dipolar interactions in cyclic octameric peptoids (showing a larger inner cavity) made the (*S*/_p_*R*)_*trans*_/(*R*/_p_*S*)_*trans*_ configurational matching less stringent.^11^

**Figure 2:**
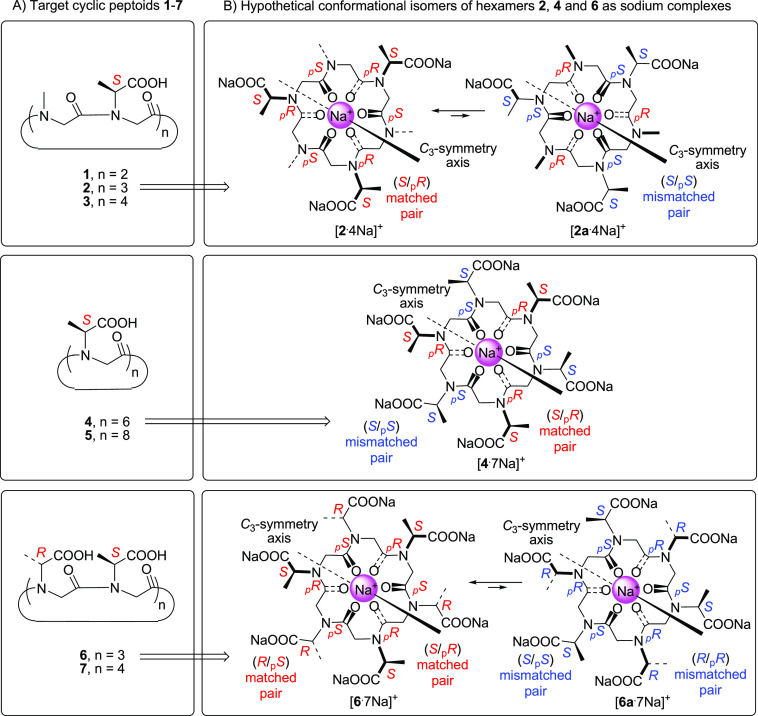
(A) Schematic structures of the three families of macrocycles synthesized in this paper: **1**–**3**, alternated (*S*)-*N*-(1-carboxyethyl)glycine/sarcosine cyclooligomers; **4** and **5**, (*S*)-*N*-(1-carboxyethyl)glycine cyclohomo-oligomers; **6** and **7**, alternated (*S*)/(*R*)-*N*-(1-carboxyethyl)glycine cyclooligomers. (B) Schematic structures of the hypothetical most stable conformations for hexameric cyclopeptoids **2**, **4**, and **6** as sodium complexes with configurational notations. The formal counteranion of the sodium complexes is the phosphate ion of the buffer.

To corroborate those assumptions and reveal the first structures of the metal complexes of the cyclic peptoids in water solution, we designed three families of polycarboxylated macrocycles. The first group displayed alternating units of chiral *N-*(*S*)-(1-carboxyethyl)glycine (*N*sce) and sarcosine [**1**–**3** (Figure 2A, top)]. For the cyclohexameric models, we envisaged the formation of a conformational isomer with paired (*S*/_p_*R*)_*trans*_ configurations (i.e., [**2**·4Na]^+^), energetically favored with respect to the mismatched (*S*/_p_*S*)_*trans*_ [**2a**·4Na]^+^ conformer (Figure 2B, top).

A second group of compounds included homo-oligomeric sequences incorporating *N-*(*S*)-(1-carboxyethyl)glycine units [in **4** and **5** (Figure 2A, middle)], which tested the effects of the mismatched (*S*/_*p*_*S*)*_trans_* pairs in the hexameric all-*trans* [**4**·7Na]^+^ (Figure 2B, middle).

Finally, the third group of cyclooligomers contained alternating (*S*)- and (*R*)-*N*-(1-carboxyethyl)glycine units [i.e., **6** and **7** (Figure 2A, bottom)], designed to form fully matched (*S*/_*p*_*R*)*_trans_*/(*R*/_*p*_*S*)*_trans_* hexameric cyclopeptoids [**6**·7Na]^+^ (Figure 2B, bottom), which would be favored with respect to the fully mismatched conformer [**6a**·7Na]^+^.

Cyclization reactions with a variety of oligomers containing four residues successfully generated only the alternating cyclic tetrapeptoid **1**. This result is consistent with our previous finding that it is extremely difficult to achieve the cyclization of tetrameric peptoids bearing all four α-branched side chains.^17^

Finally, our designs included the formation of Gd^3+^ ion complexes with **2**, **4**, and **6** hexamer peptoids as we planned ^1^H relaxometric investigations to assess their thermodynamic stabilities and relaxivities. This aspect of our study is a continuation of our search for new magnetic resonance imaging (MRI) agents based on cyclic oligoamides.^26^

### Synthesis of Macrocycles **1–7** and Conformational Properties of Their Sodium Salts

**Figure 3:**
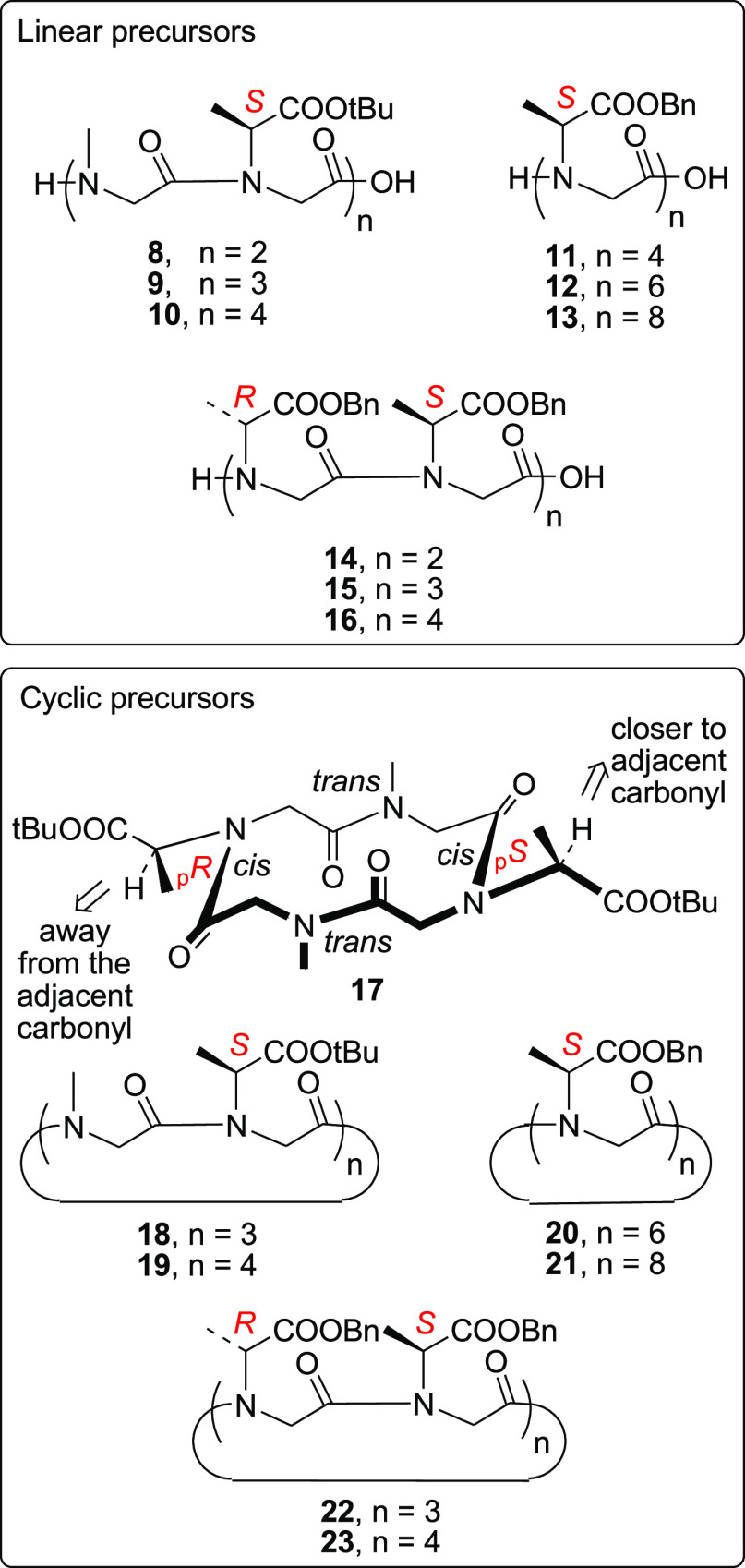
Linear (**8**–**16**) and cyclic precursors (**17**–**23**) of target cyclic peptoids **1**–**7**.

Linear peptoids **8**–**10** (Figure 3), precursors of macrocycles **1**–**3**, respectively, were synthesized on 2-chlorotrityl resin using the mixed peptoid “submonomer” protocol^4^ (see the Experimental Section) incorporating l-alanine, as *tert*-butyl ester (as the chiral unit), and methylamine (as submonomers). Linear oligomers **12**, **13**, **15**, and **16** (precursors of cyclopeptoids **4**–**7**, respectively) were generated on the same resin using l- and d-alanine, as benzyl esters. The use of the less bulky benzyl ester (instead of the *tert*-butyl ester) facilitated the growth of sequences containing contiguous α-branched side chains.

Head-to-tail cyclization of oligomers **8**–**16** was performed with HATU as the condensing agent under high-dilution conditions (0.003 M). Macrocycles **17**–**23** were isolated in reasonable purity [>70%, HPLC analysis (Supporting Information)] and variable yields (17–70%) through reverse-phase chromatography (**19**–**21** and **23**) or precipitation from hot acetonitrile solutions (**17**, **18**, and **22**).

*cyclo*-[*N*sce_(Bn)4_] and *cyclo*-[(*N*rce_(Bn)_-*N*sce_(Bn)_)_2_] could not be formed from the corresponding linear precursors H-*N*sce_(Bn)4_-OH (**11**) and H-(*N*rce_(Bn)_-*N*sce_(Bn)_)_2_-OH (**14**), respectively, even at 50 °C, due to the known^17^ difficulties in cyclizing small Nα-branched cyclooligomers (see the Experimental Section).

**Figure 4:**
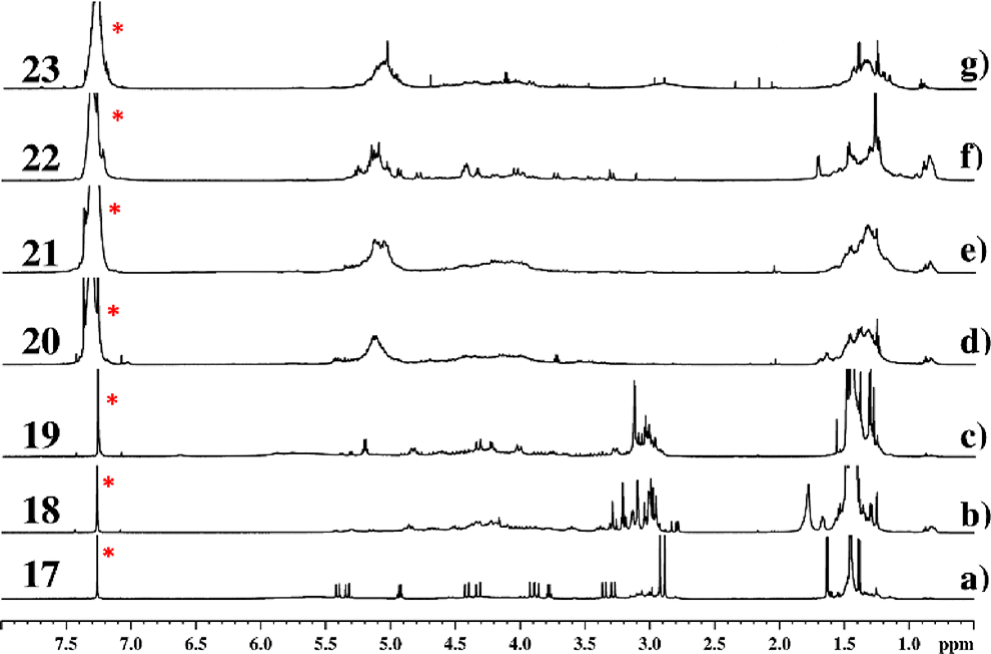
^1^H NMR spectra of C-protected cyclic peptoids **17**–**23**. Residual solvent peaks are labeled with asterisks (600 MHz, CDCl_3_, 0.5–8 ppm expansion).

Of the seven C-protected macrocyclic precursors formed (**17**–**23**), tetramer **17** was the only conformationally stable cyclic oligomer detected [NMR analysis (Figure 4)]. It showed a classic *cis*–*trans*–*cis*–*trans* (*ctct*) conformation with the two chiral side chains on the *cis* amide bonds (the only amide junctions capable of accommodating bulky substituents).^17^ The distinct chemical shifts recorded for the side chain methine protons [δ = 4.92 and 3.78, ^1^H NMR analysis (Figure 4, spectrum a)] implied their location on the nonequivalent _p_*S* and _p_*R cis*-amide bond junctions, respectively (as depicted in Figure 3).

Deprotection of the *tert*-butyl and benzyl groups was accomplished under typical acidic conditions (trifluoroacetic acid in dichloromethane, from 60% to 76% yield) or via reductive (hydrogen and palladium on carbon, from 61% to 78% yield) conditions, respectively, and gave the expected carboxylated macrocycles **1**–**7** in good purity [>90%, HPLC analysis (Supporting Information)].

**Figure 5:**
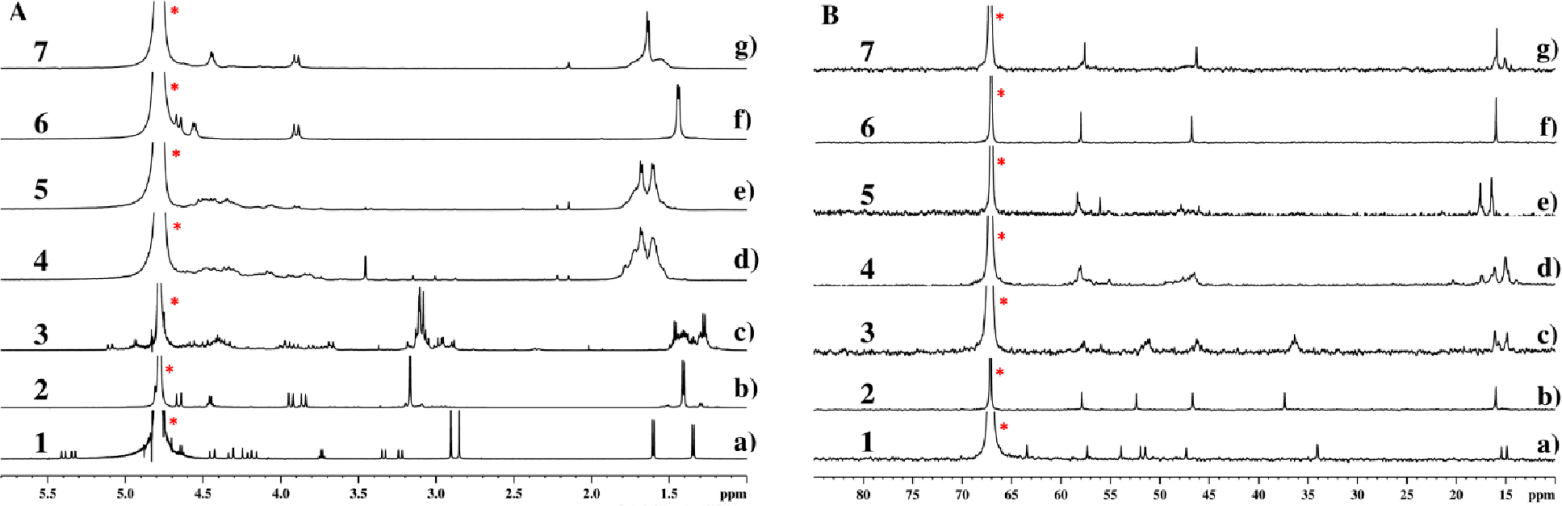
Side-by-side ^1^H and ^13^C NMR spectra of cyclic peptoids **1**–**7**. (A) In ^1^H NMR spectra, residual solvent peaks (HDO) are labeled with asterisks [5.0–10.0 mM solutions in D_2_O with a 0.1 M NaH_2_PO_4_/Na_2_HPO_4_ buffer at pH 8.0 (600 MHz)]. (B) In ^13^C NMR spectra, internal standard (1,4-dioxane) peaks are labeled with a red asterisk [5.0–10.0 mM solutions in D_2_O with a 0.1 M NaH_2_PO_4_/Na_2_HPO_4_ buffer at pH 8.0 (150 MHz)].

The synthesized cyclic peptoids were characterized as sodium salts in D_2_O in a 0.1 M NaH_2_PO_4_/Na_2_HPO_4_ buffer at pH 8.0.^28^ Their conformational properties were deduced from the ^1^H and ^13^C NMR spectra recorded at room temperature and are reported in Figure 5.

### Structural and Theoretical Studies of Alternating (*S*)-*N*-(1-Carboxyethyl)glycine/sarcosine Cyclooligomers **1–3** as Sodium Salts/Complexes

Figure 5 reveals the conformational features of cyclic peptoids **1**–**7** upon dissolution in phosphate buffer (^1^H and ^13^C NMR). [*cyclo*-[(*cis*,_p_*R*)*N*sce-(*trans*,_p_*R*)Sar-(*cis*,_p_*S*)*N*sce-(*trans*,_p_*R*)Sar]·2Na], [**1**·2Na], with its rigid *C*_1_-symmetric arrangement and the nonequivalent chiral side chain methine protons on the distinct _p_*S* and _p_*R cis*-amide bonds [δ = 4.64 and 3.73, ^1^H NMR analysis (Figure 5, spectrum a)], was the only oligomer with an unchanged homogeneous conformation with respect to protected precursor **17**.

**Figure 6:**
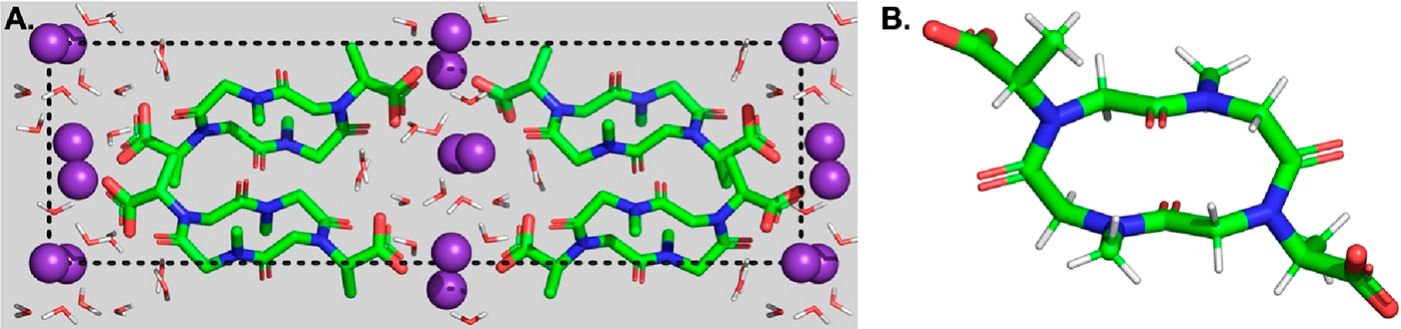
X-ray crystallographic studies of the potassium complex of **1**. (A) Orthoscopic view of the crystal lattice along the *a*-axis. The *b–c* plane of the unit cell is highlighted by black dashed lines. (B) X-ray crystal structure of **1**. Color code: light gray for hydrogen, green for carbon, blue for nitrogen, red for oxygen, and purple for potassium.

Single crystals were obtained for the potassium complex of **1** from the aqueous solution (pH ∼7.5). The ionic interactions between the side chain carboxylate groups and potassium cations were the dominant forces directing the formation of its crystal lattice (Figure 6A). The X-ray crystal structure of this cyclotetrapeptoid confirmed the classic *ctct* amide backbone conformation (Figure 6B), commonly observed for cyclotetrapeptoids in the solid state.^8,25^ Accordingly, we observed the presence of the two (*S*)-*N*-1-carboxyethyl glycine residues at the *cis* amide junctions and the two sarcosine residues at the *trans* peptoid bonds.

Dissolution of cyclic peptoid **2** in a phosphate buffer at pH 8.0 generated a 3-fold symmetric conformationally stable species [NMR analysis (Figure 5, spectrum b, ^1^H NMR)]. Two clearly distinct ^1^H NMR patterns were observed for the side chains. One was related to the CHCH_3_ side chain residues (δ = 4.46, quartet, and 1.42, doublet), and the other to the sarcosines’ methyl groups (δ = 3.18, singlet). Of the two plausible all-*trans* 3-fold symmetric conformations expected for the complex, that containing three pairs of matched (*S*/_p_*R*)^5^ configurations, [**2**·4Na]^+^ (Figure 2B, top), was considered to be preferred.

**Figure 7:**
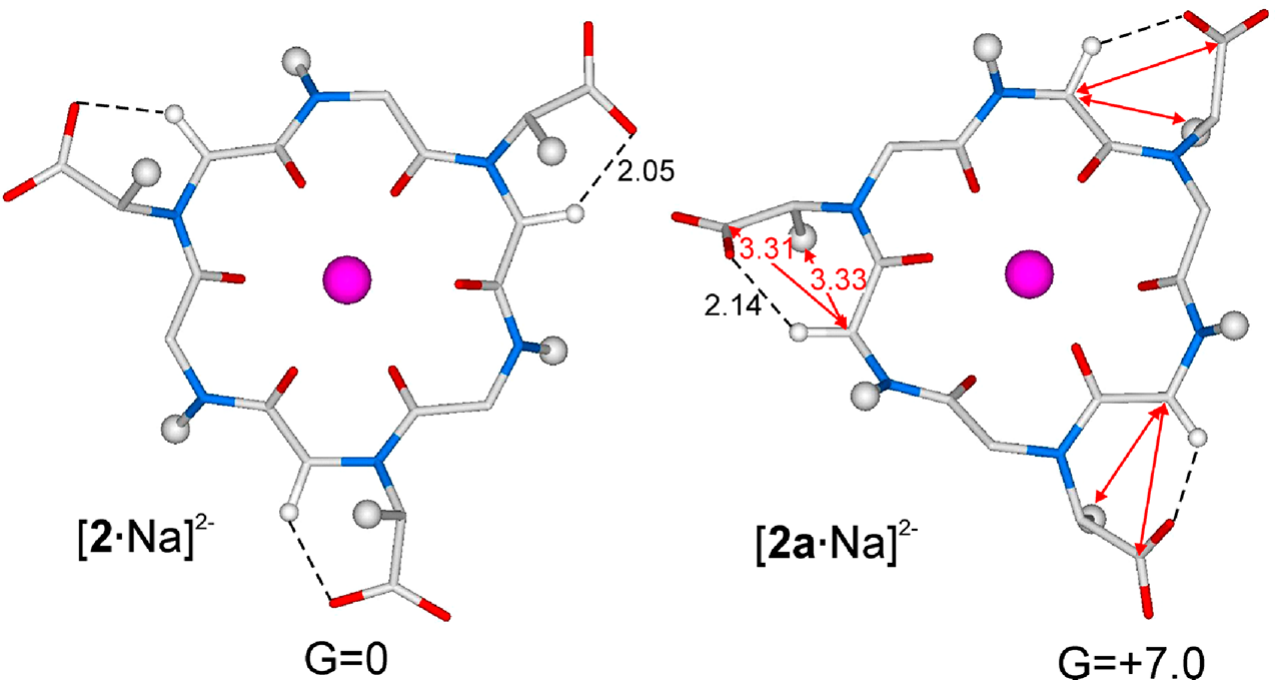
Minimum energy structures of [**2**·Na]^2–^ and [**2a**·Na]^2–^ and their respective free energies calculated in water and expressed in kilocalories per mole. Most hydrogen atoms have been omitted for the sake of clarity. Color code: magenta for Na^+^, light gray for C, light blue for N, red for O, and white for H (see the Supporting Information for the Cartesian coordinates). Distances are given in angstroms. No water molecules have been located close to the Na^+^ ion because its coordination sphere is saturated by the ligand.

To compare the relative energies of conformational isomers [**2**·4Na]^+^ and [**2a**·4Na]^+^, density functional theory (DFT) calculations at the BP86/TZVP level were performed (see the Supporting Information for computational details). Keeping in mind that in a water solution the three Na^+^ counterions of the carboxylate groups would possibly be far from the cyclic peptoids, minimum energy species bearing only one Na^+^ for the possible conformational isomers of cyclohexamer **2** were contemplated. Species [**2**·Na]^2–^ and [**2a**·Na]^2–^, considering the total charge of the calculated structures, are depicted in Figure 7.

According to the results of calculations, the structure of [**2a**·Na]^2–^ was markedly disfavored with respect to [**2**·Na]^2–^ (Δ*G* = 7.0 kcal/mol in a water solution). This finding is mainly related to the repulsive interactions of both the carboxylate carbon and the methyl carbon of the side chain with the methylene of the *n* – 1 residue (Figure 7 and animation in the Supporting Information). Additionally, [**2**·Na]^2–^ shows a favorable hydrogen–carboxylate oxygen attractive interaction [distance of 2.05 Å (Figure 7)].

Although the sodium complexes of cyclic octamer peptoids share similar structural features with the hexameric oligomers, the weaker interactions between intra-annular carbonyl moieties and sodium ions (due to their larger cavity)^11,12^ reduce the stability of their conformation. For this reason, the sodium salt of cyclooligomer **3** appears as a mixture of conformational isomers in slow equilibrium on the NMR time scale (Figure 5, spectra c).

### Structural and Theoretical Studies on (*S*)-*N*-(1-Carboxyethyl)glycine Cyclooligomers **4** and **5** as Sodium Complexes

Dissolution of cyclohexamer **4** in phosphate buffer at pH 8.0 revealed multiple conformations of the sodium complex in slow equilibrium on the NMR time scale [NMR analysis (Figure 5, spectra d)]. The unavoidable mismatched (*S*/_p_*S*) configurational pairs of the hypothetical all-*trans* conformational isomer ([**4**·7Na]^+^, reported in Figure 2B) likely preclude the formation of a stable conformer.

**Figure 8:**
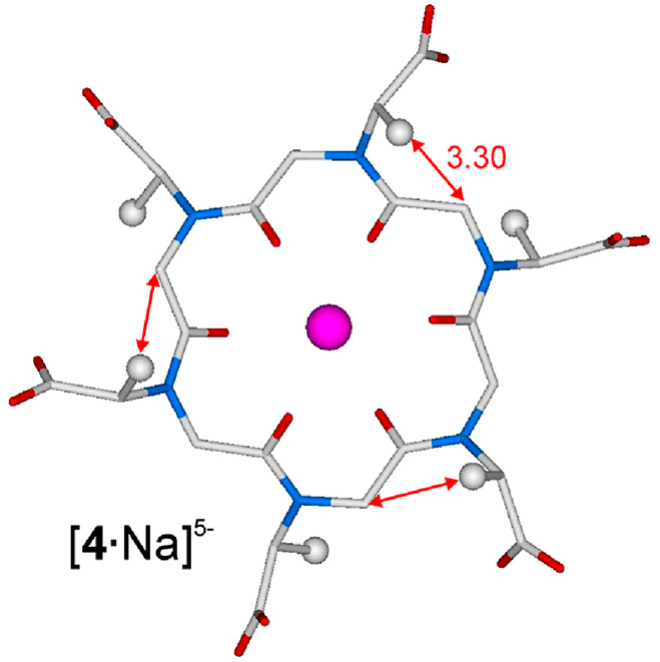
Minimum energy structure of [**4**·Na]^5–^ with reported mismatched interactions (and atomic distances). Hydrogen atoms have been omitted for the sake of clarity. Color code: magenta for Na^+^, light gray for C, light blue for N, and red for O (see the Supporting Information for the Cartesian coordinates). Distances are given in angstroms. No water molecules have been located close to the Na^+^ ion because its coordination sphere is saturated by the ligand.

DFT studies of the monosodiated all-*trans* structure, [**4**·Na]^5–^, evidenced the sterically destabilizing interactions between the methyl groups of the three pairs of (*S*/_p_*S*) side chains and the intra-annular methylenes of the preceding *N*sce units (Figure 8 and animation in the Supporting Information).

The NMR spectra of the corresponding cyclic octamer peptoid [**5**·9Na]^+^ also appeared as a mixture of conformers in slow equilibrium on the NMR time scale (Figure 5, spectra e).

### Structural and Theoretical Studies of (*S*)-*N*-(1-Carboxyethyl)glycine Cyclooligomers **6** and **7** as Sodium Complexes

Dissolution in a phosphate buffer of hexameric cyclic peptoid **6** revealed the formation of a conformationally stable 3-fold symmetric species (Figure 5, spectra f). Of the two alternative possible all-*trans* conformations hypothesized in Figure 2B, [**6**·7Na]^+^ was considered the most plausible, containing fully matched (*S*/_p_*R*)/(*R*/_p_*S*) configurational pairs.

**Figure 9:**
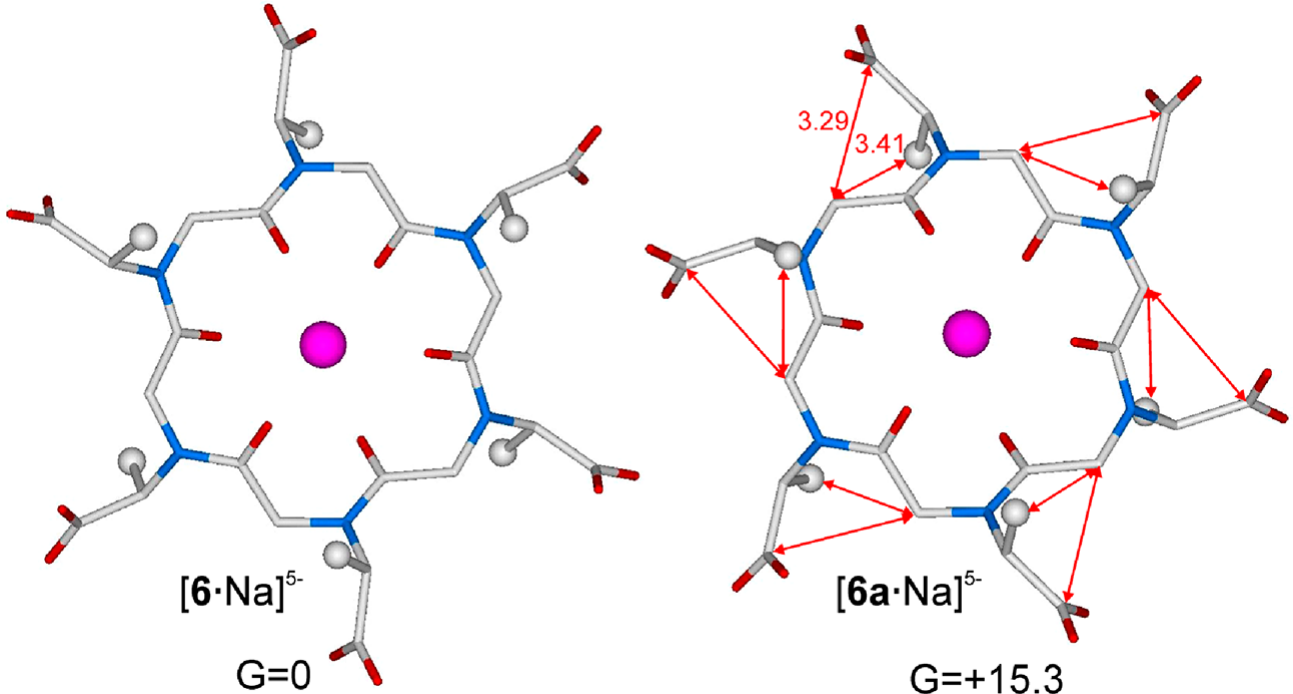
Minimum energy structures of [**6**·Na]^5–^ and [**6a**·Na]^5–^ and their respective free energies calculated in water and expressed in kilocalories per mole. Hydrogen atoms have been omitted for the sake of clarity. Color code: magenta for Na^+^, light gray for C, light blue for N, and red for O (see the Supporting Information for the Cartesian coordinates). Distances are given in angstroms. No water molecules have been located close to the Na^+^ ion because its coordination sphere is saturated by the ligand.

Comparison of the geometries and free energies calculated for the monosodiated [**6**·Na]^5–^ and [**6a**·Na]^5–^ species is reported in Figure 9 (and further described in the animation of the Supporting Information). The significantly higher energy observed for [**6a**·Na]^5–^ (15.3 kcal/mol) was ascribed to repulsive interactions of the carboxylate carbon and the methyl carbon of the side chains with the macrocycle backbone, at all side chain positions (Figure 9).

### Relaxometric Properties of Gd^3+^ Complexes of **2**, **4**, and **6** and Theoretical Studies

**Figure 10:**
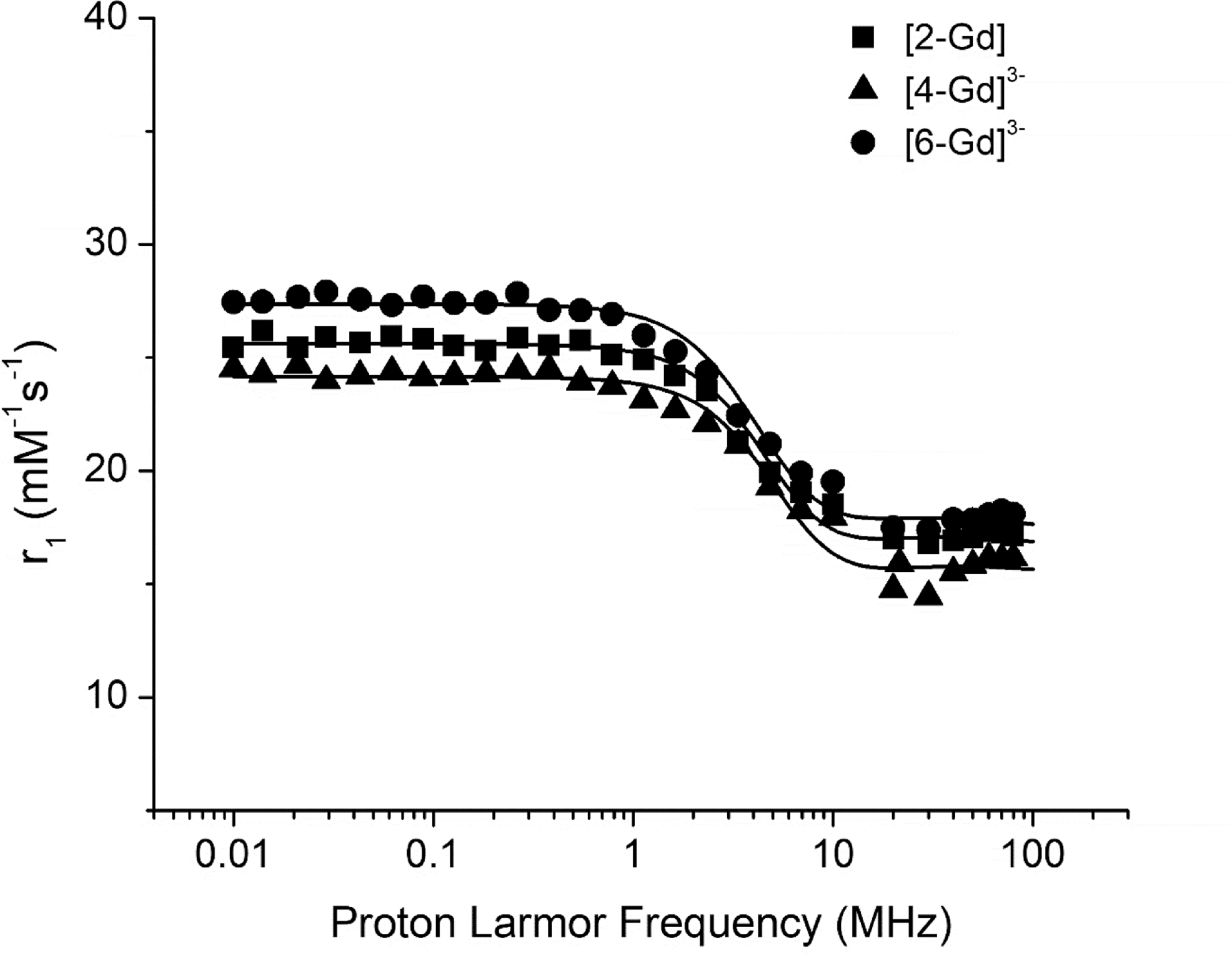
1/*T*_1_ NMRD profiles of [**2**·Gd] (■), [**4**·Gd]^3–^ (▲), and [**6**·Gd]^3–^ (●) recorded at 25 °C in water at neutral pH. The data refer to a concentration of the paramagnetic complex of 1 mM.

The formation of Gd complexes was investigated through the relaxometric titration of a given quantity of ligands (**2**, **4**, and **6**) with increasing amounts of GdCl_3_. The inclusion of Gd^3+^ in the macrocyclic ligands results in the formation of paramagnetic complexes ([**2**·Gd], [**4**·Gd]^3–^, and [**6**·Gd]^3–^) whose relaxivity (*r*_1_) can be calculated from the slope of the straight line obtained by plotting the observed longitudinal relaxation rate (*R*_1obs_ = 1/*T*_1obs_), at 21.5 MHz (proton Larmor frequency) and 25 °C, as a function of increasing concentrations of added GdCl_3._ From this experiment, relaxivity values of 18.3, 15.9, and 20.7 mM^–1^ s^–1^ were determined for [**2**·Gd], [**4**·Gd]^3–^, and [**6**·Gd]^3–^, respectively. The values are significantly lower than those obtained for the Gd complexes with cyclic peptoids reported in our previous study,^26^ likely due to diminished hydration of the paramagnetic metal ion in the inner and/or in the second coordination sphere. In particular, for [*cyclo*-(*N*ce_6_)·Gd]^3–^, a constitutional isomer of **4** and **6** containing a chiral (2-carboxyethyl)glycine units, a value of 31.5 mM^–1^ s^–1^ was determined.^26^ The decreased level of hydration was confirmed by fitting the NMRD (nuclear magnetic relaxation dispersion) profiles, where the relaxivity is measured as a function of the applied magnetic field (Figure 10). The available theory for paramagnetic relaxation, which is based on the Solomon–Bloembergen–Morgan equations, allows for the determination of the principal relaxometric parameters that are responsible for the attained relaxivity; in particular, the number of inner and second sphere water molecules (*q*^is^ and *q*^ss^, respectively), the reorientational correlation time (τ_R_), and the parameters involved in the electronic relaxation (Δ^2^ and τ_V_) have been estimated and are reported in Table 1. For all three complexes, two inner sphere water molecules were determined, while the number of second sphere waters is ∼12.

**Table 1:** Main Relaxometric Parameters Derived from Fitting of NMRD Profiles Reported in Figure 10^a^

	Δ^2^ (s^–2^)^b^	τ_v_^c^ (ps)	τ_R_^d^ (ps)	*q*^e^	*q*_ss_^f^
[**2**·Gd]	(3.3 ± 0.4) × 10^19^	27.4 ± 2.5	168 ± 11	2	11.5 ± 0.5
[**4**·Gd]^3–^	(4.1 ± 1.5) × 10^19^	24.3 ± 6.1	144 ± 13	2	12.0 ± 2.0
[**6**·Gd]^3–^	(2.8 ± 0.4) × 10^19^	29.2 ± 3.1	181 ± 18	2	11.6 ± 0.6

a When the fitting procedure was carried out, some parameters were fixed to reasonable values: *r*_Gd–H_ (distance between Gd and protons of the inner sphere water molecule) = 3.1 Å; *a* (distance of minimum approach of solvent water molecules to the Gd ion) = 3.8 Å; *D* (solvent diffusion coefficient) = 2.2 × 10^–5^ cm^2^ s^–1^.

b Squared mean transient zero-field splitting (ZFS) energy.

c Correlation time for the collision-related modulation of the ZFS Hamiltonian.

d Reorientational correlation time.

e Number of inner sphere water molecules.

f Number of second sphere water molecules.

The values of the reorientational correlation time, as well as those involved in the electronic relaxation, are very similar to each other and to those reported for the other Gd complexes with cyclic peptoids of our previous study.^26^

Analogous to our previous study,^26^ a relaxometric experiment was used for the estimation of the thermodynamic stability of [**2**·Gd], [**4**·Gd]^3–^, and [**6**·Gd]^3–^ complexes. The formation constants for these complexes (*K*_f_) were estimated by carrying out a competition study with another ligand (EDTA) that forms with Gd^3+^ a complex of known *K*_f_ (5.01 × 10^17^)^29^ and relaxivity (*r*_1_ = 7.6 mM^–1^ s^–1^)^30^ (Figure S25). Stability constants of (3.6 ± 4.1) × 10^15^ (log *K*_Gd–L_ = 15.5), (3.2 ± 4.8) × 10^15^ (log *K*_Gd–L_ = 15.5), and (2.1 ± 0.5) × 10^13^ (log *K*_Gd–L_ = 13.3) were found for [**2**·Gd], [**4**·Gd]^3–^, and [**6**·Gd]^3–^, respectively {a value of (8.8 ± 5.1) × 10^15^; a log *K*_Gd–L_ value of 15.9 was recorded for [*cyclo*-(*N*ce_6_)·Gd]^3–^}.^26^ The obtained values are comparable to or lower than those obtained in our previous study^26^ and thus not compatible with a possible application as MRI contrast agents *in vivo*.

**Figure 11:**
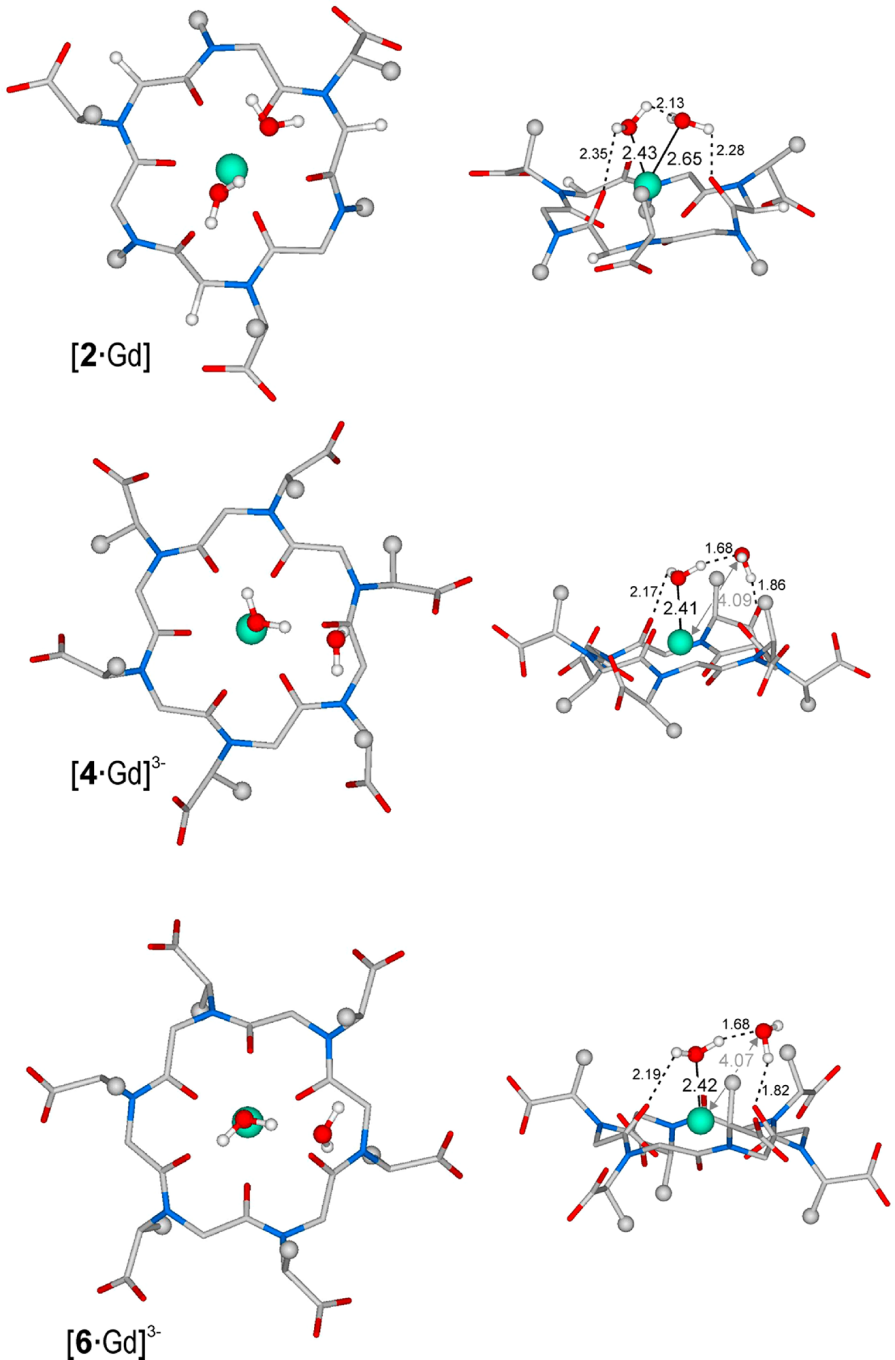
Top and side views of minimum energy structures for gadolinium complexes [**2**·Gd], [**4**·Gd]^3–^, and [**6**·Gd]^3–^ in the presence of two water molecules at the PBE0/6-31G(d,p) level (MWB28 ECP was used for Gd). Hydrogen atoms, except for water molecules, have been omitted for the sake of clarity. Color code: turquoise for Gd^3+^, light gray for C, light blue for N, and red for O (see the Supporting Information for the Cartesian coordinates). Distances are given in angstroms.

In light of experimental results, DFT calculations, at the PBE0/6-31G(d,p) level, on Gd^3+^ cyclic peptoids complexes bearing two water molecules in the inner coordination sphere, were performed to gain additional information about their structures. Minimum energy geometries of [**2**·Gd], [**4**·Gd]^3–^, and [**6**·Gd]^3–^ in the presence of two water molecules are depicted in Figure 11. According to distances reported for the side view of each complex, both water molecules are coordinated directly to the metal in [**2**·Gd] (Gd–OH_2_ distances of 2.43 and 2.65 Å). On the contrary, [**4**·Gd]^3–^ and [**6**·Gd]^3–^ show only one water molecule directly coordinated to gadolinium (*d*_Gd–OH_2__ = 2.41 and 2.42 Å for [**4**·Gd]^3–^ and [**6**·Gd]^3–^, respectively), whereas the second water molecule interacts with the complex mainly by hydrogen bonds with the first water molecule and a carbonyl oxygen of the cyclic peptoid. Differences among [**2**·Gd] and the other complexes can be ascribed to the number of side chains on the cyclic peptoid, which is twice as much for [**4**·Gd]^3–^ and [**6**·Gd]^3–^, significantly crowding the space around the metal. It is worth noting that DFT calculations indicate a frozen state in which in a dynamic contest as in solution can involve a fast exchange between the two water molecules close to the metal.

## Conclusions

We have reported here a rational strategy for the construction of conformationally homogeneous hexameric cyclic peptoids exploiting the subtle interplay among side chain configuration, metal chelation, and general stereoelectronic effects in water. While strategic positioning of chiral (*S*)- and (*R*)-(1-carboxyethyl)glycine units within hexamer peptoid macrocycles allows sodium complexation to enhance their conformational stability, for corresponding octamer peptoid macrocycles, the weaker metal–carbonyl interactions are insufficient to induce conformational homogeneity.

The proper positioning of stereogenic centers is therefore critical for achieving the conformational control of smaller water-soluble cyclic hexa- and tetramers. Although bulky chiral side chains are crucial for gaining predetermined enantiomorphous conformations, they can impede effective complexation with highly hydrated metals such as gadolinium ions. Thus, modest association constants for Gd(III) were observed, requiring additional development of peptoid macrocycles as metal chelators to enable future applications as MRI contrast agents.

Overall, this study has provided a set of design tools for controlling the conformations of oligopeptoids, a necessary step for the programmatic construction of novel water-soluble catalytic/biomimetic polyamide foldamers.

## Experimental Section

### General Methods

Starting materials and reagents purchased from commercial suppliers were generally used without purification unless otherwise mentioned. HPLC analyses were performed on a JASCO LC-NET II/ADC instrument equipped with a JASCO model PU-2089 Plus Pump and a JASCO MD-2010 Plus UV–vis multiple-wavelength detector set at 220 nm. The column used was a C_18_ reversed-phase analytical column (Waters, Bondapak, 10 μm, 125 Å, 3.9 mm × 300 mm) run with linear gradients of ACN (0.1% TFA) in H_2_O (0.1% TFA) over 30 min, at a flow rate of 1.0 mL/min for the analytical runs. High-resolution mass spectra (HRMS) were recorded on a Bruker Solarix XR Fourier transform ion cyclotron resonance mass spectrometer (FTICR-MS) equipped with a 7T magnet, using electrospray ionization (ESI). Yields refer to chromatographically and spectroscopically (^1^H and ^13^C NMR) pure materials. Optical rotations were measured on a digital polarimeter (JASCO P-2000). A sodium lamp (λ = 589 nm) was used as a light source. NMR spectra were recorded on a Bruker DRX 600 (^1^H at 600.13 MHz and ^13^C at 150.90 MHz) or Bruker DRX 400 (^1^H at 400.13 MHz and ^13^C at 100.03 MHz) instrument. Chemical shifts (δ) are reported in parts per million relative to the residual solvent peak (CHCl_3_, δ = 7.26; ^13^CDCl_3_, δ = 77.00; DHO, δ = 4.68). The temperature at 600 MHz was constant and set to 25 °C. NMR spectra of the sodium salts were recorded in a 0.1 M deuterated NaH_2_PO_4_/Na_2_HPO_4_ buffer at pH 8.0; 25 mL stocks were prepared by adding 0.17 mL of 1 M NaH_2_PO_4_ to 2.33 mL of 1 M Na_2_HPO_4_ and then made up to the mark with D_2_O. 1,4-Dioxane was employed as the internal standard for ^13^C NMR acquisition, calibrated at 67.15 ppm. The multiplicity of each signal is designated by the following abbreviations: s, singlet; d, doublet; dd, double doublet; t, triplet; sept, septet; m, multiplet; br, broad. Two-dimensional (2D) NMR experiments such as COSY, ROESY, HSQC, and HMBC were performed for the full assignment of each signal. Coupling constants (*J*) are quoted in hertz.

The longitudinal water proton relaxation rate was measured at 25 °C by using a Stelar Spinmaster (Stelar) spectrometer operating at 21.5 MHz, by means of the standard inversion–recovery technique. The temperature was controlled with a Stelar VTC-91 air flow heater equipped with a copper constantan thermocouple (uncertainty of 0.1 °C).

NMRD profiles were recorded on a Stelar Smartracer Fast-Field-Cycling (FFC) relaxometer at a continuum of proton frequencies from 0.01 to 10 MHz; additional points were obtained between 21.5 and 70 MHz with a Bruker WP80 electromagnet coupled to a Stelar SpinMaster spectrometer. Both systems were equipped with Stelar VTC-91 temperature control, and the internal temperature was checked with a calibrated RS PRO RS55-11 digital thermometer.

### General Procedure for the Solid-Phase Synthesis of Linear Peptoids **8–16**

The 2-chlorotrityl chloride resin (α-dichlorobenzhydryl-polystyrene cross-linked with 1% DVB; 100–200 mesh; 1.71 mmol g^–1^, 0.300 g, 0.513 mmol) was swelled in dry CH_2_Cl_2_ (3 mL) for 45 min and washed twice with dry CH_2_Cl_2_ (3 mL). The first submonomer was attached to the resin by adding bromoacetic acid (0.114 g, 0.821 mmol) in dry CH_2_Cl_2_ (3 mL) and DIPEA (447 μL, 2.56 mmol) on a shaker platform for 60 min at room temperature, followed by washing with DMF (3 × 1 min), with CH_2_Cl_2_ (3 × 1 min), and then again with DMF (3 × 1 min). A solution of the chosen amine (1.6 M in dry DMF, 3 mL) was added to the bromoacetylated resin. Benzyl ester-protected amino acids were employed for the synthesis of compounds **11**–**16**, while *tert*-butyl-protected amino acids were employed for the synthesis of compounds **8**–**10**. The mixture was left on a shaker platform for 60 min at room temperature for the chirally hindered submonomers, 30 min for the achiral submonomers, and then the resin was washed with DMF (3 × 1 min), with CH_2_Cl_2_ (3 × 1 min), and then again with DMF (3 × 1 min). Subsequent bromoacetylation reactions were performed by reacting the aminated oligomer with a solution of bromoacetic acid (0.713 g, 5.13 mmol) and DIC (0.87 mL, 5.64 mmol) in dry DMF (3 mL) for 60 min at room temperature. The filtered resin was washed with DMF (4 × 1 min), CH_2_Cl_2_ (4 × 1 min), and DMF (4 × 1 min) and treated again with the proper amine under the same conditions reported above. This cycle of reactions was iterated until the target linear oligomer was obtained. The cleavage was performed by treating the resin, previously washed with CH_2_Cl_2_ (3 × 1 min), three times with a solution of HFIP in CH_2_Cl_2_ [20% (v/v), 3.0 mL each time] on a shaker platform at room temperature for 30 min each time. The resin was then filtered away, and the combined filtrates were concentrated *in vacuo*. One milligram of the final products was dissolved in 70 μL of acetonitrile (0.1% TFA) and 70 μL of HPLC grade water (0.1% TFA) and analyzed by RP-HPLC [purity of >70%; conditions, 5% to 100% A in 30 min for all oligomers (A, 0.1% TFA in acetonitrile; B, 0.1% TFA in water); flow rate of 1.0 mL min^–1^; 220 nm]. The crude linear oligomers (isolated as amorphous solids) were subjected to ESI mass spectrometry and, subsequently, to the cyclization reactions without further purification.

#### H-[*N*sce_(*t*-Bu)_-Sar]_2_-OH (**8**)

White amorphous solid: 0.261 g, 96% yield; HPLC (C_18_ column, 5% to 100% 0.1% TFA in acetonitrile/water in 30 min, flow rate of 1.0 mL/min, λ = 220 nm) *t*_R_ 7.4 min; HRMS (MALDI/FTICR) *m*/*z* [M + K]^+^ calcd for C_24_H_42_N_4_O_9_K^+^ 569.2583, found 569.2561.

#### H-[*N*sce_(*t*-Bu)_-Sar]_3_-OH (**9**)

White amorphous solid: 0.323 g, 80% yield; HPLC (C_18_ column, 5% to 100% 0.1% TFA in acetonitrile/water in 30 min, flow rate of 1.0 mL/min, λ = 220 nm) *t*_R_ 9.2 min; HRMS (MALDI/FTICR) *m*/*z* [M + K]^+^ calcd for C_36_H_62_N_6_O_13_K^+^ 825.4006, found 825.3974.

#### H-[*N*sce_(*t*-Bu)_-Sar]_4_-OH (**10**)

White amorphous solid: 0.267 g, 50% yield; HPLC (C_18_ column, 5% to 100% 0.1% TFA in acetonitrile/water in 30 min, flow rate of 1.0 mL/min, λ = 220 nm) *t*_R_ 10.2 min; HRMS (MALDI/FTICR) *m*/*z* [M + Na]^+^ calcd for C_48_H_82_N_8_O_17_Na^+^ 1065.5690, found 1065.5628.

#### H-[*N*sce_(Bn)_]_4_-OH (**11**)

White amorphous solid: 0.449 g, 98% yield; HPLC (C_18_ column, 5% to 100% 0.1% TFA in acetonitrile/water in 30 min, flow rate of 1.0 mL/min, λ = 220 nm) *t*_R_ 12.7 min; HRMS (MALDI/FTICR) *m*/*z* [M + K]^+^ calcd for C_48_H_54_N_4_O_13_K^+^ 933.3319, found 933.3272.

#### H-[*N*sce_(Bn)_]_6_-OH (**12**)

White amorphous solid: 0.501 g, 73% yield; HPLC (C_18_ column, 5% to 100% 0.1% TFA in acetonitrile/water in 30 min, flow rate of 1.0 mL/min, λ = 220 nm) *t*_R_ 16.7 min; HRMS (MALDI/FTICR) *m*/*z* [M + H]^+^ calcd for C_72_H_81_N_6_O_19_^+^ 1333.5551, found 1333.5505.

#### H-[*N*sce_(Bn)_]_8_-OH (**13**)

White amorphous solid: 0.645 g, 71% yield; HPLC (C_18_ column, 5% to 100% 0.1% TFA in acetonitrile/water in 30 min, flow rate of 1.0 mL/min, λ = 220 nm) *t*_R_ 16.3 min; HRMS (MALDI/FTICR) *m*/*z* [M + Na]^+^ calcd for C_96_H_106_N_8_O_25_Na^+^ 1793.7161, found 1793.7144.

#### H-[*N*rce_(Bn)_-*N*sce_(Bn)_]_2_-OH (**14**)

White amorphous solid: 0.440 g, 96% yield; HPLC (C_18_ column, 5% to 100% 0.1% TFA in acetonitrile/water in 30 min, flow rate of 1.0 mL/min, λ = 220 nm) *t*_R_ 11.9 min; HRMS (MALDI/FTICR) *m*/*z* [M + K]^+^ calcd for C_48_H_54_N_4_O_13_K^+^ 933.3319, found 933.3360.

#### H-[*N*rce_(Bn)_-*N*sce_(Bn)_]_3_-OH (**15**)

White amorphous solid: 0.588 g, 86% yield; HPLC (C_18_ column, 5% to 100% 0.1% TFA in acetonitrile/water in 30 min, flow rate of 1.0 mL/min, λ = 220 nm) *t*_R_ 12.7 min; HRMS (MALDI/FTICR) *m*/*z* [M + Na]^+^ calcd for C_72_H_80_N_6_O_19_Na^+^ 1355.5370, found 1355.5409.

#### H-[*N*rce_(Bn)_-*N*sce_(Bn)_]_4_-OH (**16**)

White amorphous solid: 0.581 g, 64% yield; HPLC (C_18_ column, 5% to 100% 0.1% TFA in acetonitrile/water in 30 min, flow rate of 1.0 mL/min, λ = 220 nm) *t*_R_ 14.8 min; HRMS (MALDI/FTICR) *m*/*z* [M + Na]^+^ calcd for C_96_H_106_N_8_O_25_Na^+^ 1793.7161, found 1793.7110.

### General Procedure for the High-Dilution Cyclization to Yield **17**–**23**

Solutions of linear peptoids (0.150 mmol), previously co-evaporated three times with toluene, were prepared under nitrogen in dry DMF (5.0 mL). The mixture was added dropwise to a stirred solution of HATU (0.228 g, 0.600 mmol) and DIPEA (162 μL, 0.930 mmol) in dry DMF (45.0 mL) by a syringe pump in 3 h, at room temperature under an anhydrous atmosphere. After 12 h, the resulting mixture was concentrated *in vacuo*, diluted with CH_2_Cl_2_ (30 mL), and washed twice with a solution of HCl (1.0 M, 15 mL). The organic phase was washed with water (30.0 mL), dried over anhydrous MgSO_4_, filtered, and concentrated *in vacuo*. Attempts to cyclize oligomers **11** and **14** reacting the mixture at 50 °C did not yield the expected *cyclo*-[*N*sce_(Bn)4_] and *cyclo*-[(*N*rce_(Bn)_-*N*sce_(Bn)_)_2_] macrocycles. The crude peptoids were analyzed via ^1^H NMR. Crude cyclic peptoids **17**, **18**, and **22** were dissolved in hot acetonitrile and precipitated by slowly cooling the acetonitrile solutions. Crude **19**–**21** and **23** were purified on reverse silica gel (C_18_) [conditions, 10% to 100% A (A, acetonitrile; B, water)]. Cyclic peptoids were dissolved in 50% acetonitrile in HPLC grade water and analyzed by RP-HPLC [purity of >90%; conditions, 5% to 100% A in 30 min (A, 0.1% TFA in acetonitrile; B, 0.1% TFA in water); flow rate of 1 mL min^–1^; 220 nm].

#### *cyclo*-[(*cis*,_p_*R*)*N*sce_(*t*-Bu)_-(*trans*,_p_*R*)Sar-(*cis*,_p_*S*)*N*sce_(*t*-Bu)_-(*trans*,_p_*R*)Sar] (**17**)

White amorphous solid: 0.035 g, 53% yield; HPLC (C_18_ column, 5% to 100% 0.1% TFA in acetonitrile/water in 30 min, flow rate of 1.0 mL/min, λ = 220 nm) *t*_R_ 6.5 min; ^1^H NMR (600 MHz, CDCl_3_) δ 5.41 (1H, d, *J* = 14.8 Hz, C*H*HNCH_3_), 5.33 (1H, d, *J* = 15.0 Hz, C*H*HNCH_3_), 4.92 (1H, q, *J* = 7.7 Hz, NC*H*CH_3_), 4.41 (1H, d, *J* = 17.8 Hz, C*H*HNCHCH_3_), 4.32 (1H, d, *J* = 18.4 Hz, C*H*HNCHCH_3_), 3.91 (1H, d, *J* = 18.4 Hz, CH*H*NCHCH_3_), 3.87 (1H, d, *J* = 17.8 Hz, CH*H*NCHCH_3_), 3.78 (1H, q, *J* = 6.9 Hz, NC*H*CH_3_), 3.35 (1H, d, *J* = 14.8 Hz, CH*H*NCH_3_), 3.28 (1H, d, *J* = 15.0 Hz, CH*H*NCH_3_), 2.92 (3H, s, NC*H*_*3*_), 2.88 (3H, s, NC*H*_*3*_), 1.63 (3H, d, *J* = 6.9 Hz, NC*H*CH_3_), 1.45 (18H, s, 2 × COOC(C*H*_*3*_)*_3_*), 1.39 (3H, d, *J* = 7.7 Hz, NC*H*CH_3_); ^13^C{^1^H} NMR (150 MHz, CDCl_3_) δ 171.8 (*C*=OO*t*Bu), 170.6 (*C*=ONCHCH_3_), 169.9 (*C*=OO*t*Bu), 169.5 (*C*=ONCHCH_3_), 167.5 (*C*=ONCH_3_), 167.0 (*C*=ONCH_3_), 81.9 (COO*C*(CH_3_)_3_), 81.7 (COO*C*(CH_3_)_3_), 60.4 (N*C*HCH_3_), 53.9 (N*C*HCH_3_), 51.9 (*C*H_2_NCHCH_3_), 51.2 (*C*H_2_NCH_3_), 51.0 (*C*H_2_NCH_3_), 46.4 (*C*H_2_NCHCH_3_), 33.6 (N*C*H_3_), 33.5 (N*C*H_3_), 28.0 (COOC(*C*H_3_)_3_), 27.9 (COOC(*C*H_3_)_3_), 14.6 (NCH*C*H_3_), 14.3 (NCH*C*H_3_); HRMS (MALDI/FTICR) *m*/*z* [M + H]^+^ calcd for C_24_H_41_N_4_O_8_^+^ 513.2919, found 513.2879.

#### *cyclo*-[*N*sce_(*t*-Bu)_-Sar]_3_ (**18**)

White amorphous solid: 0.036 g, 31% yield; HPLC (C_18_ column, 5% to 100% 0.1% TFA in acetonitrile/water in 30 min, flow rate of 1.0 mL/min, λ = 220 nm) *t*_R_ 9.1 min; ^1^H NMR (600 MHz, CDCl_3_, mixture of conformational isomers) δ 4.89–3.50 (15H, overlapping signals, NC*H*_*2*_ and NC*H*CH_3_), 3.41–2.78 (9H, overlapping s, NC*H*_*3*_), 1.48–1.40 (36H, overlapping signals, NCHC*H*_*3*_ and COO(C*H*_*3*_)*_3_*); ^13^C{^1^H} NMR (150 MHz, CDCl_3_, mixture of conformational isomers) δ 171.6, 170.9, 170.4, 170.0, 169.7, 169.2, 168.8, 167.5, 167.1, 81.8, 81.2, 81.0, 55.9, 55.4, 55.0, 53.0, 52.6, 52.1, 51.3, 51.0, 50.7, 50.2, 47.9, 46.7, 46.3, 45.8, 45.3, 37.9, 36.8, 36.4, 35.7, 35.2, 34.8, 34.4, 33.6, 32.9, 29.7, 15.9, 15.4, 14.8, 14.5; HRMS (MALDI/FTICR) *m*/*z* [M + Na]^+^ calcd for C_36_H_60_N_6_O_12_Na^+^ 791.4161, found 791.4125.

#### *cyclo*-[*N*sce_(*t*-Bu)_-Sar]_4_ (**19**)

White amorphous solid: 0.071 g, 46% yield; HPLC (C_18_ column, 5% to 100% 0.1% TFA in acetonitrile/water in 30 min, flow rate of 1.0 mL/min, λ = 220 nm) *t*_R_ 14.8 min; ^1^H NMR (600 MHz, CDCl_3_, mixture of conformational isomers) δ 5.33–3.32 (20H, overlapping signals, NC*H*_*2*_ and NC*H*CH_3_), 3.24–3.08 (12H, overlapping s, NC*H*_*3*_), 1.60–1.39 (48H, overlapping signals, NCHC*H*_*3*_ and COO(C*H*_*3*_)*_3_*); ^13^C{^1^H} NMR (150 MHz, CDCl_3_, mixture of conformational isomers) δ 172.1, 169.8, 169.1, 168.6, 118.3, 116.3, 116.0, 114.9, 114.5, 113.7, 113.2, 112.8, 112.5, 83.3, 82.9, 81.9, 55.2, 55.0, 52.6, 52.4, 52.1, 51.6, 51.1, 50.9, 50.8, 50.1, 45.4, 44.3, 37.6, 37.1, 36.9, 36.7, 36.4, 36.2, 35.8, 35.6, 14.8; HRMS (MALDI/FTICR) *m*/*z* [M + Na]^+^ calcd for C_48_H_80_N_8_O_16_Na^+^ 1047.5584, found 1047.5528.

#### *cyclo*-[*N*sce_(Bn)_]_6_ (**20**)

White amorphous solid: 0.138 g, 70% yield; HPLC (C_18_ column, 5% to 100% 0.1% TFA in acetonitrile/water in 30 min, flow rate of 1.0 mL/min, λ = 220 nm) *t*_R_ 14.4 min; ^1^H NMR (600 MHz, CDCl_3_, mixture of conformational isomers) δ 7.37–7.28 (30 H, broad signals, overlapping, Ar-*H*), 5.21–3.98 (30H, broad signals, overlapping, C*H*_*2*_Ph, C*H*CH_3_, NC*H*_*2*_), 1.60–1.23 (18H, broad signals, overlapping, CHC*H*_*3*_); ^13^C{^1^H} NMR (150 MHz, CDCl_3_, mixture of conformational isomers) δ 172.2, 171.6, 171.1, 170.1, 169.8, 169.6, 169.4, 169.2, 169.1, 168.9, 168.6, 167.8, 135.3, 128.6, 128.1, 127.7, 127.0, 68.6, 68.2, 67.9, 67.6, 67.1, 65.4, 54.9, 54.6, 54.1, 53.5, 53.2, 53.1 53.0, 52.4, 52.3, 51.8, 47.0, 46.9, 46.6, 46.4, 46.3, 45.7, 45.6, 45.0, 44.8, 44.4, 15.4, 15.1, 14.8, 14.2; HRMS (MALDI/FTICR) *m*/*z* [M + Na]^+^ calcd for C_72_H_78_N_6_O_18_Na^+^ 1337.5265, found 1337.5323.

#### *cyclo*-[*N*sce_(Bn)_]_8_ (**21**)

White amorphous solid: 0.105 g, 40% yield; HPLC (C_18_ column, 5% to 100% 0.1% TFA in acetonitrile/water in 30 min, flow rate of 1.0 mL/min, λ = 220 nm) *t*_R_ 18.5 min; ^1^H NMR (600 MHz, CDCl_3_, mixture of conformational isomers) δ 7.40–7.28 (40H, broad signals, overlapping, Ar-*H*), 5.36–3.95 (40H, broad signals, overlapping, C*H*_*2*_Ph, C*H*CH_3_, NC*H*_*2*_), 1.50–1.18 (24H, broad signals, overlapping, CHC*H*_*3*_); ^13^C{^1^H} NMR (150 MHz, CDCl_3_, mixture of conformational isomers) δ 172.1, 171.9, 171.8, 171.6, 171.4, 171.0, 170.7, 170.5, 169.9, 169.8, 169.6, 169.5, 168.9, 168.6, 168.4, 167.8, 167.6, 167.4, 167.3, 167.0, 160.7, 160.5, 160.2, 159.9, 135.4, 135.3, 128.6, 128.1, 127.7, 127.0, 68.4, 67.9, 67.7, 67.1, 66.8, 65.4, 58.0, 57.8, 57.6, 57.1, 55.9, 55.6, 55.4, 55.2, 54.6, 54.5, 53.4, 53.2, 53.1, 52.9, 52.7, 52.4, 52.2, 52.1, 47.1, 46.8, 46.6, 46.4, 46.3, 46.0, 45.5, 45.2, 45.0, 44.8, 44.6, 44.2, 15.3, 14.9, 14.1, 13.9; HRMS (MALDI/FTICR) *m*/*z* [M + Na]^+^ calcd for C_96_H_104_N_8_O_24_Na^+^ 1775.7056, found 1775.7003.

#### *cyclo*-[*N*rce_(Bn)_-*N*sce_(Bn)_]_3_ (**22**)

White amorphous solid: 0.034 g, 17% yield; HPLC (C_18_ column, 5% to 100% 0.1% TFA in acetonitrile/water in 30 min, flow rate of 1.0 mL/min, λ = 220 nm) *t*_R_ 15.3 min; ^1^H NMR (600 MHz, CDCl_3_, mixture of conformational isomers) δ 7.30–7.22 (30H, overlapping signals, broad, Ar*H*), 5.27–3.10 (30H, overlapping signals, broad, NC*H*_*2*_Ph, NC*H*CH_3_, NC*H*_*2*_), 1.70–1.22 (18H, overlapping signals, NCHC*H*_*3*_); ^13^C{^1^H} NMR (150 MHz, CDCl_3_, mixture of conformational isomers) δ 171.2, 170.8, 170.2, 169.4, 168.2, 135.9, 135.8, 135.5, 128.8, 128.6, 128.4, 128.2, 127.8, 127.6, 67.9, 67.6, 67.2, 67.1, 66.4, 55.2, 54.6, 52.2, 50.9, 47.1, 45.6, 45.2, 14.7, 14.6, 14.4, 14.1, 13.8; HRMS (MALDI/FTICR) *m*/*z* [M + Na]^+^ calcd for C_72_H_78_N_6_O_18_Na^+^ 1337.5265, found 1337.5295.

#### *cyclo*-[*N*rce_(Bn)_-*N*sce_(Bn)_]_4_ (**23**)

White amorphous solid: 0.163 g, 62% yield; HPLC (C_18_ column, 5% to 100% 0.1% TFA in acetonitrile/water in 30 min, flow rate of 1.0 mL/min, λ = 220 nm) *t*_R_ 16.0 min; ^1^H NMR (600 MHz, CDCl_3_, mixture of conformational isomers) δ 7.37–7.19 (40H, broad signals, overlapping, Ar-*H*), 5.18–3.94 (40H, overlapping signals, broad, NC*H*_*2*_Ph, NC*H*CH_3_, NC*H*_*2*_), 1.37–1.16 (24H, overlapping signals, NCHC*H*_*3*_); ^13^C{^1^H} NMR (150 MHz, CDCl_3_, mixture of conformational isomers) δ 173.4, 172.5, 172.3, 171.9, 170.9, 170.8, 170.0, 169.7, 169.6, 169.4, 169.0, 168.7, 168.2, 167.8, 167.4, 164.4, 135.6, 135.5, 135.2, 128.5, 128.2, 127.9, 127.6, 68.2, 67.8, 67.1, 66.9, 66.7, 54.6, 54.3, 53.9, 52.4, 50.9, 46.7, 46.4, 46.2, 45.9, 45.6, 45.3, 45.1, 44.7, 44.4, 16.8, 16.6, 15.2, 14.7, 14.6, 14.2, 14.0, 13.8; HRMS (MALDI/FTICR) *m*/*z* [M + Na]^+^ calcd for C_96_H_104_N_8_O_24_Na^+^ 1775.7056, found 1775.7109.

### General Procedure for the *tert*-Butyl Deprotection of **17**–**19** and Synthesis of Cyclic Peptoids **1**–**3**

Cyclic peptoids **17**–**19** (0.05 mmol) were dissolved in dry DCM (360 μL); trifluoroacetic acid (200 μL) was then added while the mixture was being stirred. The mixture was stirred at room temperature for 3 h, and then the solution was added dropwise to cold diethyl ether to induce the precipitation of the products as white solids. The compounds were dissolved in a 0.1 M deuterated NaH_2_PO_4_/Na_2_HPO_4_ buffer at pH 8.0.

#### *cyclo*-[(*cis*,_p_*R*)*N*sce-(*trans*,_p_*R*)Sar-(*cis*,_p_*S*)*N*sce-(*trans*,_p_*R*)Sar] (**1**)

White amorphous solid: 0.015 g, 75% yield; HPLC (C_18_ column, 5% to 100% 0.1% TFA in acetonitrile/water in 30 min, flow rate of 1.0 mL/min, λ = 220 nm) *t*_R_ 3.8 min. [**1**·2Na]: ^1^H NMR (600 MHz, 0.1 M deuterated NaH_2_PO_4_/Na_2_HPO_4_ buffer at pH 8.0, single conformational isomer) δ 5.40 (1H, d, *J* = 15.0 Hz, C*H*HNCH_3_), 5.33 (1H, d, *J* = 15.0 Hz, C*H*HNCH_3_), 4.64 (1H, q, *J* = 7.5 Hz, NC*H*CH_3_), 4.44 (1H, d, *J* = 18.3 Hz, C*H*HNCHCH_3_), 4.32 (1H, d, *J* = 18.6 Hz, C*H*HNCHCH_3_), 4.23 (1H, d, *J* = 18.6 Hz, CH*H*NCHCH_3_), 4.17 (1H, d, *J* = 18.3 Hz, CH*H*NCHCH_3_), 3.73 (1H, q, *J* = 7.0 Hz, NC*H*CH_3_), 3.34 (1H, d, *J* = 15.0 Hz, CH*H*NCH_3_), 3.23 (1H, d, *J* = 15.0 Hz, CH*H*NCH_3_), 2.90 (3H, s, NC*H*_*3*_), 2.85 (3H, s, NC*H*_*3*_), 1.60 (3H, d, *J* = 7.0 Hz, NC*H*CH_3_), 1.34 (3H, d, *J* = 7.5 Hz, NC*H*CH_3_); ^13^C{^1^H} NMR (150 MHz, 0.1 M deuterated NaH_2_PO_4_/Na_2_HPO_4_ buffer at pH 8.0) δ 180.0 (*C*OONa), 178.6 (*C*OONa), 173.2 (*C*=O), 171.8 (*C*=O), 169.6 (*C*=O), 169.2 (*C*=O), 63.4 (N*C*HCH_3_), 57.3 (N*C*HCH_3_), 53.9 (*C*H_2_NCHCH_3_), 52.0 (*C*H_2_NCH_3_), 51.5 (*C*H_2_NCH_3_), 47.3 (*C*H_2_NCHCH_3_), 2 × 34.1 (N*C*H_3_), 15.4 (NCH*C*H_3_), 14.9 (NCH*C*H_3_); HRMS (MALDI/FTICR) *m*/*z* [M + Na]^+^ calcd for C_16_H_24_N_4_O_8_Na^+^ 423.1486, found 423.1488; α_D_^25^ −104 (*c* 0.10, 0.1 M deuterated NaH_2_PO_4_/Na_2_HPO_4_ buffer at pH 8.0).

#### *cyclo*-[*N*sce-Sar]_3_ (**2**)

White amorphous solid: 0.023 g, 76% yield; HPLC (C_18_ column, 5% to 100% 0.1% TFA in acetonitrile/water in 30 min, flow rate of 1.0 mL/min, λ = 220 nm) *t*_R_ 5.5 min. [**2**·4Na]^+^: ^1^H NMR (600 MHz, 0.1 M deuterated NaH_2_PO_4_/Na_2_HPO_4_ buffer at pH 8.0, single conformational isomer) δ 4.82 (signal hidden under the HDO signal, 3H, C*H*HNCH_3_), 4.67 (3H, d, *J* = 17.0 Hz, C*H*HNCHCH_3_), 4.46 (3H, q, *J* = 7.2 Hz, NC*H*CH_3_), 3.94 (3H, d, *J* = 17.0 Hz, CH*H*NCHCH_3_), 3.86 (3H, d, *J* = 16.9 Hz, CH*H*NCH_3_), 3.18 (9H, s, NC*H*_*3*_), 1.42 (9H, d, *J* = 7.2 Hz, NCHC*H*_*3*_); ^13^C{^1^H} NMR (150 MHz, 0.1 M deuterated NaH_2_PO_4_/Na_2_HPO_4_ buffer at pH 8.0) δ 3 × 178.4 (*C*OONa), 3 × 172.7 (*C*=ONCH_3_), 3 × 171.0 (*C*=ONCHCH_3_), 3 × 57.9 (N*C*HCH_3_), 3 × 52.4 (*C*H_2_NCHCH_3_), 3 × 46.7 (*C*H_2_NCH_3_), 3 × 34.1 (N*C*H_3_), 3 × 15.4 (NCH*C*H_3_); HRMS (MALDI/FTICR) *m*/*z* [M + K]^+^ calcd for C_24_H_36_N_6_O_12_K^+^ 639.2023, found 639.2021; α_D_^25^ −114 (*c* 0.72, 0.1 M deuterated NaH_2_PO_4_/Na_2_HPO_4_ buffer at pH 8.0).

#### *cyclo*-[*N*sce-Sar]_4_ (**3**)

White amorphous solid: 0.024 g, 60% yield; HPLC (C_18_ column, 5% to 100% 0.1% TFA in acetonitrile/water in 30 min, flow rate of 1.0 mL/min, λ = 220 nm) *t*_R_ 5.8 min. [**3**·5Na]^+^: ^1^H NMR (600 MHz, 0.1 M deuterated NaH_2_PO_4_/Na_2_HPO_4_ buffer at pH 8.0, multiple conformational isomers) δ 5.11–4.90 (6H, overlapping signals, C*H*_*2*_N and NC*H*CH_3_), 4.60–4.33 (11H, overlapping signals, C*H*_*2*_N and NC*H*CH_3_), 4.01–3.66 (3H, overlapping signals, C*H*_*2*_N and NC*H*CH_3_), 3.13–2.88 (12H, overlapping signals, NC*H*_*3*_), 1.48–1.26 (12H, overlapping signals, NCHC*H*_*3*_); ^13^C{^1^H} NMR (150 MHz, 0.1 M deuterated NaH_2_PO_4_/Na_2_HPO_4_ buffer at pH 8.0, multiple conformational isomers) δ 180.8, 180.2, 179.9, 178.6, 178.2, 176.8, 174.9, 174.5, 173.8, 172.8, 172.2, 171.8, 171.0, 170.2, 169.6, 58.0, 57.6, 56.0, 51.9, 51.5, 51.2, 51.0, 46.3, 45.8, 36.5, 36.3, 16.1, 15.7, 14.8; HRMS (MALDI/FTICR) *m*/*z* [M + K]^+^ calcd for C_32_H_48_N_8_O_16_K^+^ 839.2820, found 839.2822; α_D_^25^ −259 (*c* 0.42, 0.1 M deuterated NaH_2_PO_4_/Na_2_HPO_4_ buffer at pH 8.0).

### General Procedure for the Benzyl Deprotection of **20**–**23** and Synthesis of Cyclic Peptoids **4**–**7**

To a solution of cyclic peptoids **20**–**23** (0.030 mmol), in ethyl acetate (0.43 mL), was added 10% wt Pd on carbon (half of the weight with respect to the cyclic peptoid substrate). Three vacuum–hydrogen cycles were performed, and the reaction mixture was stirred for 5 h. After 5 h, 0.86 mL of ethanol was added, and three more vacuum–hydrogen cycles were performed. The reaction mixture was stirred for an additional 19 h, and then the completion of the reaction was assessed via TLC. The reaction mixture was filtered with ethanol on a Celite pad and extensively dried *in vacuo* to obtain the final products. The cyclic peptoids were characterized via ^1^H NMR, ^13^C NMR and HRMS (MALDI-FTICR). The compounds were dissolved in a 0.1 M deuterated NaH_2_PO_4_/Na_2_HPO_4_ buffer at pH 8.0.

#### *cyclo*-[*N*sce]_6_ (**4**)

White amorphous solid: 0.015 g, 65% yield; HPLC (C_18_ column, 5% to 100% 0.1% TFA in acetonitrile/water in 30 min, flow rate of 1.0 mL/min, λ = 220 nm) *t*_R_ 3.5 min. [**4**·7Na]^+^: ^1^H NMR (600 MHz, 0.1 M deuterated NaH_2_PO_4_/Na_2_HPO_4_ buffer at pH 8.0, multiple conformational isomers) δ 4.28–3.53 (18H, broad signals, overlapping, C*H*_*2*_ and C*H*), 1.41–1.14 (18H, broad signals, overlapping, C*H*_*3*_); ^13^C{^1^H} NMR (150 MHz, 0.1 M deuterated NaH_2_PO_4_/Na_2_HPO_4_ buffer at pH 8.0, multiple conformational isomers) δ 179.8, 178.8, 178.6, 178.1, 177.4, 171.6, 171.2, 170.5, 169.4, 58.1, 55.1, 49.5, 49.0, 48.5, 48.3, 47.7, 47.2, 20.3, 17.5, 16.2, 15.1, 14.7, 14.0; HRMS (MALDI/FTICR) *m*/*z* [M + Na]^+^ calcd for C_30_H_42_N_6_O_18_Na^+^ 797.2448, found 797.2446; α_D_^25^ −151 (*c* 0.90, 0.1 M deuterated NaH_2_PO_4_/Na_2_HPO_4_ buffer at pH 8.0).

#### *cyclo*-[*N*sce]_8_ (**5**)

White amorphous solid: 0.024 g, 78% yield; HPLC (C_18_ column, 5% to 100% 0.1% TFA in acetonitrile/water in 30 min, flow rate of 1.0 mL/min, λ = 220 nm) *t*_R_ 3.4 min. [**5**·9Na]^+^: ^1^H NMR (400 MHz, 0.1 M deuterated NaH_2_PO_4_/Na_2_HPO_4_ buffer at pH 8.0, single conformational isomer) δ 4.50–3.71 (24H, overlapping signals, 8 × C*H*_*2*_ and 8 × C*H*), 1.29 (12H, d, *J* = 7.1 Hz, C*H*_*3*_), 1.21 (12H, d, *J* = 7.1 Hz, C*H*_*3*_); ^13^C{^1^H} NMR (150 MHz, 0.1 M deuterated NaH_2_PO_4_/Na_2_HPO_4_ buffer at pH 8.0, single conformational isomer) δ 4 × 180.1 (*C*OONa), 4 × 178.8 (*C*OONa), 4 × 171.5 (*C*=OCH_2_N), 4 × 170.7 (*C*=OCH_2_N), 8 × 58.2 (N*C*HCH_3_), 8 × 55.9 (C=O*C*H_2_N), 4 × 16.4 (NCH*C*H_3_), 4 × 15.2 (NCH*C*H_3_); HRMS (MALDI/FTICR) *m*/*z* [M + K]^+^ calcd for C_40_H_56_N_8_O_24_K^+^ 1071.3039, found 1071.3125; α_D_^25^ −142.1 (*c* 0.79, 0.1 M deuterated NaH_2_PO_4_/Na_2_HPO_4_ buffer at pH 8.0).

#### *cyclo*-[*N*rce-*N*sce]_3_ (**6**)

White amorphous solid: 0.014 g, 61% yield; HPLC (C_18_ column, 5% to 100% 0.1% TFA in acetonitrile/water in 30 min, flow rate of 1.0 mL/min, λ = 220 nm) *t*_R_ 3.6 min. [**6**·7Na]^+^: ^1^H NMR (600 MHz, 0.1 M deuterated NaH_2_PO_4_/Na_2_HPO_4_ buffer at pH 8.0, single conformational isomer) δ 4.66 (6H, d, *J* = 17.4 Hz, C*H*H), 4.56 (6H, q, *J* = 6.9 Hz, NC*H*CH_3_), 3.90 (6H, d, *J* = 17.4 Hz, CH*H*), 1.44 (18H, d, *J* = 6.9 Hz, NCHC*H*_*3*_); ^13^C{^1^H} NMR (150 MHz, 0.1 M deuterated NaH_2_PO_4_/Na_2_HPO_4_ buffer at pH 8.0, single conformational isomer) δ 6 × 178.5 (*C*=OONa), 6 × 171.6 (*C*=O), 6 × 58.1 (N*C*HCH_3_), 6 × 46.9 (*C*H_2_), 6 × 16.1 (NCH*C*H_3_); HRMS (MALDI/FTICR) *m*/*z* [M + Na]^+^ calcd for C_30_H_42_N_6_O_18_Na^+^ 797.2448, found 797.2449; α_D_^25^ −142 (*c* 0.75, 0.1 M deuterated NaH_2_PO_4_/Na_2_HPO_4_ buffer at pH 8.0).

#### *cyclo*-[*N*rce-*N*sce]_4_ (**7**)

White amorphous solid: 0.024 g, 77% yield; HPLC (C_18_ column, 5% to 100% 0.1% TFA in acetonitrile/water in 30 min, flow rate of 1.0 mL/min, λ = 220 nm) *t*_R_ 3.6 min. [**7**·9Na]^+^: ^1^H NMR (600 MHz, 0.1 M deuterated NaH_2_PO_4_/Na_2_HPO_4_ buffer at pH 8.0, single conformational isomer) δ ∼4.70 (signal hidden under the HDO signal, 8H, C*H*H), 4.32 (8H, q, *J* = 7.0 Hz, C*H*CH_3_), 3.72 (8H, d, *J* = 17.4 Hz, C*H*H), 1.24 (24H, d, *J* = 7.0 Hz, CHC*H*_*3*_); ^13^C{^1^H} NMR (150 MHz, 0.1 M deuterated NaH_2_PO_4_/Na_2_HPO_4_ buffer at pH 8.0) δ 8 × 178.7 (*C*=OONa), 8 × 171.6 (*C*=O), 8 × 57.6 (N*C*HCH_3_), 8 × 46.3 (*C*H_2_), 8 × 15.9 (NCH*C*H_3_); HRMS (MALDI/FTICR) *m*/*z* [M + K]^+^ calcd for C_40_H_56_N_8_O_24_K^+^ 1071.3039, found 1071.2938; α_D_^25^ −202 (*c* 0.53, 0.1 M deuterated NaH_2_PO_4_/Na_2_HPO_4_ buffer at pH 8.0).

### X-ray Crystallographic Studies of [**1**·2K]

Single crystals were obtained by the sitting-drop method from the aqueous solution of [**1**·2K]. X-ray diffraction data were collected on a Bruker D8 SMART APEXII three-circle diffractometer system with 0.75 Å resolution. The structure was determined and refined using the Bruker SHELXTL Software Package. The final formula was determined to be C_16_H_34.63_K_2_N_4_O_14.41_ with R1 = 4.85%. Further data are reported in the Supporting Information.

### Complexation of **2**, **4**, and **6** with Gadolinium

[**2**·Gd], [**4**·Gd]^3–^, and [**6**·Gd]^3–^ complexes were synthesized through stepwise addition of 10 μL aliquots of a 10 mM GdCl_3_ solution to solutions of **2**, **4**, and **6**, respectively, at 10 mM, maintaining the pH value at 7 with 0.1 N NaOH.

## Data Availability

The data underlying this study are available in the published article and its online Supporting Information.

## Supporting Information Available

Animations of [**2**·Na]^2–^, [**2a**·Na]^2–^, [**4**·Na]^5–^, [**6**·Na]^5–^, and [**6a**·Na]^5–^ reporting relevant dihedral angles and distances (ZIP)
FAIR data, including the primary NMR FID files, for compounds **1–7** (ZIP)
One-dimensional and 2D spectra of cyclic derivatives **17**–**23** and sodium complexes of **1**–**7**; HPLC chromatograms of linear and cyclic peptoids; graphic of relaxometric competition with EDTA of [**2**·Gd], [**4**·Gd]^3–^, and [**6**·Gd]^3–^; and Cartesian coordinates of [**2**·Na]^2–^, [**2a**·Na]^2–^, [**4**·Na]^5–^, [**6**·Na]^5–^, [**6a**·Na]^5–^, [**2**·Gd], [**4**·Gd]^3–^, and [**6**·Gd]^3–^ (PDF)
