# Supplementary figures and images for: Water-Soluble Chiral Cyclic Peptoids and Their Sodium and Gadolinium Complexes: Study of Conformational and Relaxometric Properties
Source: J Org Chem. 2023 May 8;88(11):6588–98. doi: 10.1021/acs.joc.2c02713 (PMC10242754; doi:10.1021/acs.joc.2c02713)

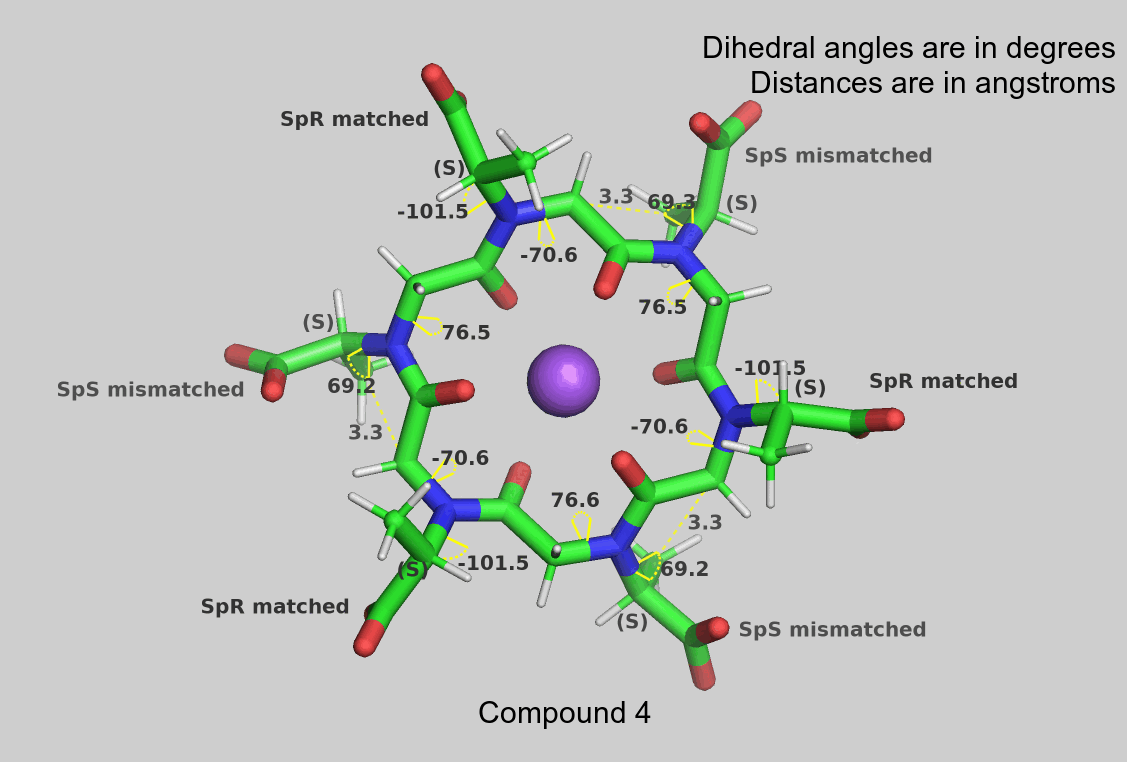

Supplement: Supplementary file 1 — jo2c02713_si_001.zip [file jo2c02713_si_001.zip › Animation esa-4.gif]

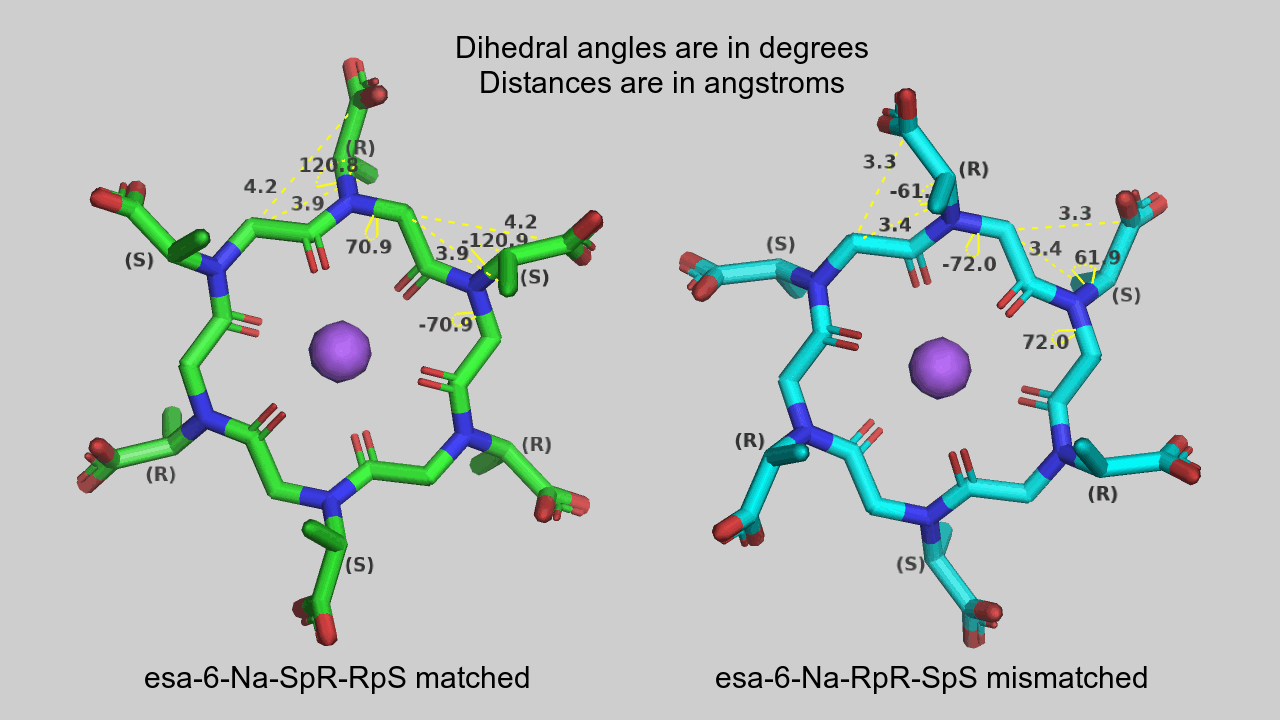

Supplement: Supplementary file 1 — jo2c02713_si_001.zip [file jo2c02713_si_001.zip › Animation esa-6.gif]

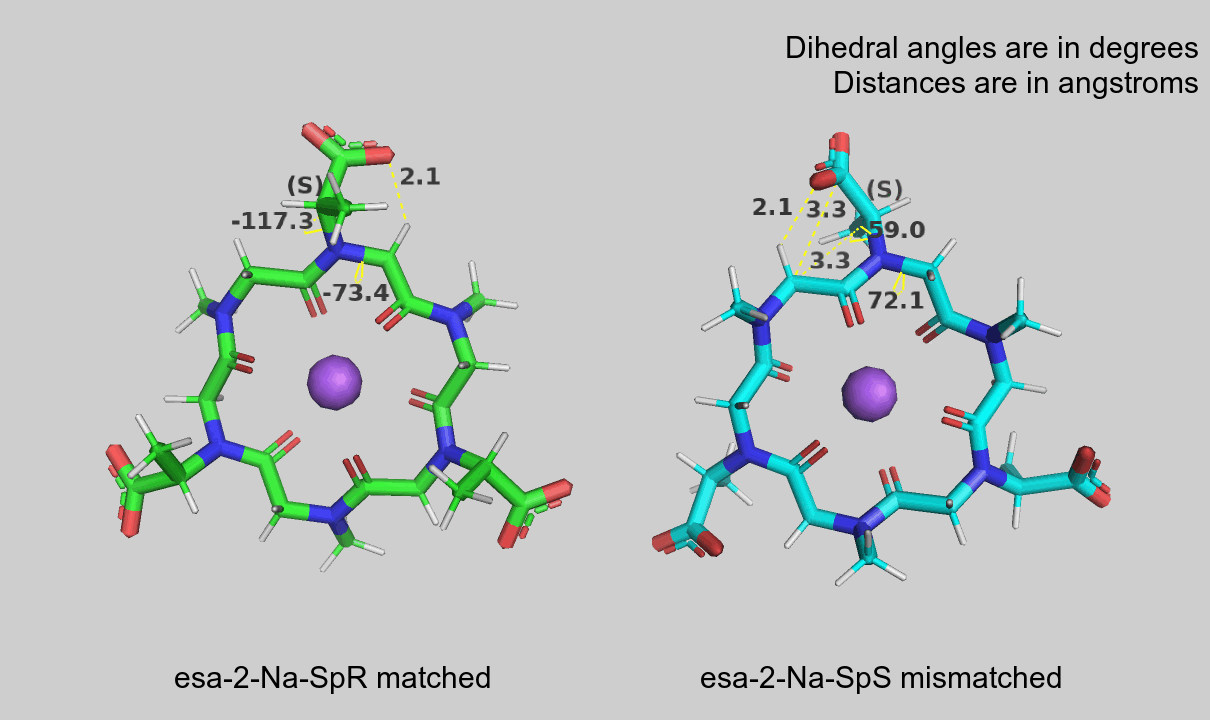

Supplement: Supplementary file 1 — jo2c02713_si_001.zip [file jo2c02713_si_001.zip › Animation esa-2.gif]

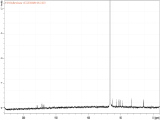

Supplement: Supplementary file 2 — jo2c02713_si_002.zip [file jo2c02713_si_002.zip › FID for publication/1/13C/pdata/1/thumb.png]

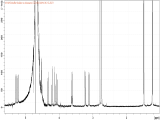

Supplement: Supplementary file 2 — jo2c02713_si_002.zip [file jo2c02713_si_002.zip › FID for publication/1/1H/pdata/1/thumb.png]

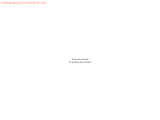

Supplement: Supplementary file 2 — jo2c02713_si_002.zip [file jo2c02713_si_002.zip › FID for publication/1/COSY/pdata/1/thumb.png]

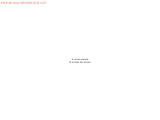

Supplement: Supplementary file 2 — jo2c02713_si_002.zip [file jo2c02713_si_002.zip › FID for publication/1/HMBC/pdata/1/thumb.png]

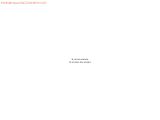

Supplement: Supplementary file 2 — jo2c02713_si_002.zip [file jo2c02713_si_002.zip › FID for publication/1/HSQC/pdata/1/thumb.png]

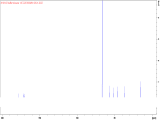

Supplement: Supplementary file 2 — jo2c02713_si_002.zip [file jo2c02713_si_002.zip › FID for publication/2/13C/pdata/1/thumb.png]

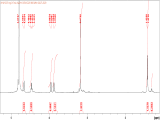

Supplement: Supplementary file 2 — jo2c02713_si_002.zip [file jo2c02713_si_002.zip › FID for publication/2/1H/pdata/1/thumb.png]
