# Supplementary material for: Water-Soluble Chiral Cyclic Peptoids and Their Sodium and Gadolinium Complexes: Study of Conformational and Relaxometric Properties
Source: J Org Chem. 2023 May 8;88(11):6588–98. doi: 10.1021/acs.joc.2c02713 (PMC10242754; doi:10.1021/acs.joc.2c02713)
Supplement: Supplementary file 3 — jo2c02713_si_003.pdf [file jo2c02713_si_003.pdf]

Supporting Information

For

Water-soluble Chiral Cyclic Peptoids and their Sodium and Gadolinium Complexes: Study of Conformational and Relaxometric Properties

Assunta D'Amato,[a] Linhai Jiang,[b] Giorgio Della Sala,[a] Kent Kirshenbaum,[b] Chiara Costabile,[a] Chiara Furlan,[c] Eliana Gianolio,[c] Irene Izzo,[a] Francesco De Riccardis [a,*]

[a] *Department of Chemistry and Biology "A. Zambelli", University of Salerno, Via Giovanni Paolo II, 132, Fisciano (SA), 84084, Italy,*

[b] *Department of Chemistry, New York University, 100 Washington Square East, New York, NY 10003-6688 (USA), and*

[c] *Department of Molecular Biotechnology and Health Sciences and Molecular Imaging Center, University of Turin, Via Nizza 52, 10126, Turin, Italy*

Corresponding Author: Francesco De Riccardis dericca@unisa.it

Index

1.0 List of abbreviations S3

2.0 ^{1}H-, ^{13}C-NMR and two-dimensional spectra S4

3.0 HPLC Chromatograms S41

3.1 HPLC chromatograms of linear peptoids **8-16** as crude mixtures (Figures S1-S9) S41

3.2 HPLC chromatograms of cyclic peptoids **17-23**, **1-7** (Figures S10-S23) S45

4.0 X-ray crystallographic studies of [1·2K] (Fig. S24) **S53**

5.0 Relaxometric competition with EDTA (Fig. S25) **S56**

6.0 Computational details S57

6.1 Cartesian Coordinates and energies of calculated structures S58

1.0 List of abbreviations

ACN: acetonitrile

Ar: aryl

Bn: benzyl

c: cis amide bond

COSY: correlation spectroscopy

DCM: dichloromethane

DIC*:* *N*,*N'*-diisopropylcarbodiimide

DIPEA: *N*,*N*-diisopropylethylamine

DMF: dimethylformamide

ESI: electrospray ionisation

FTICR-MS: Fourier transform ion cyclotron resonance mass spectrometry

HATU: *O*-(7-azabenzotriazol-1-yl)-*N*,*N*,*N'*,*N'*-tetramethyluronium hexafluorophosphate

HFIP: hexafluoroisopropanol

HMBC: heteronuclear multiple bond correlation

HSQC: heteronuclear single quantum correlation

HRMS: high resolution mass spectrometry

Ph: phenyl

ROESY: rotating-frame nuclear Overhauser effect correlation spectroscopy

RP HPLC: reversed-phase high-performance liquid chromatography

t: trans amide bond

TFA: trifluoroacetic acid

TLC: thin layer chromatography

2.0 ¹H-, ¹³C-NMR and two-dimensional spectra

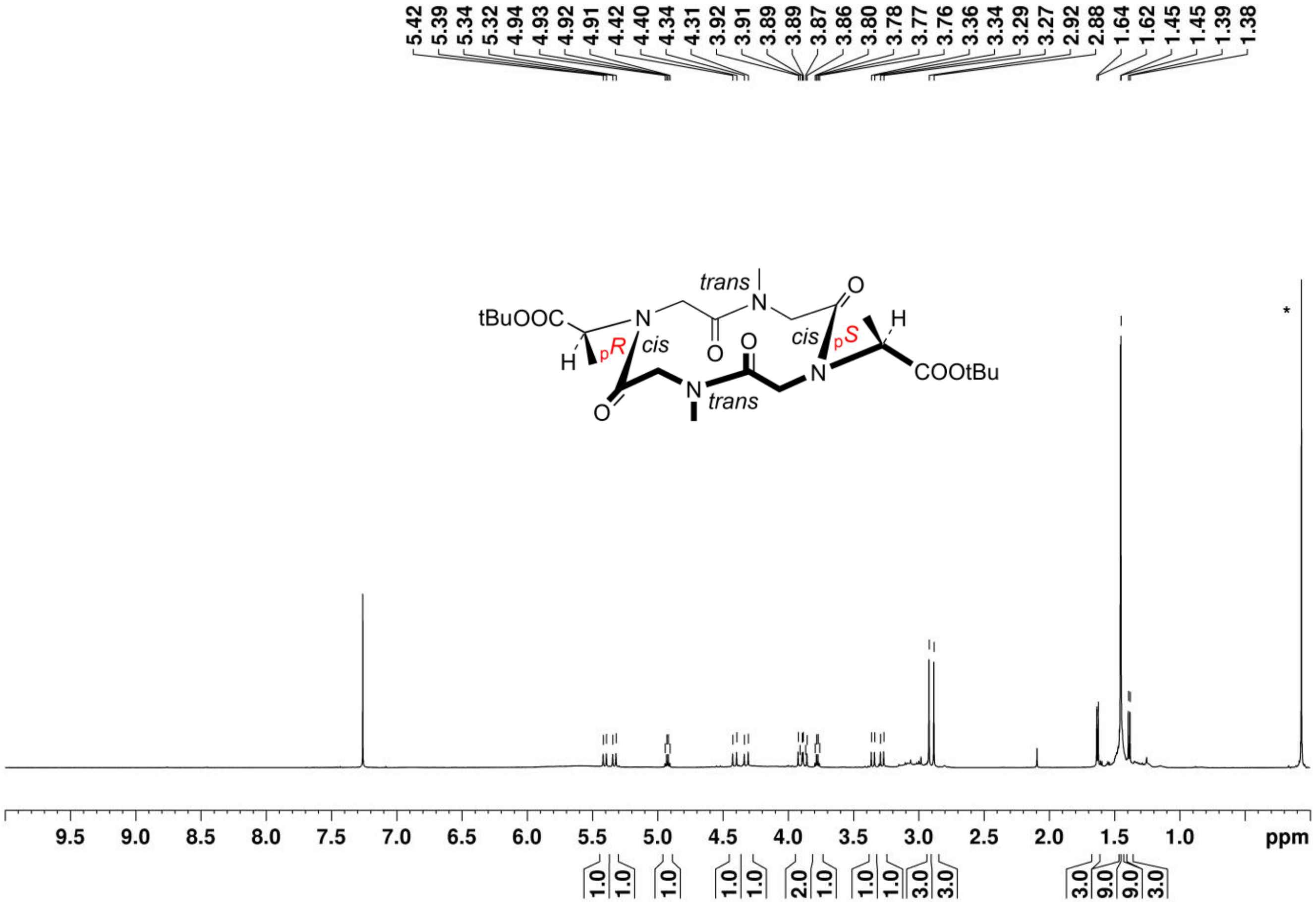

17: ¹H NMR (600 MHz, $CDCl_3$). Silicone grease impurity is marked with a black asterisk.

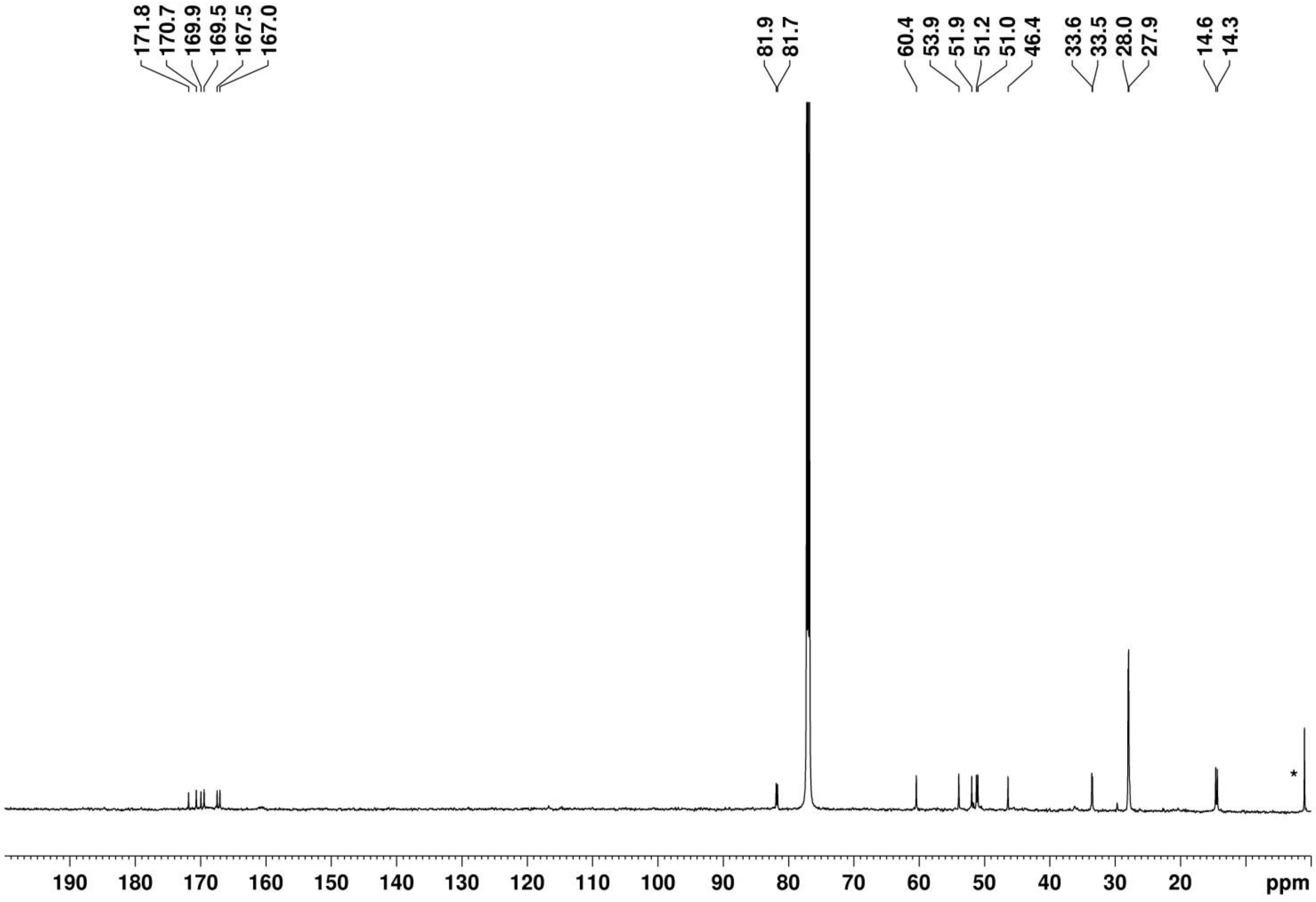

17: 13C{1H} NMR (150 MHz, $CDCl_3$). TMS impurity is marked with a black asterisk.

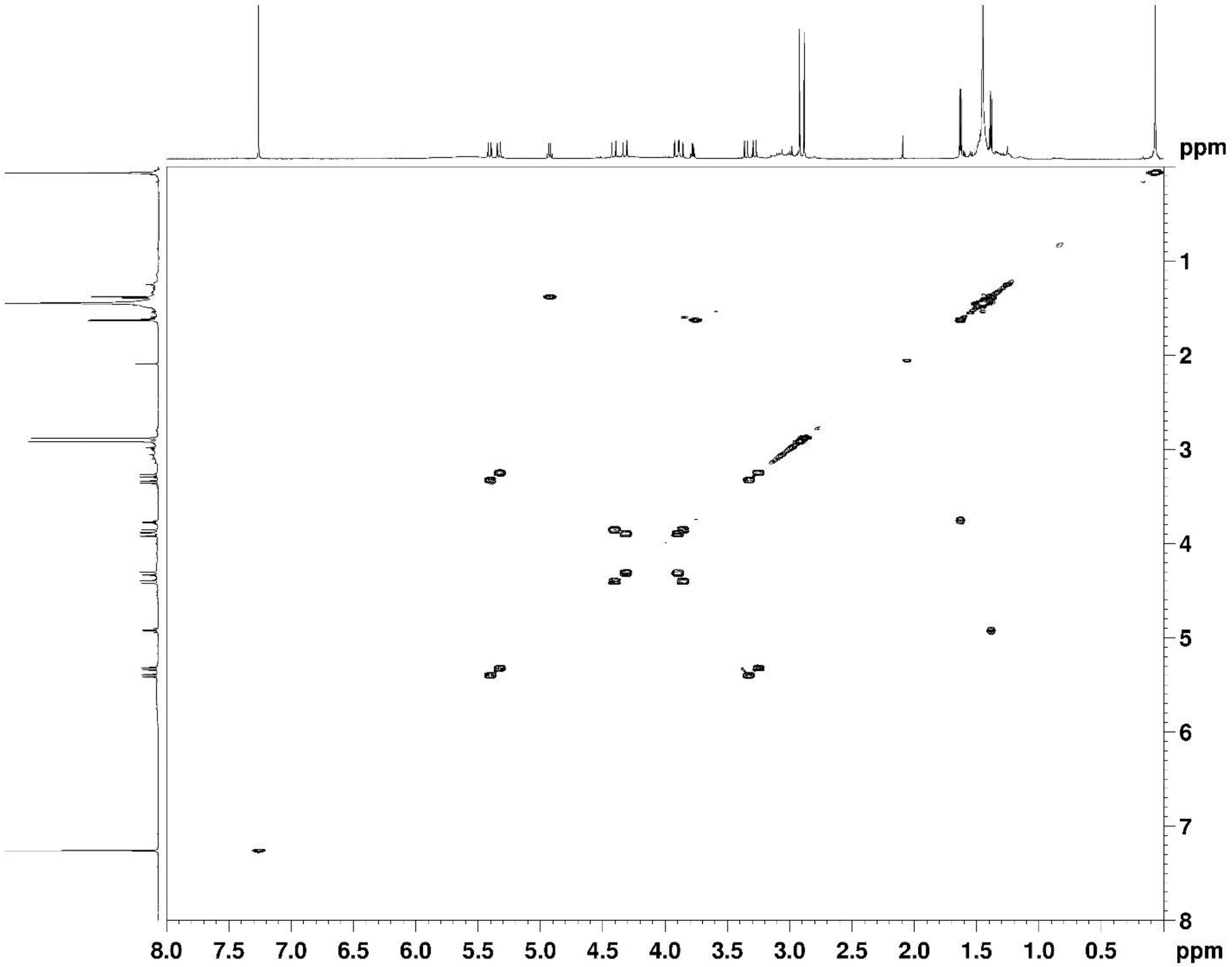

17: COSY SPECTRUM (600 MHz, $CDCl_3$)

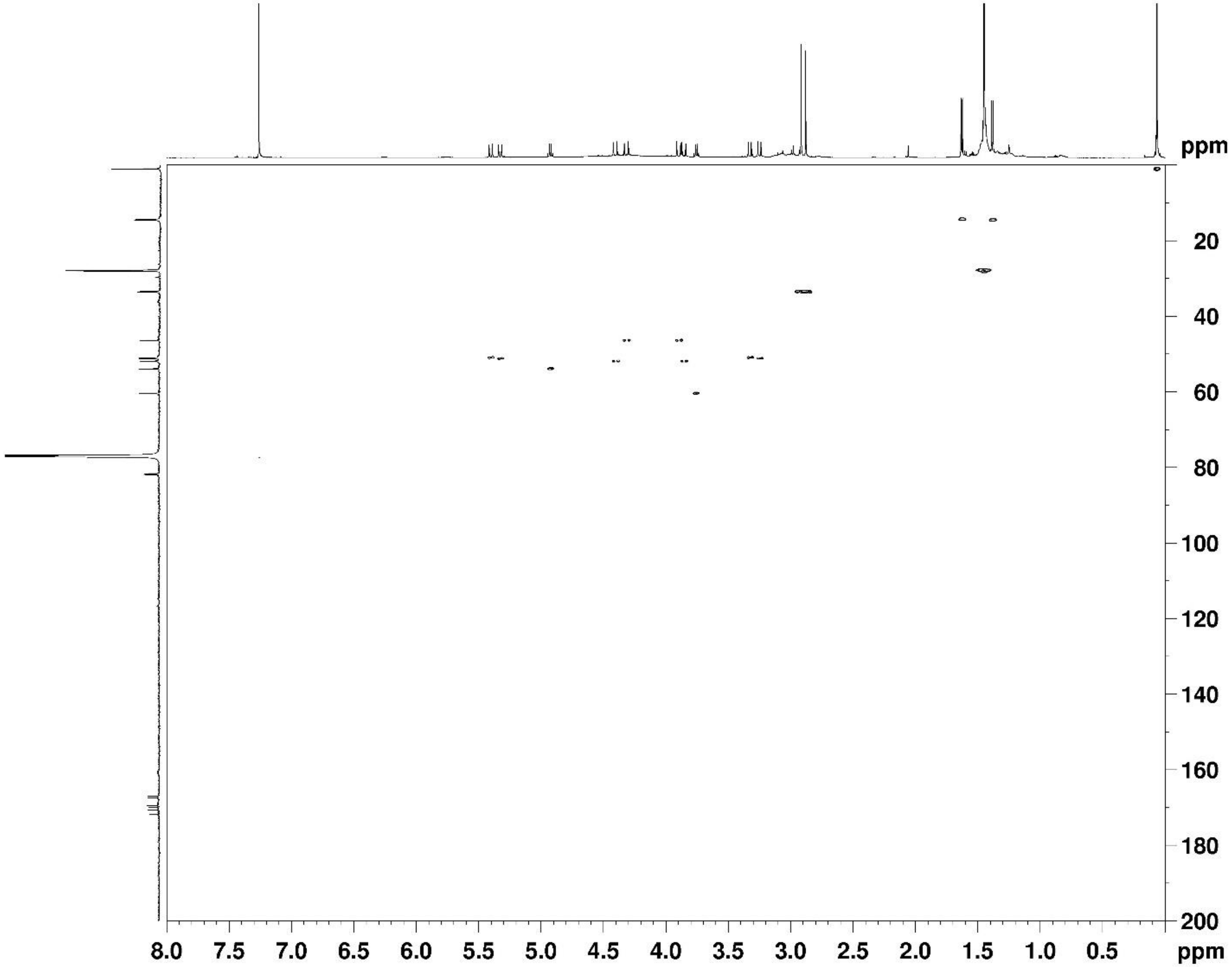

17: HSQC SPECTRUM (600 MHz, $CDCl_3$)

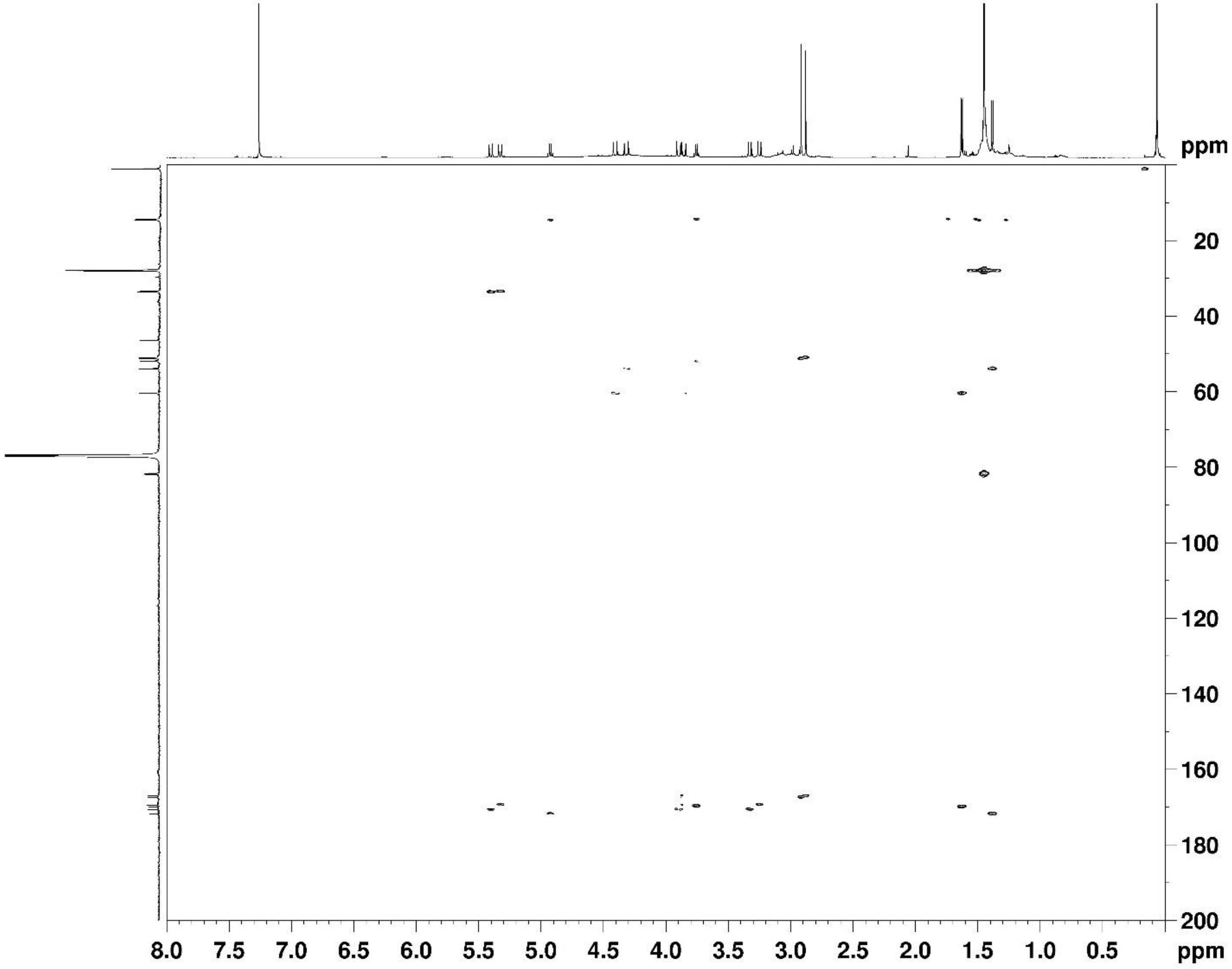

17: HMBC SPECTRUM (600 MHz, $CDCl_3$)

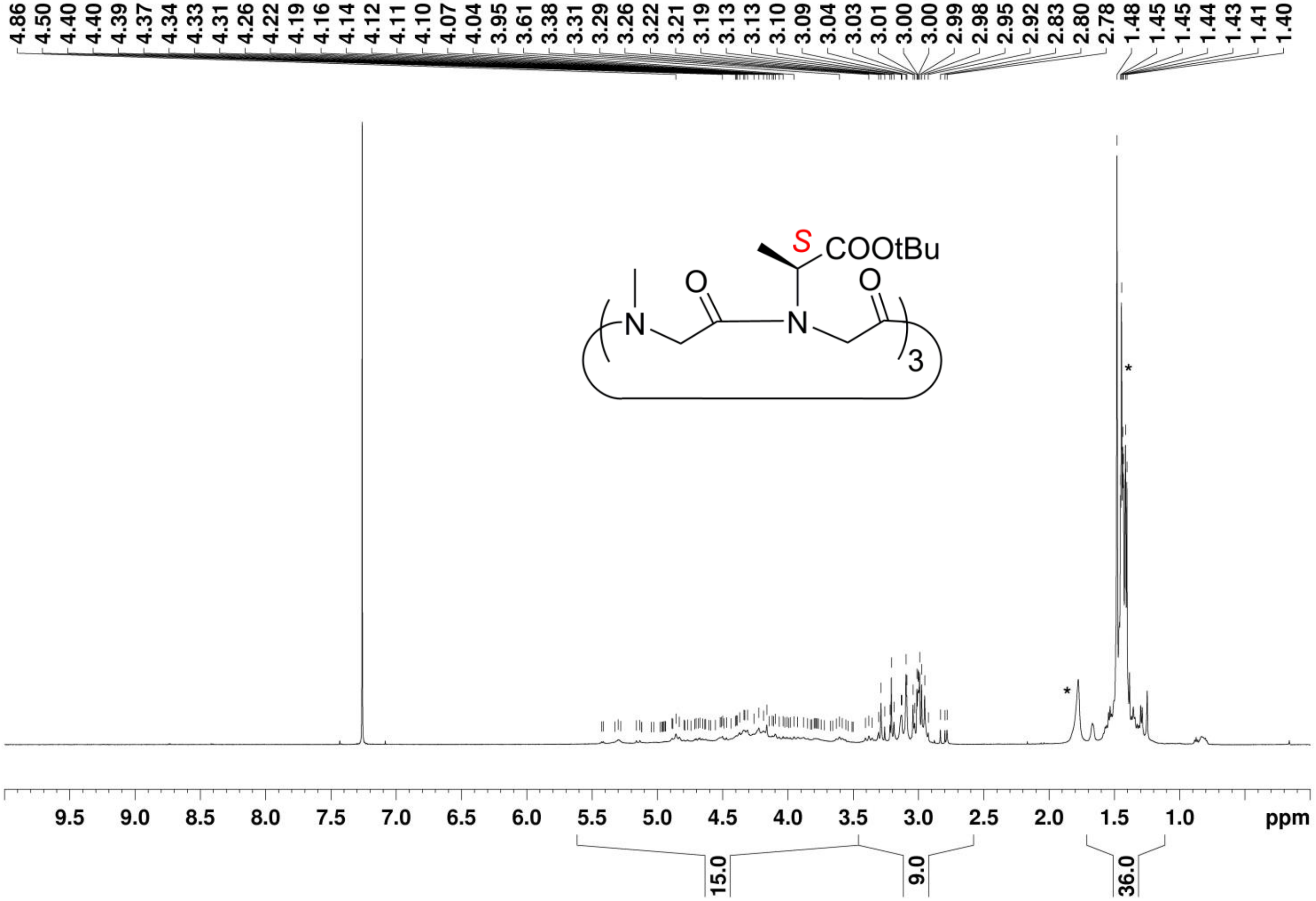

18: ^{1}H NMR (600 MHz, $CDCl_3$). Water and grease impurities are marked with a black asterisk.

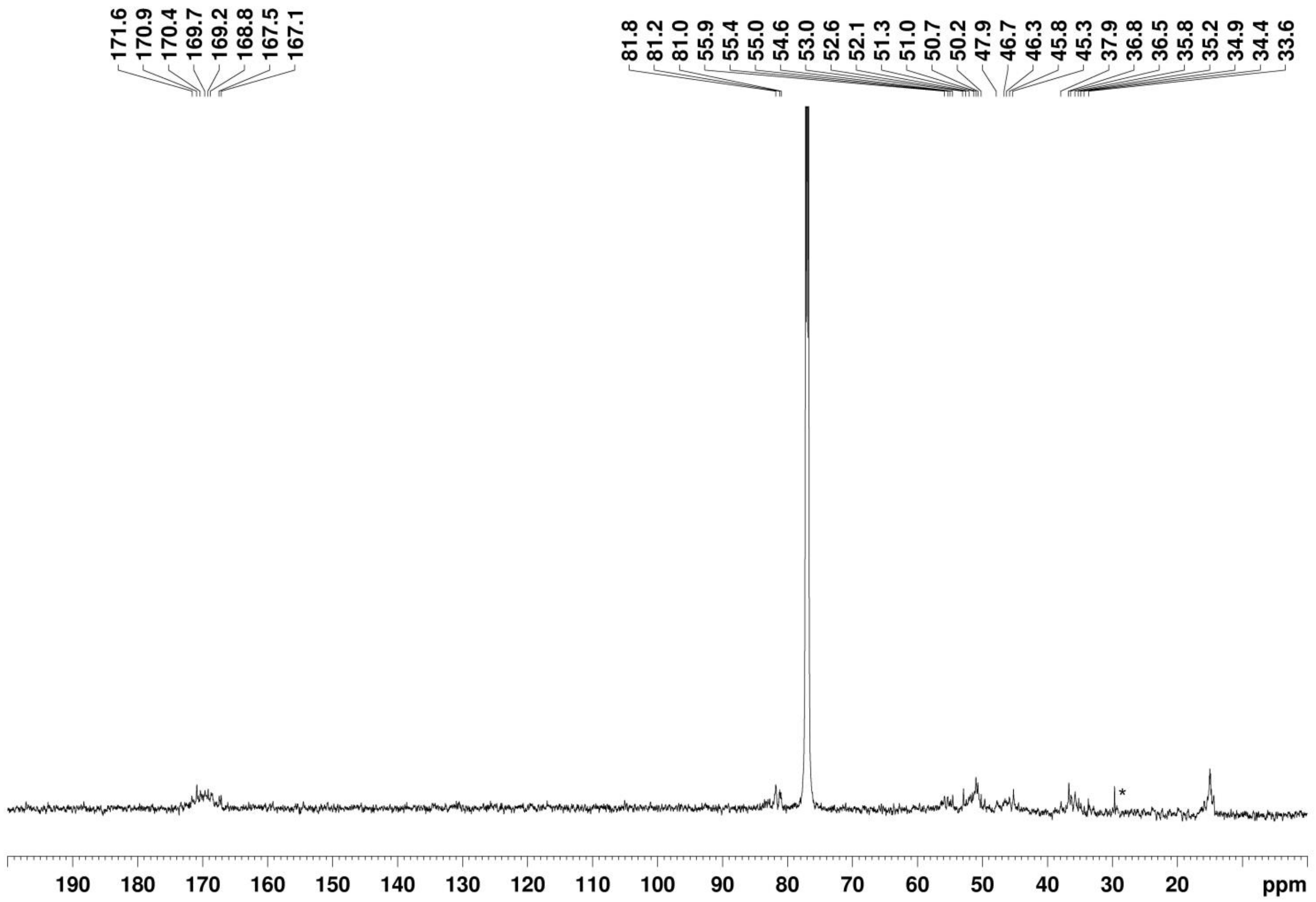

18: 13C{1H} NMR (150 MHz, $CDCl_3$). Grease impurity is marked with a black asterisk.

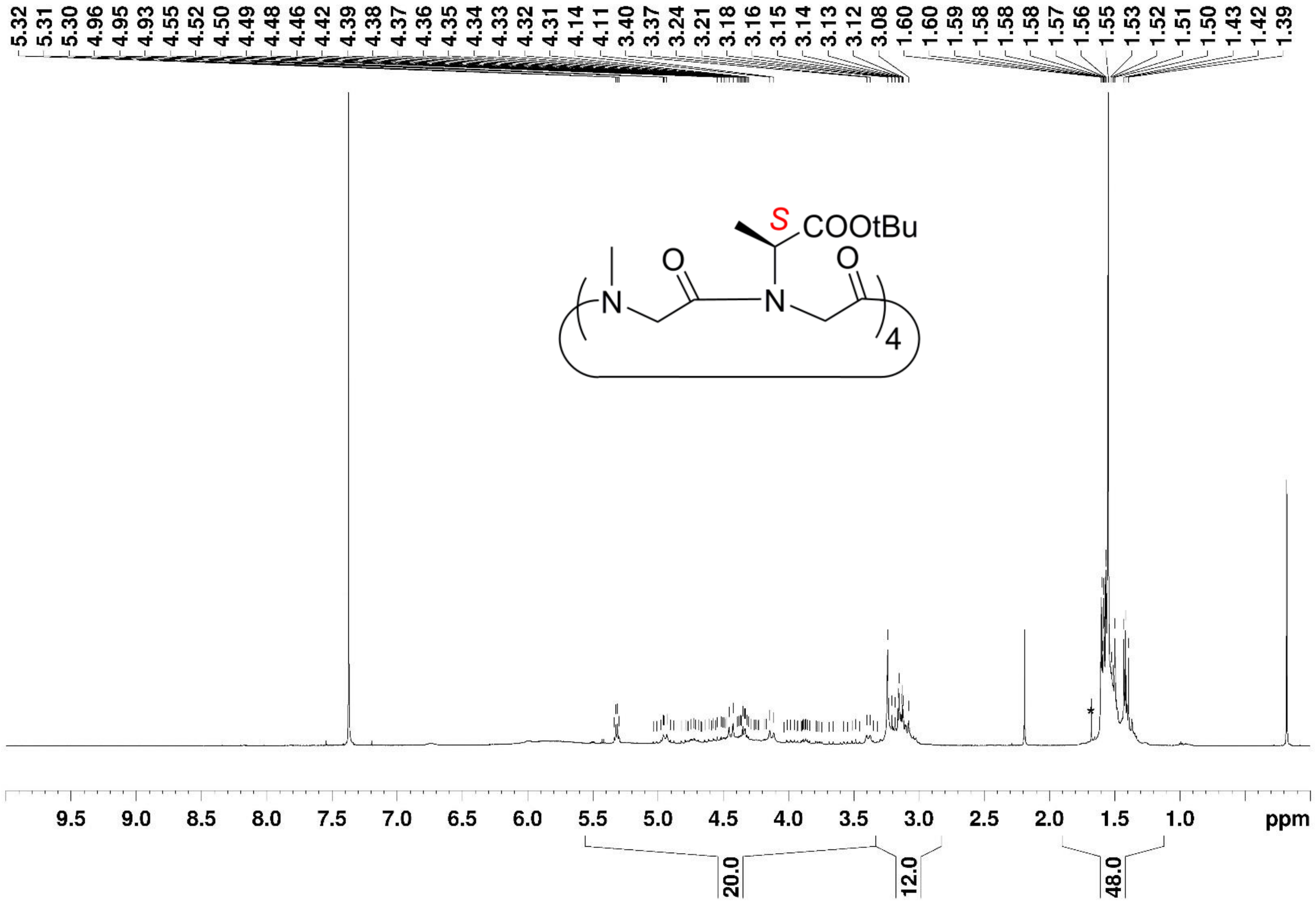

19: ^{1}H NMR (600 MHz, $CDCl_3$)

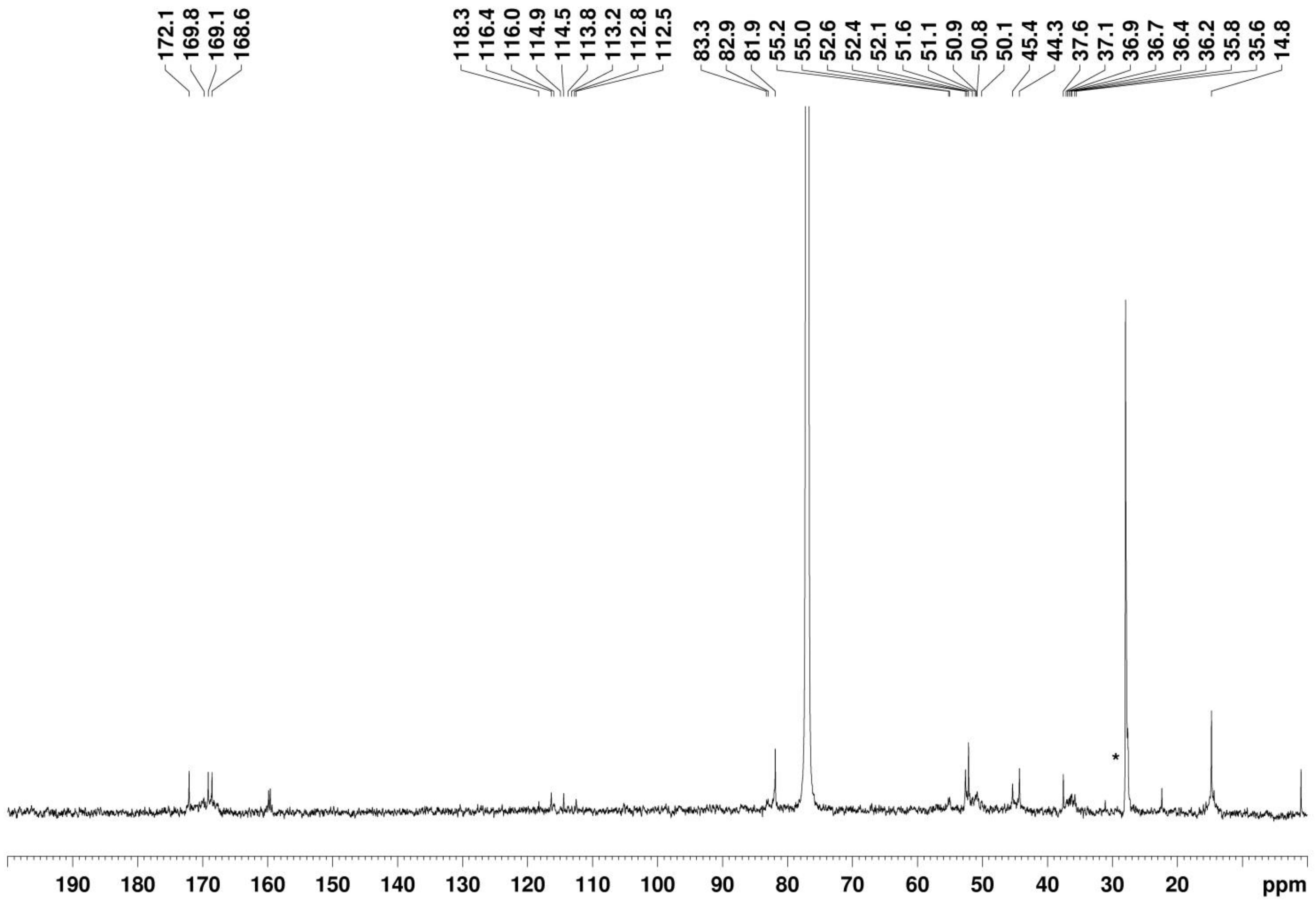

19: 13C{1H} NMR (150 MHz, $CDCl_3$). Grease impurity is marked with a black asterisk.

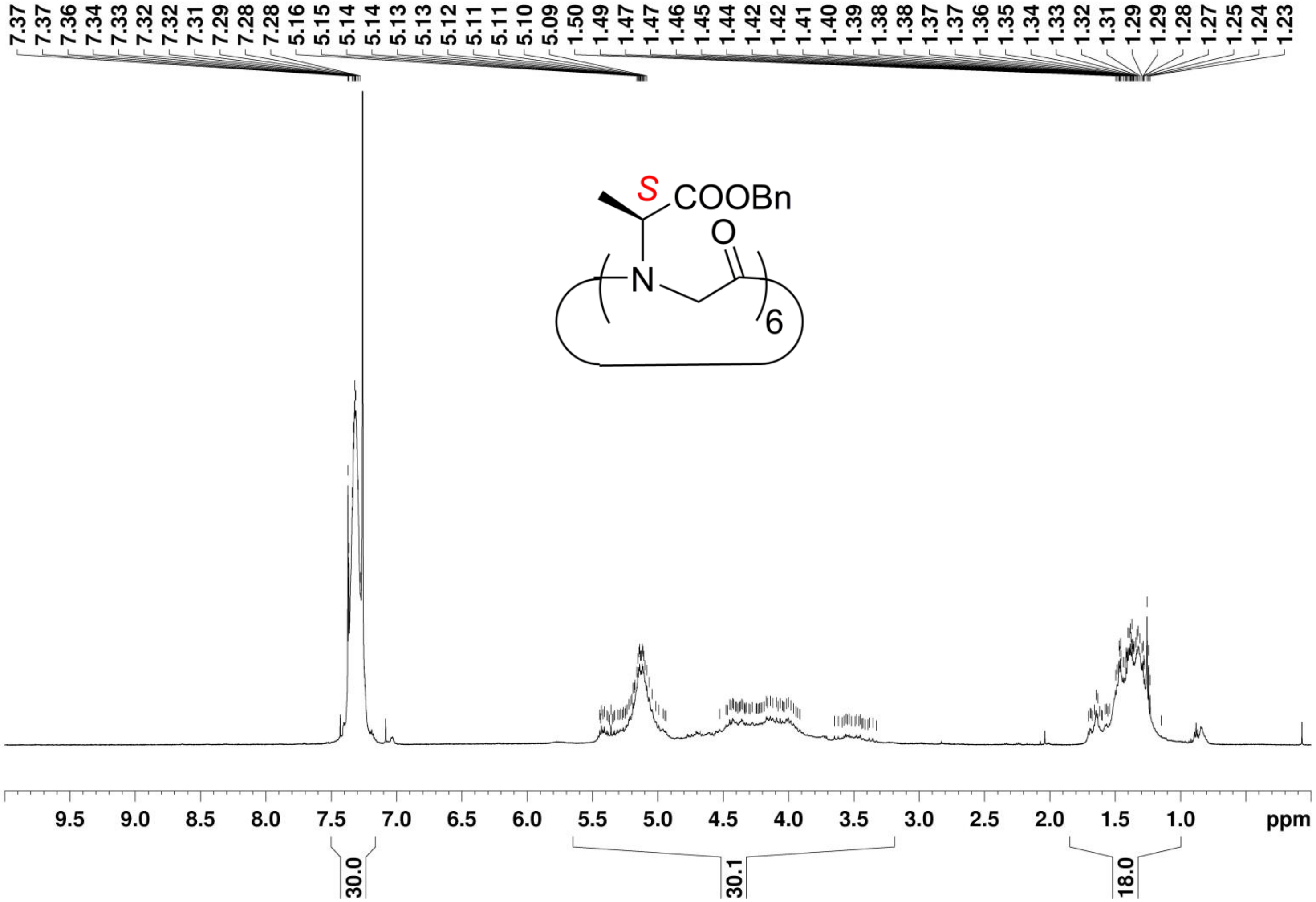

20: ^{1}H NMR (600 MHz, $CDCl_3$)

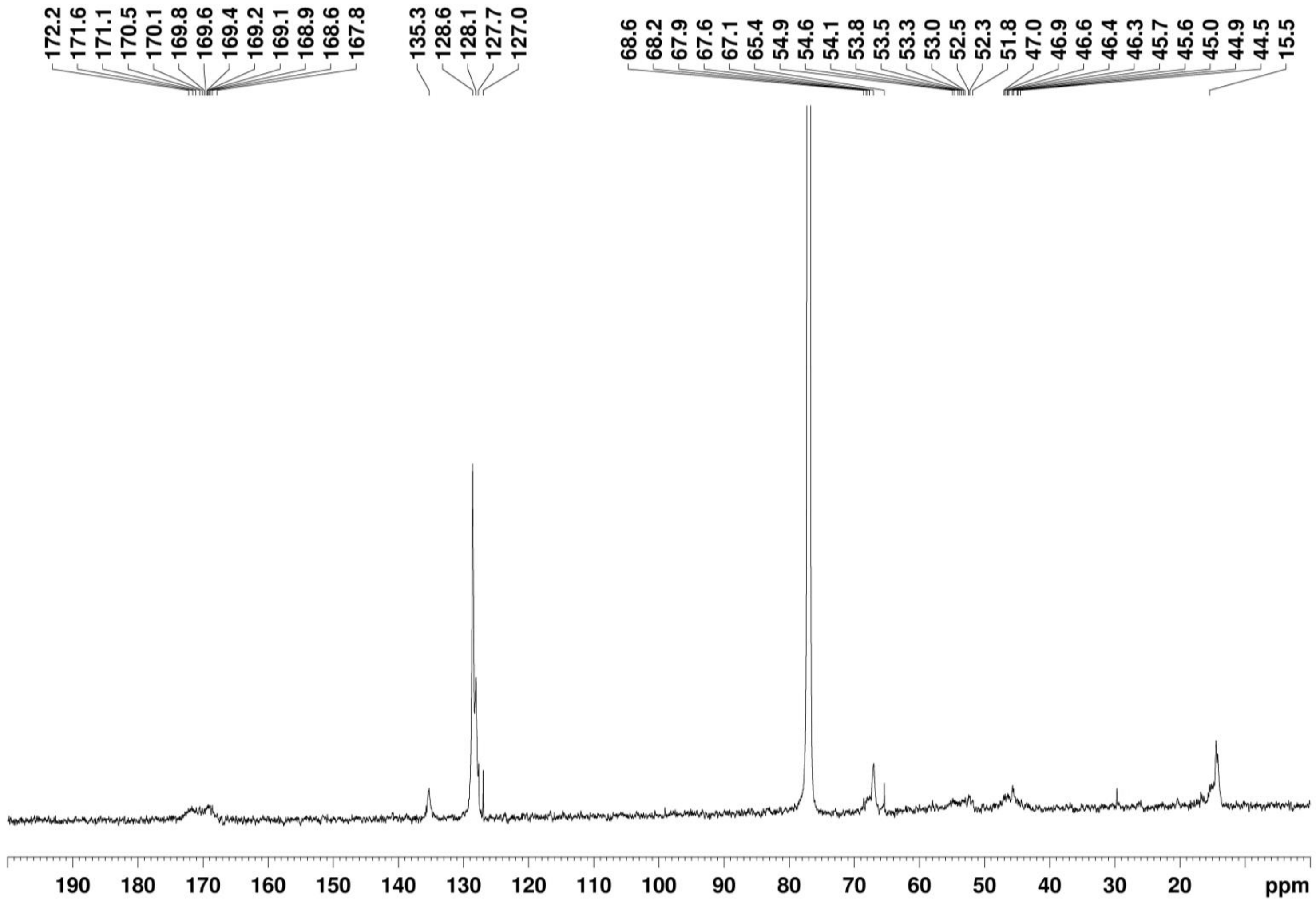

20: 13C{1H} NMR (150 MHz, $CDCl_3$)

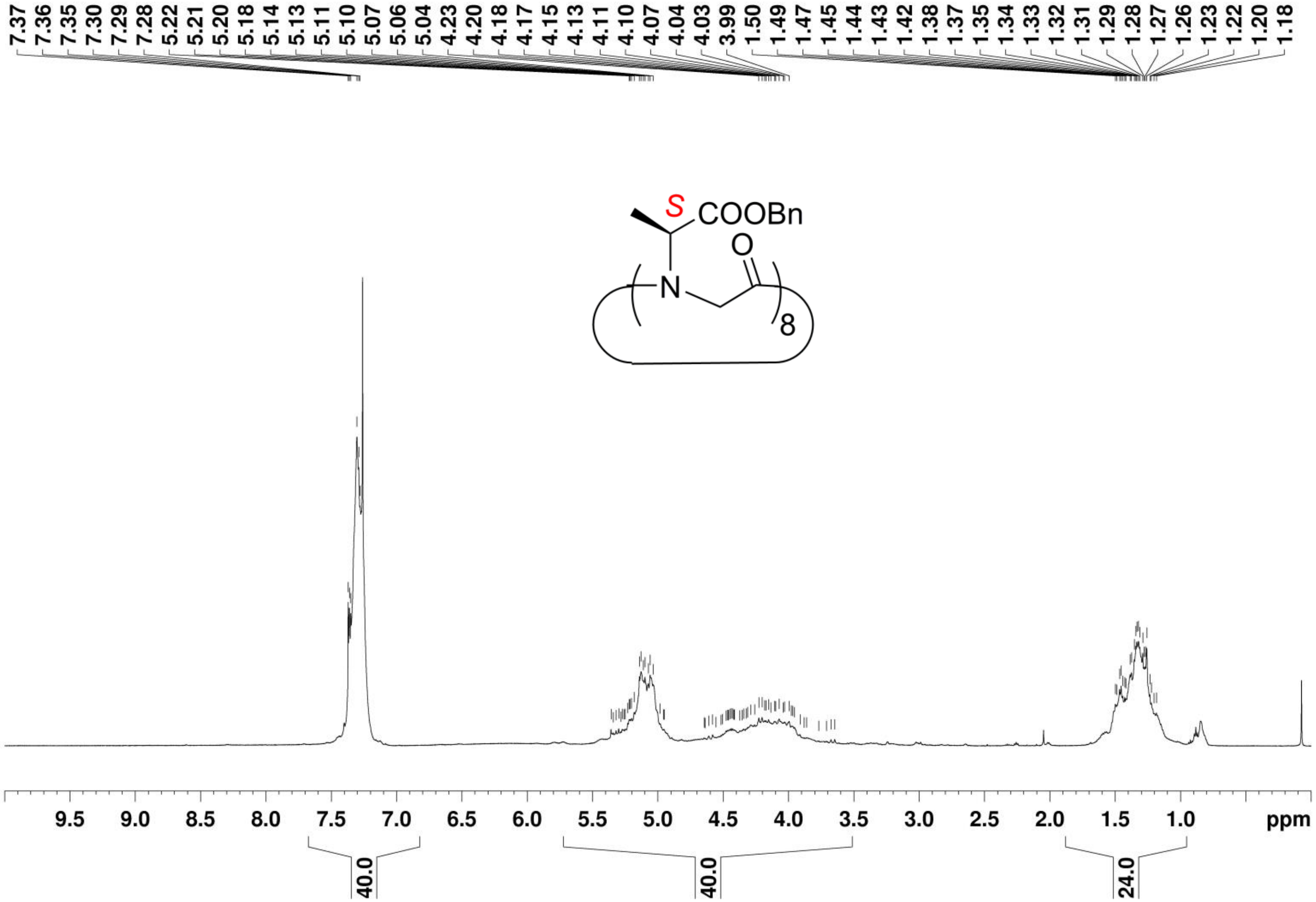

21: ^{1}H NMR (600 MHz, $CDCl_3$)

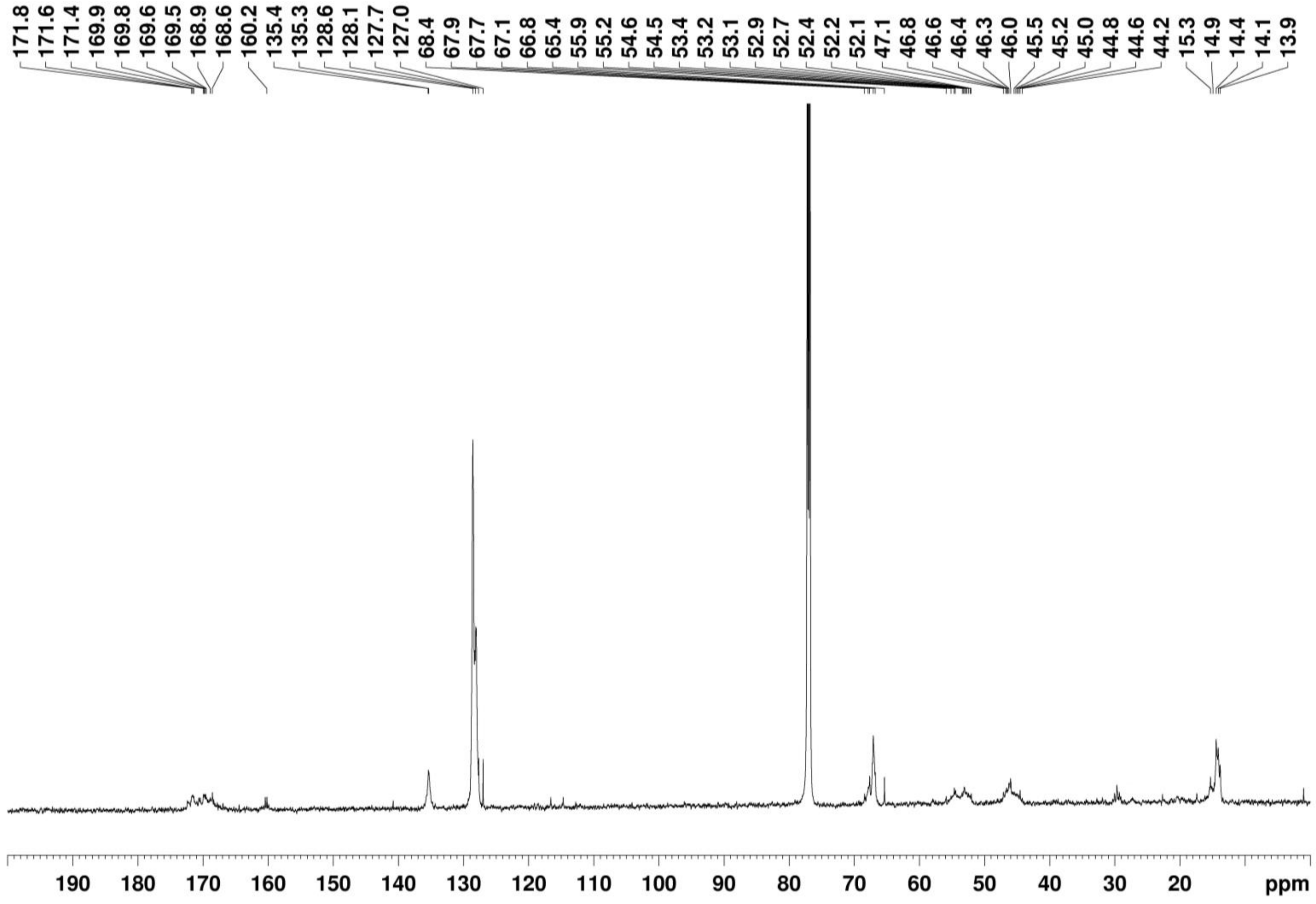

21: 13C{1H} NMR (150 MHz, $CDCl_3$)

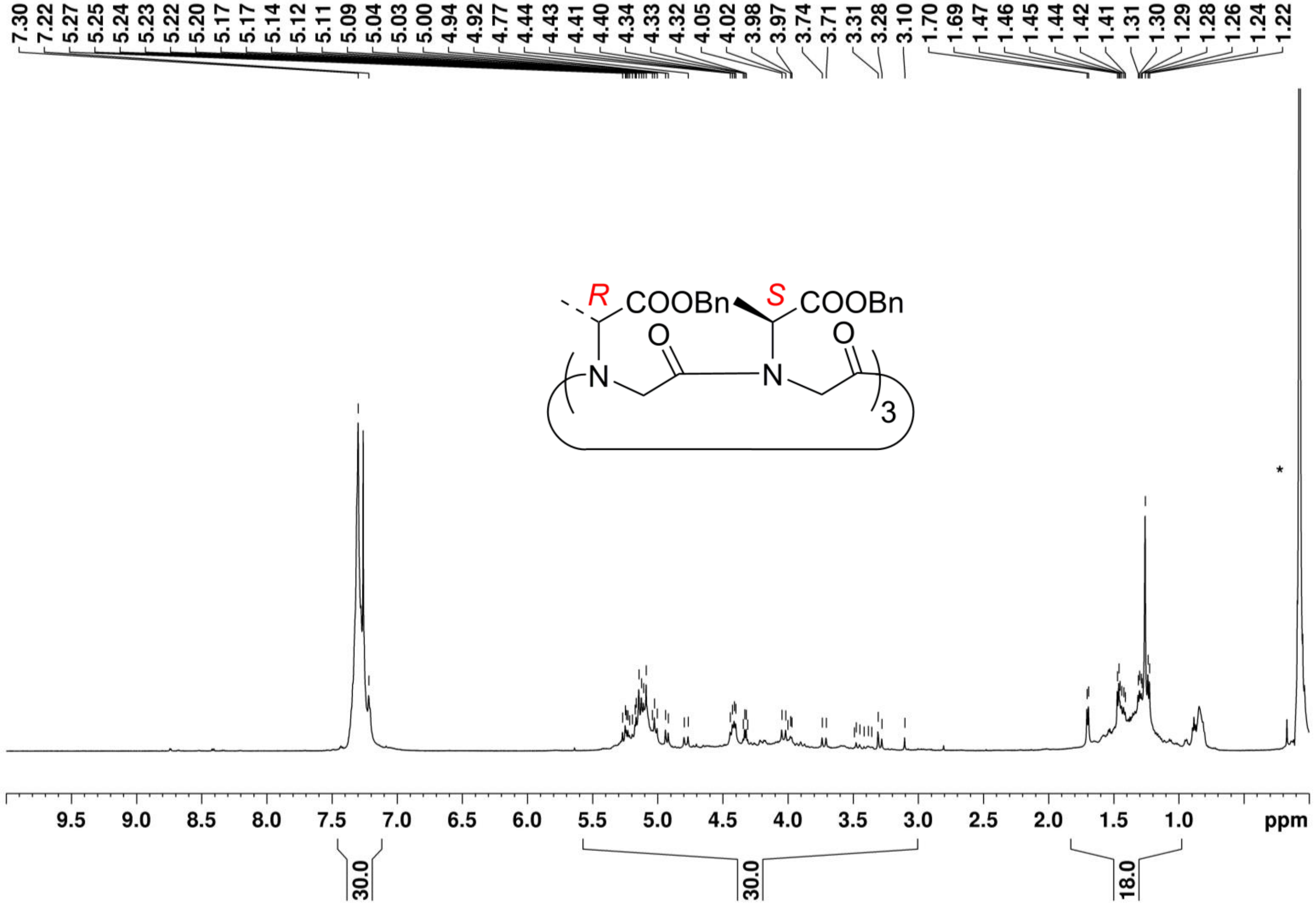

22: ^{1}H NMR (600 MHz, $CDCl_3$). Silicone grease impurity is marked with a black asterisk.

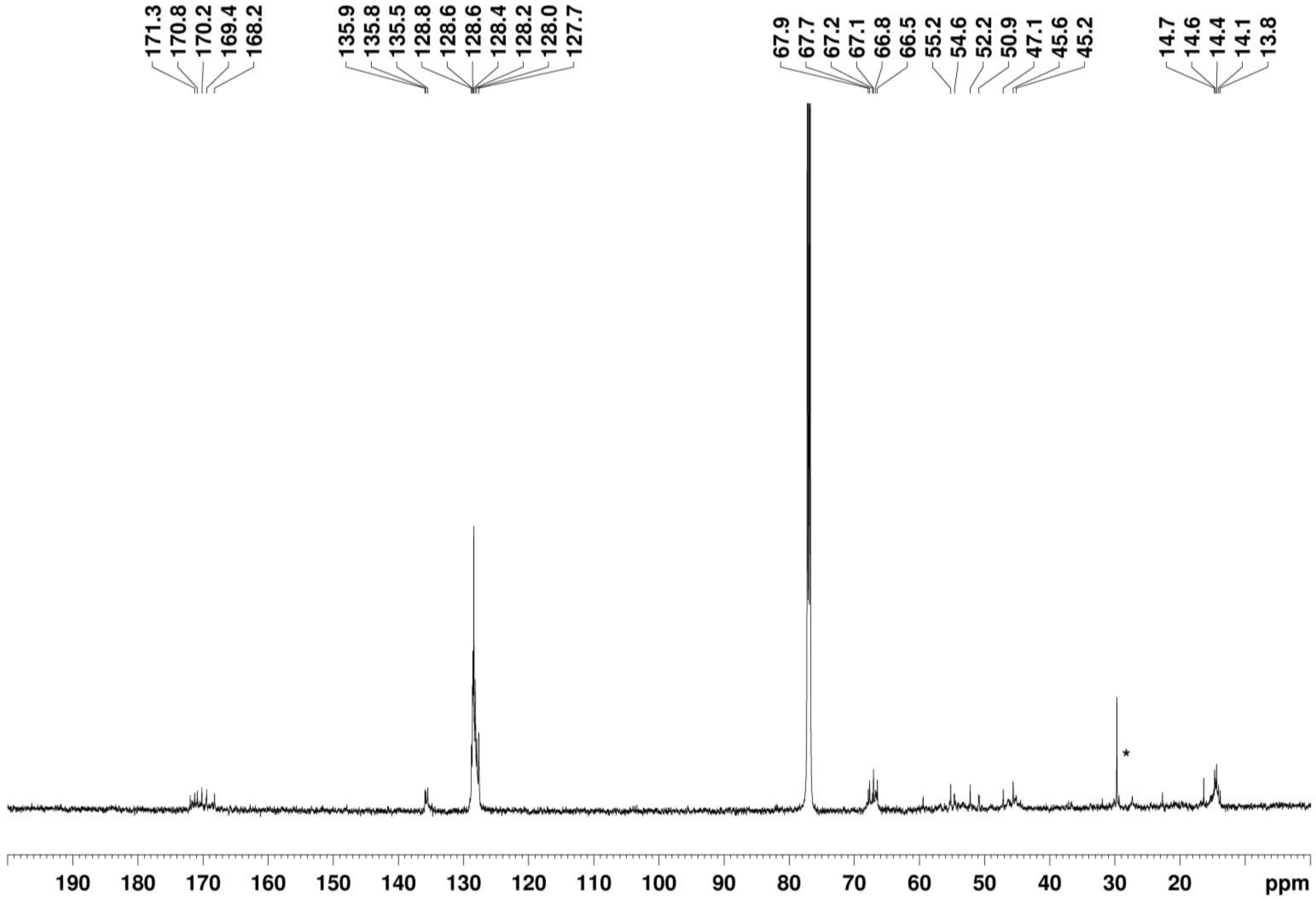

22: 13C{1H} NMR (150 MHz, $CDCl_3$). Grease impurity is marked with a black asterisk.

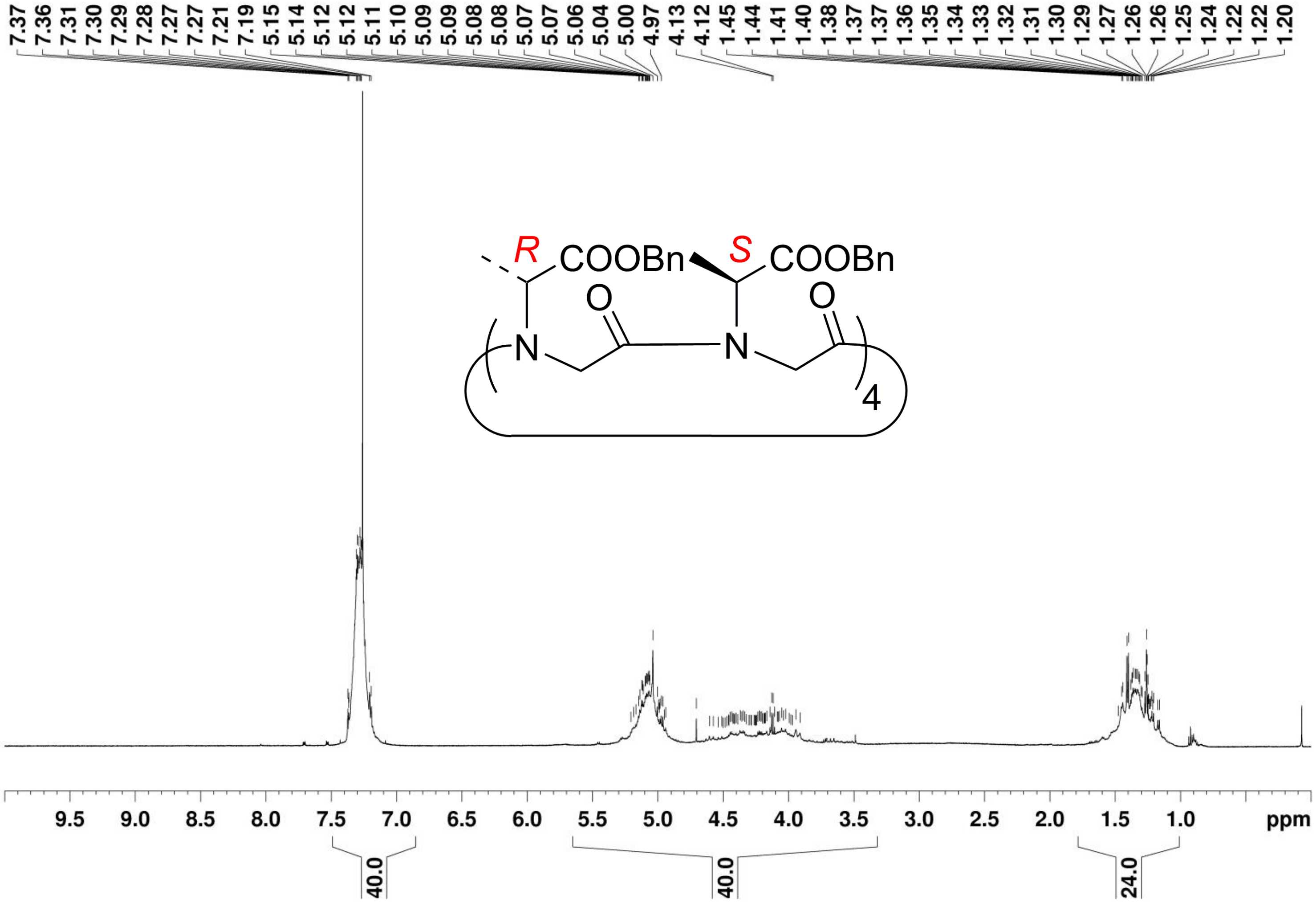

23: ^{1}H NMR (600 MHz, $CDCl_3$)

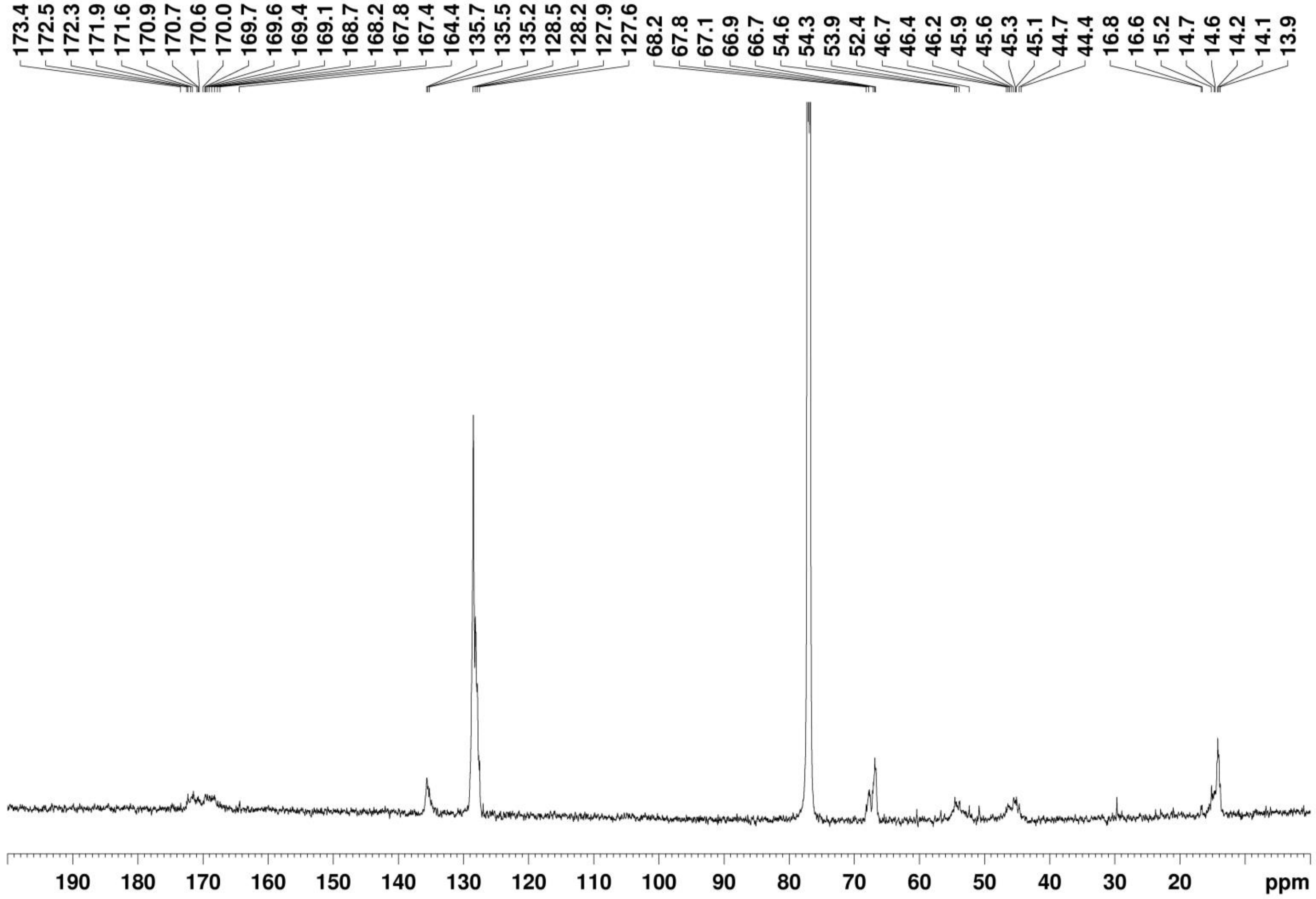

23: 13C{1H} NMR (150 MHz, $CDCl_3$)

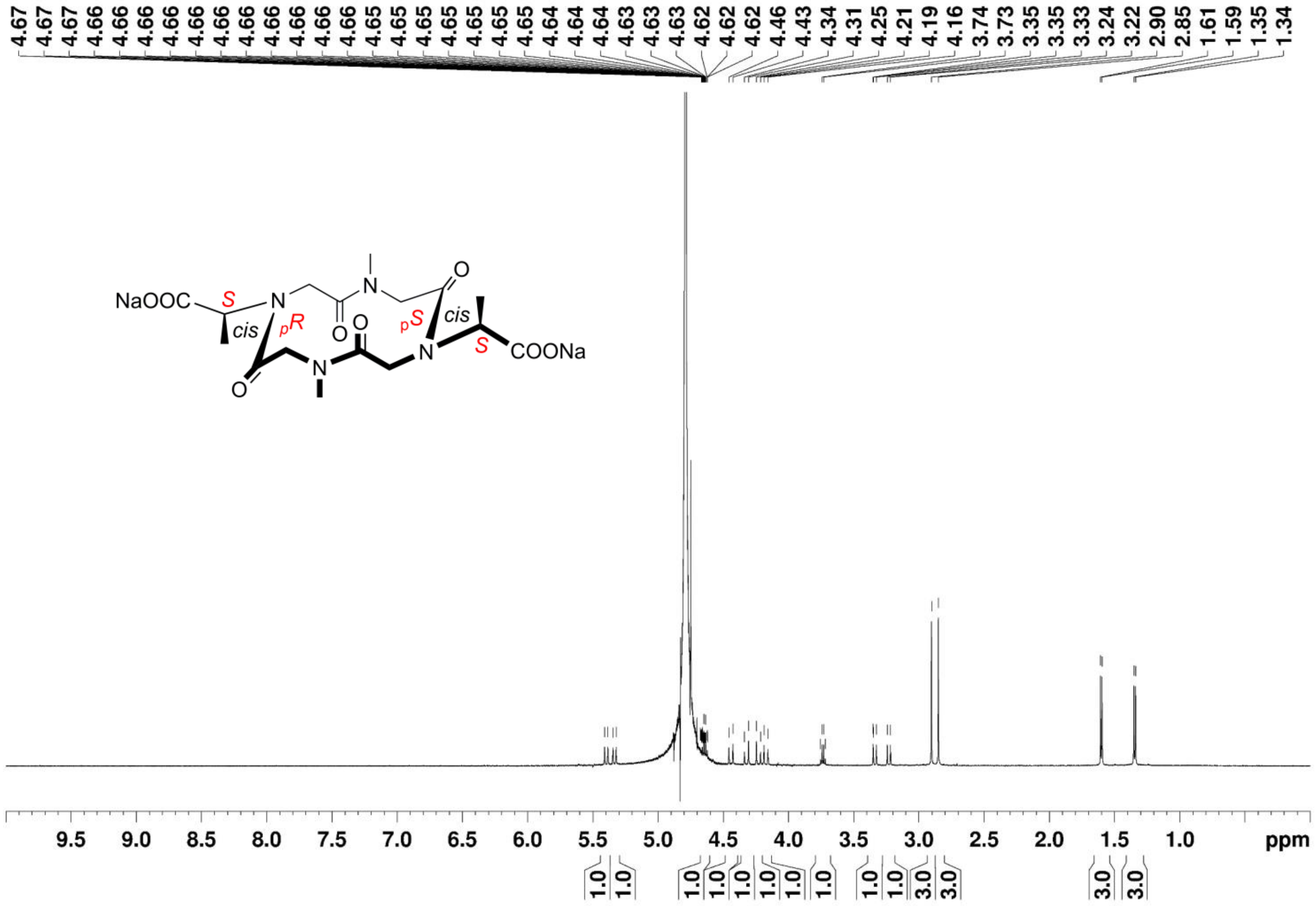

[**1**·Na_2]: ^{1}H NMR (600 MHz, pH 8 deuterated phosphate buffer)

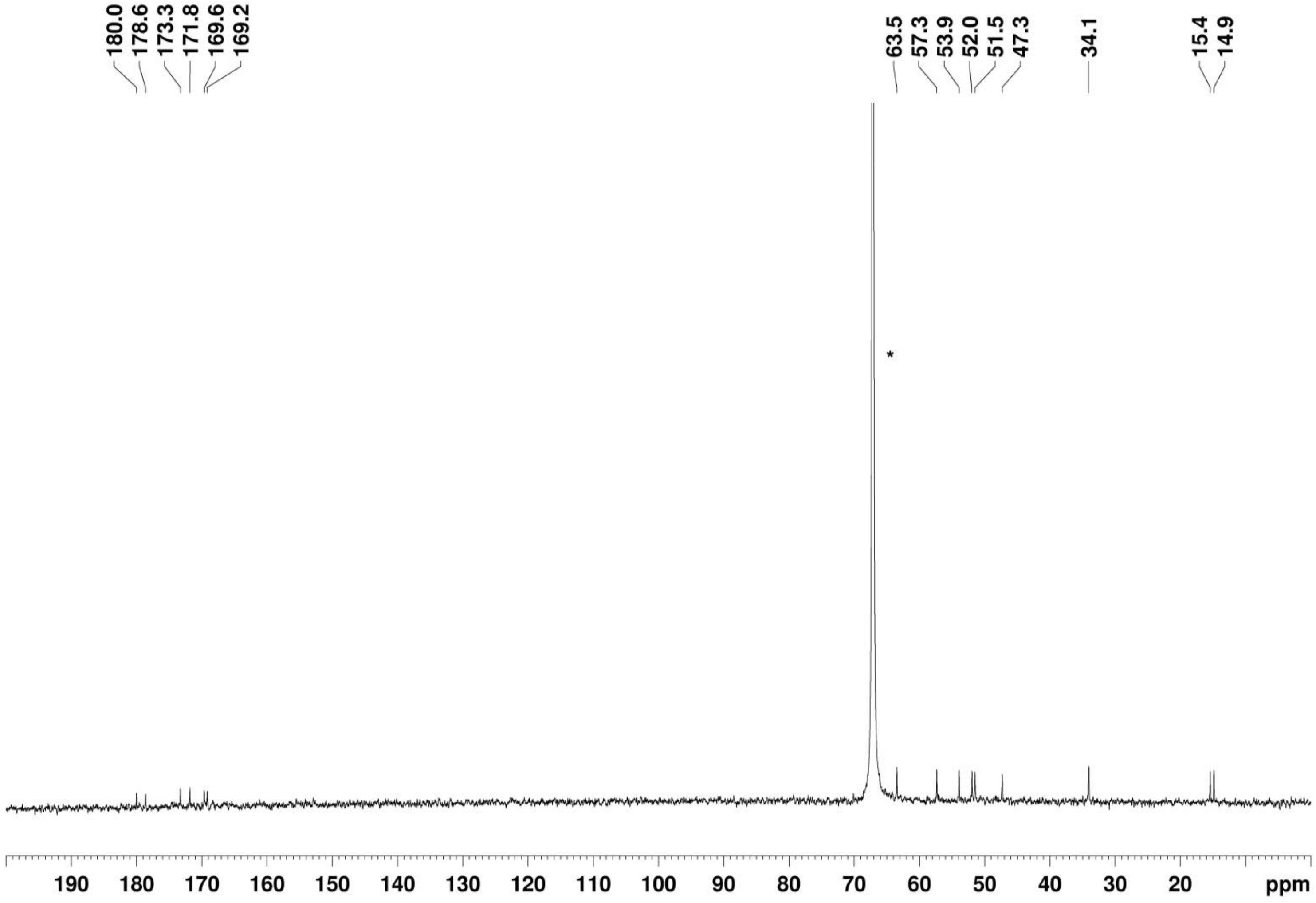

[**1**·Na_2]: 13C{1H} NMR (150 MHz, pH 8 deuterated phosphate buffer). 1,4-dioxane signal (internal standard) is marked with a black asterisk.

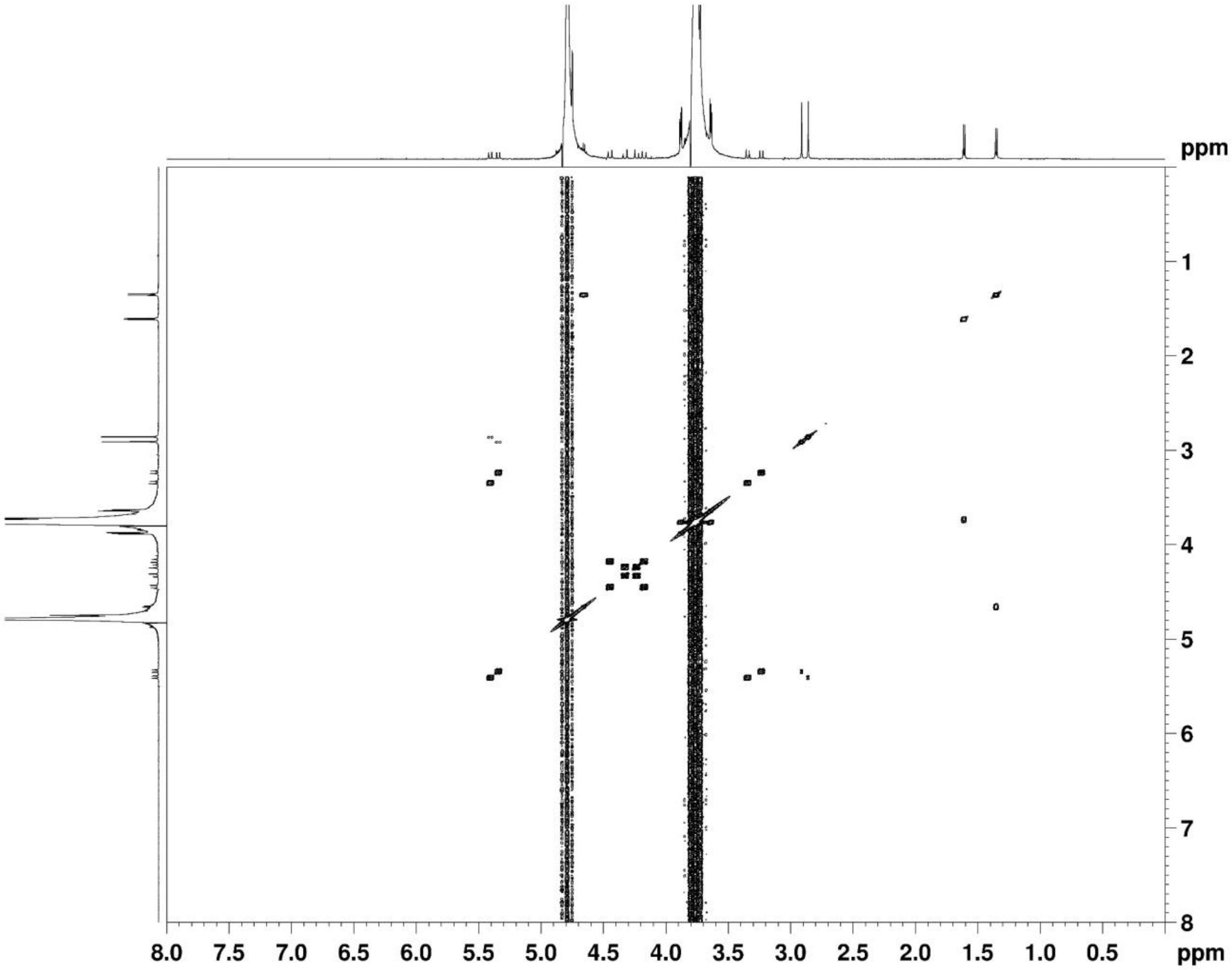

[**1**·Na_2]: COSY SPECTRUM (600 MHz, pH 8 deuterated phosphate buffer). The sample contains 1,4-dioxane.

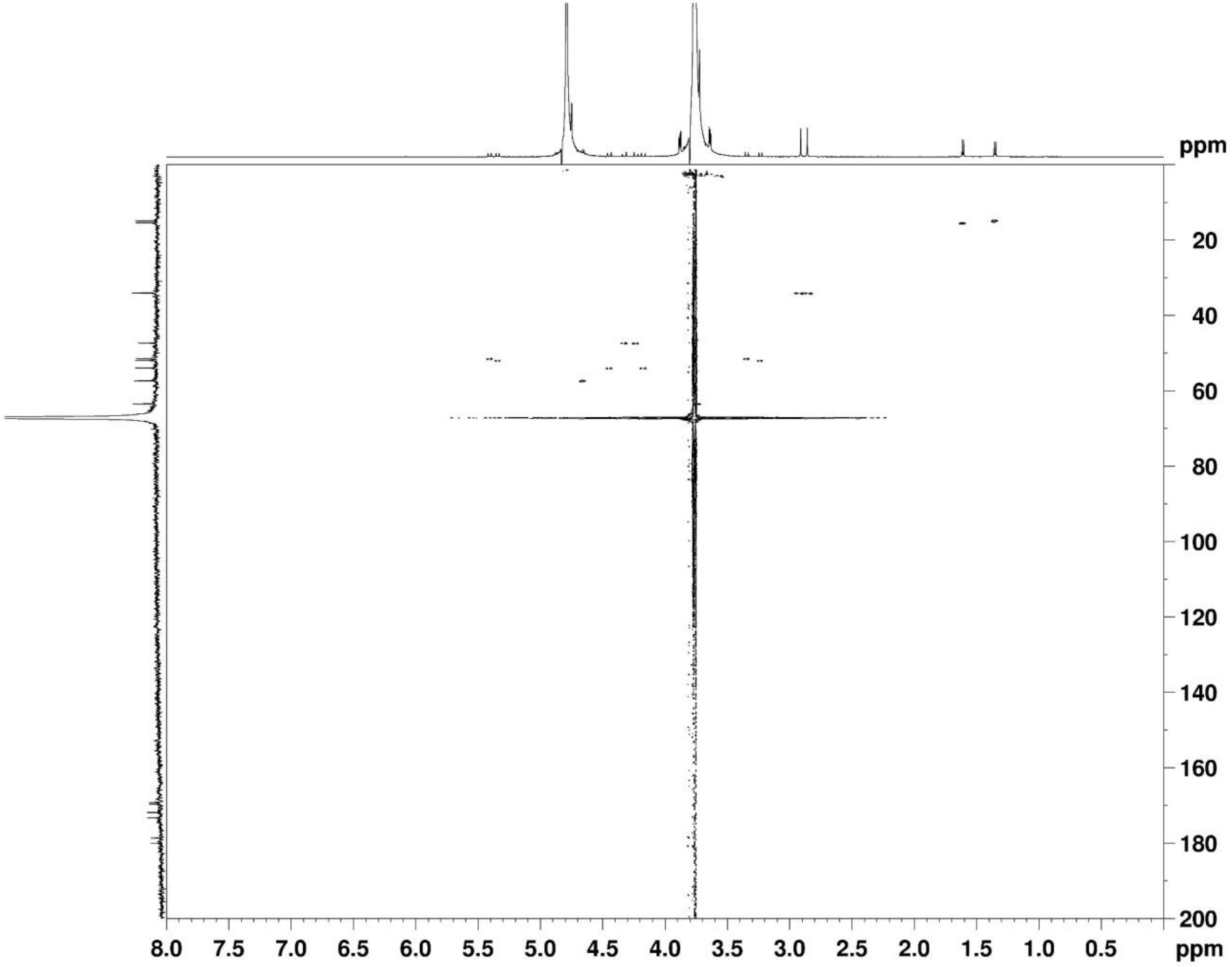

[**1**·Na_2]: HSQC SPECTRUM (600 MHz, pH 8 deuterated phosphate buffer). The sample contains 1,4-dioxane.

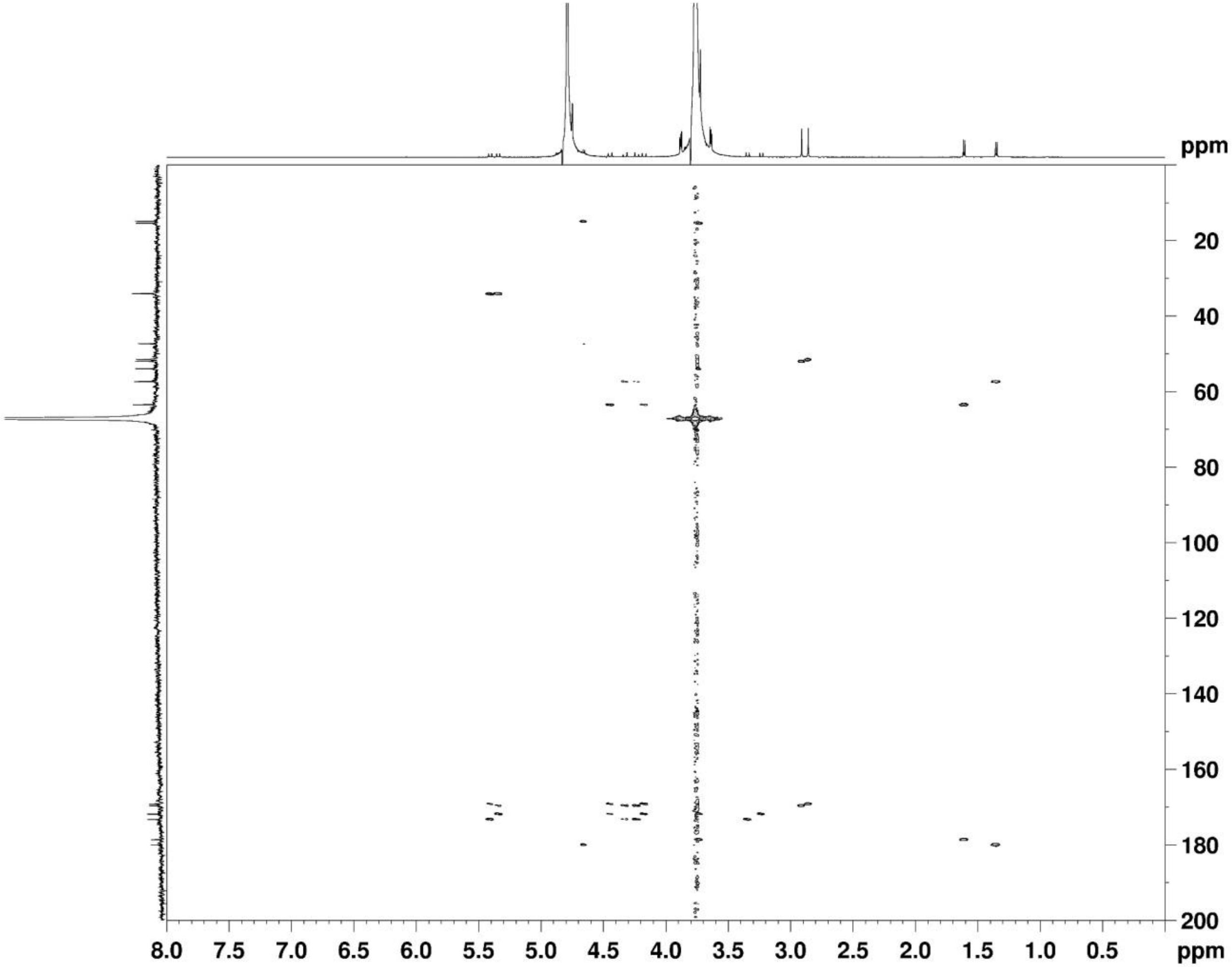

[**1**·Na_2]: HMBC SPECTRUM (600 MHz, pH 8 deuterated phosphate buffer). The sample contains 1,4-dioxane.

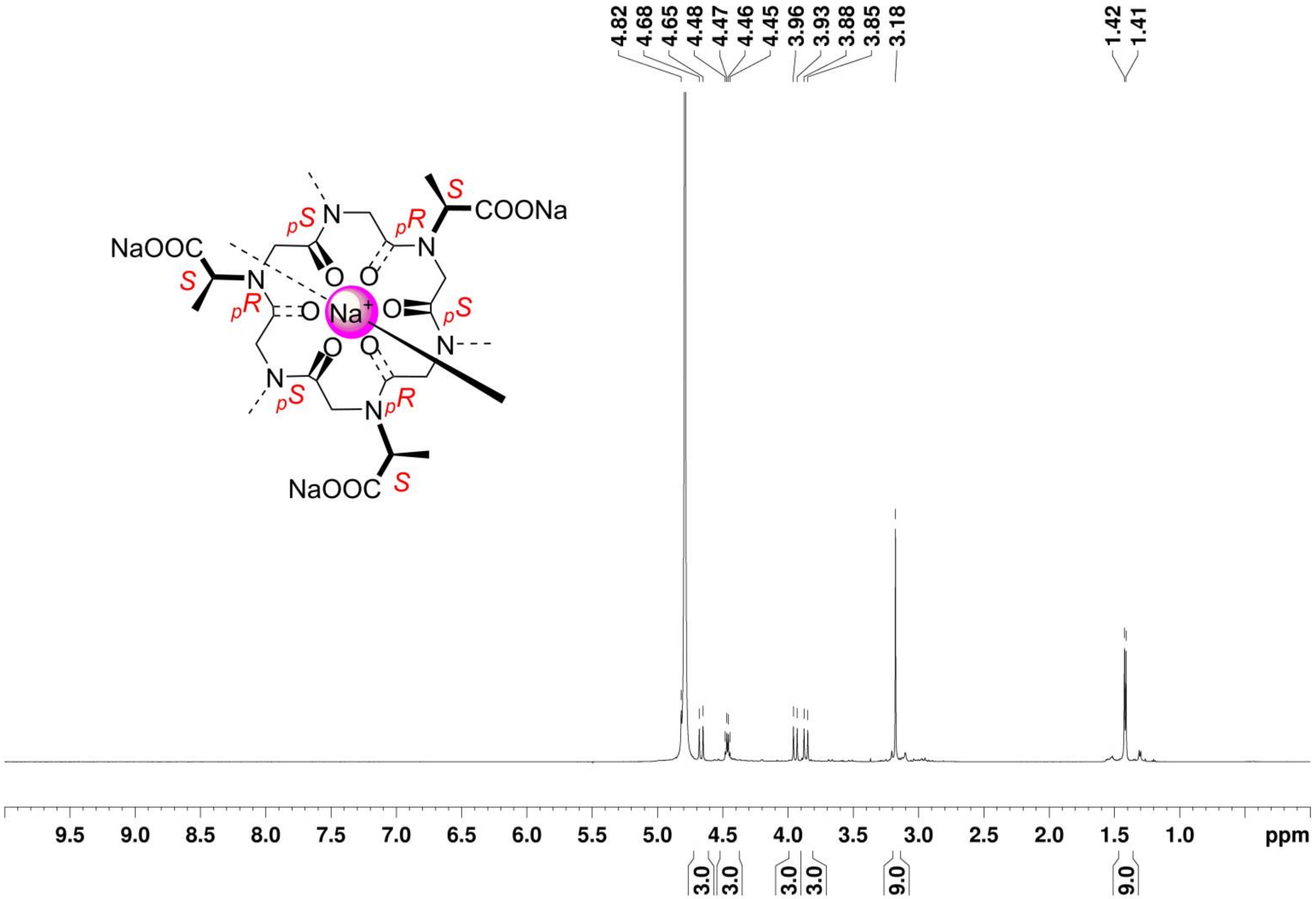

[**2**·4Na]$^{+}$: ^{1}H NMR (600 MHz, pH 8 deuterated phosphate buffer)

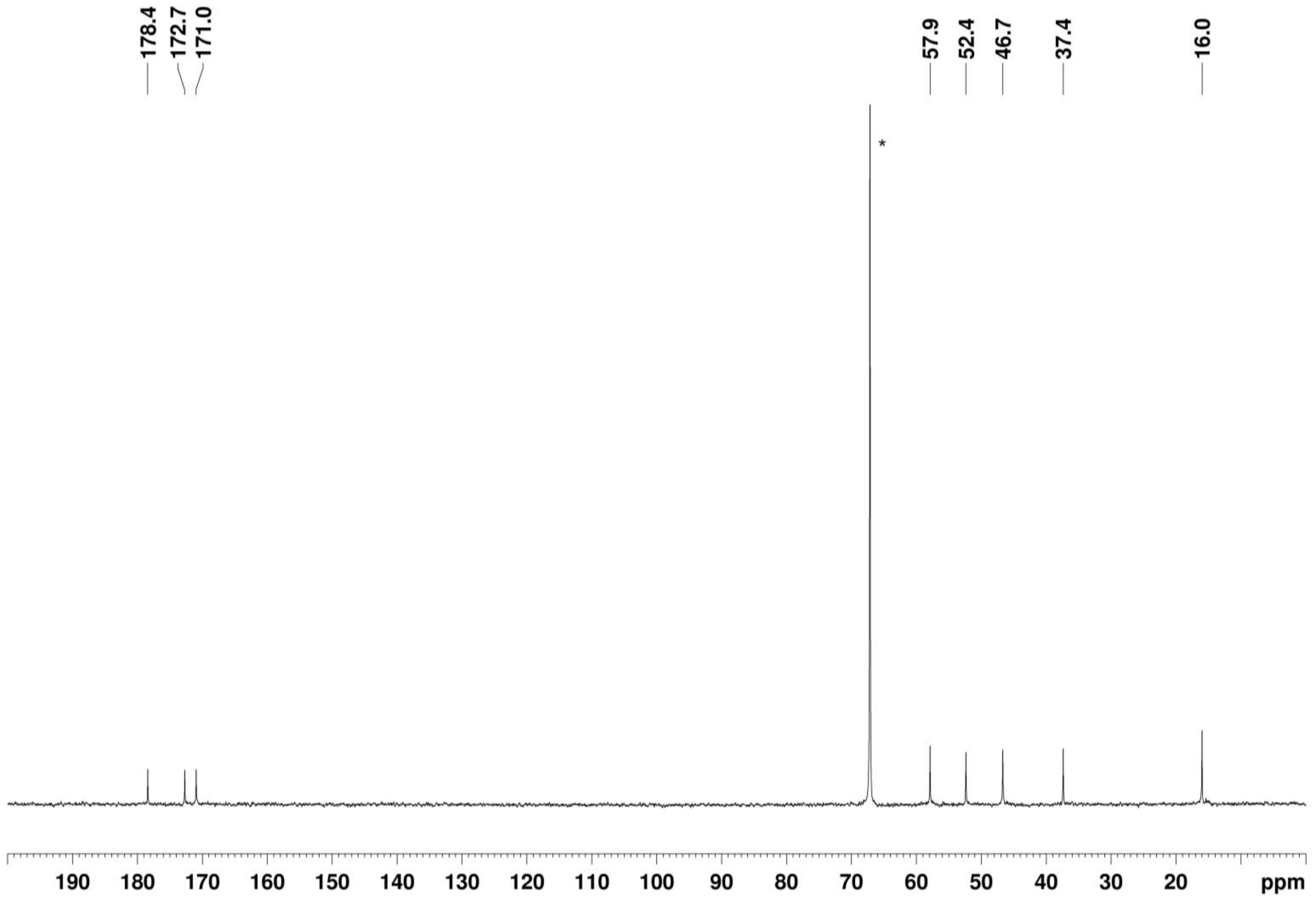

[**2**·4Na]$^{+}$: 13C{1H} NMR (150 MHz, pH 8 deuterated phosphate buffer). 1,4-dioxane signal is marked with a black asterisk.

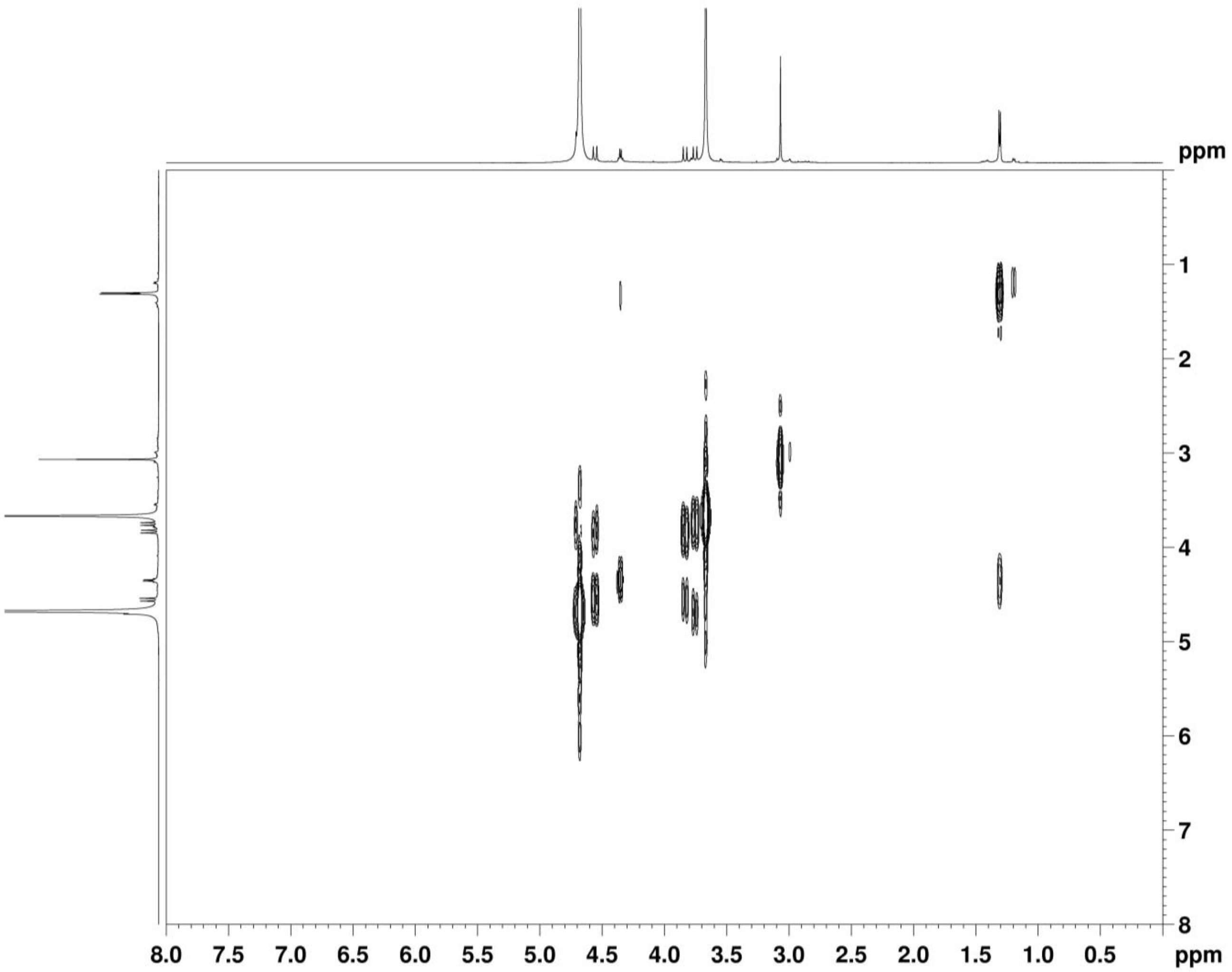

[**2**·4Na]$^+$: COSY SPECTRUM (600 MHz, pH 8 deuterated phosphate buffer). The sample contains 1,4-dioxane.

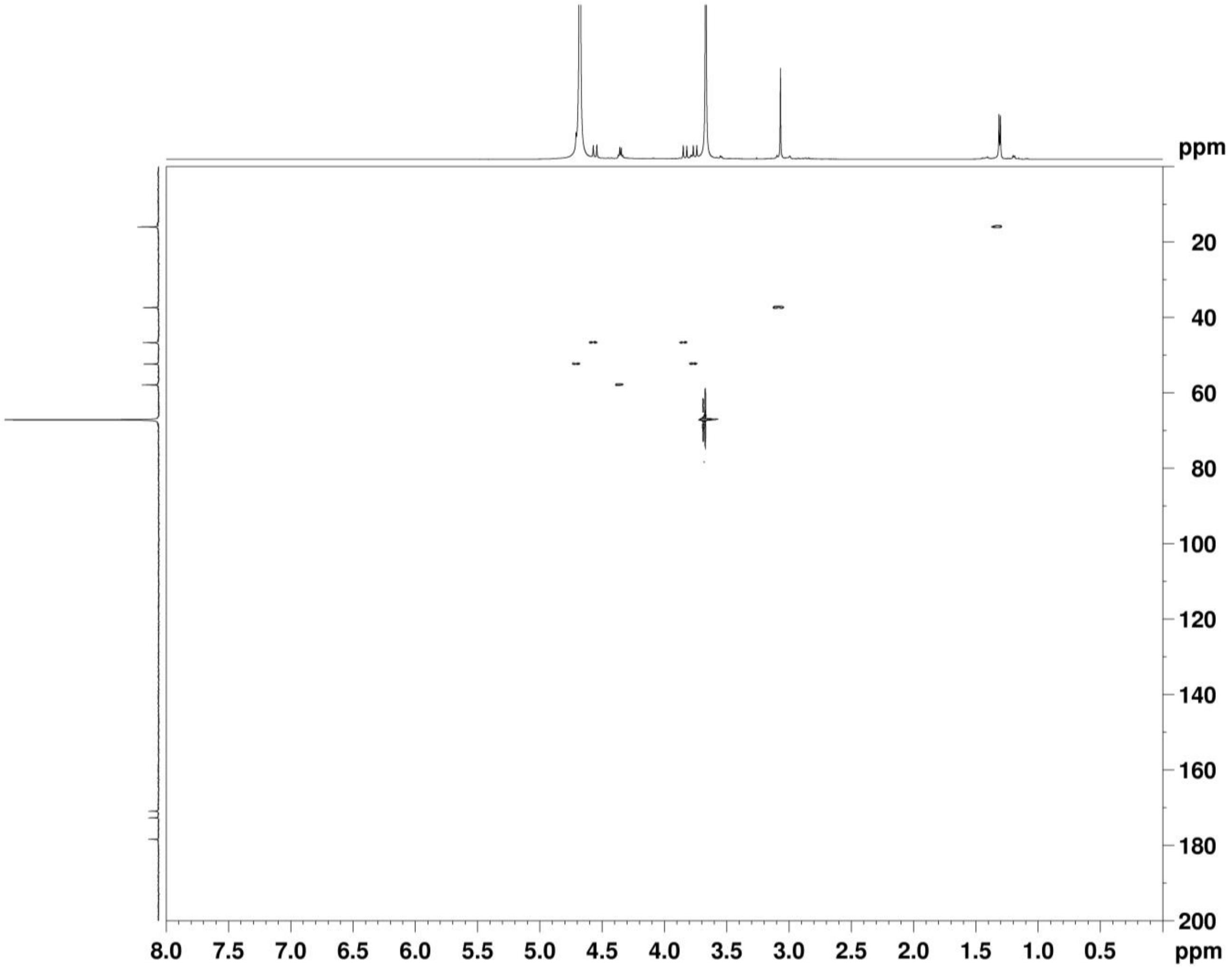

[**2**·4Na]$^+$: HSQC SPECTRUM (600 MHz, pH 8 deuterated phosphate buffer). The sample contains 1,4-dioxane.

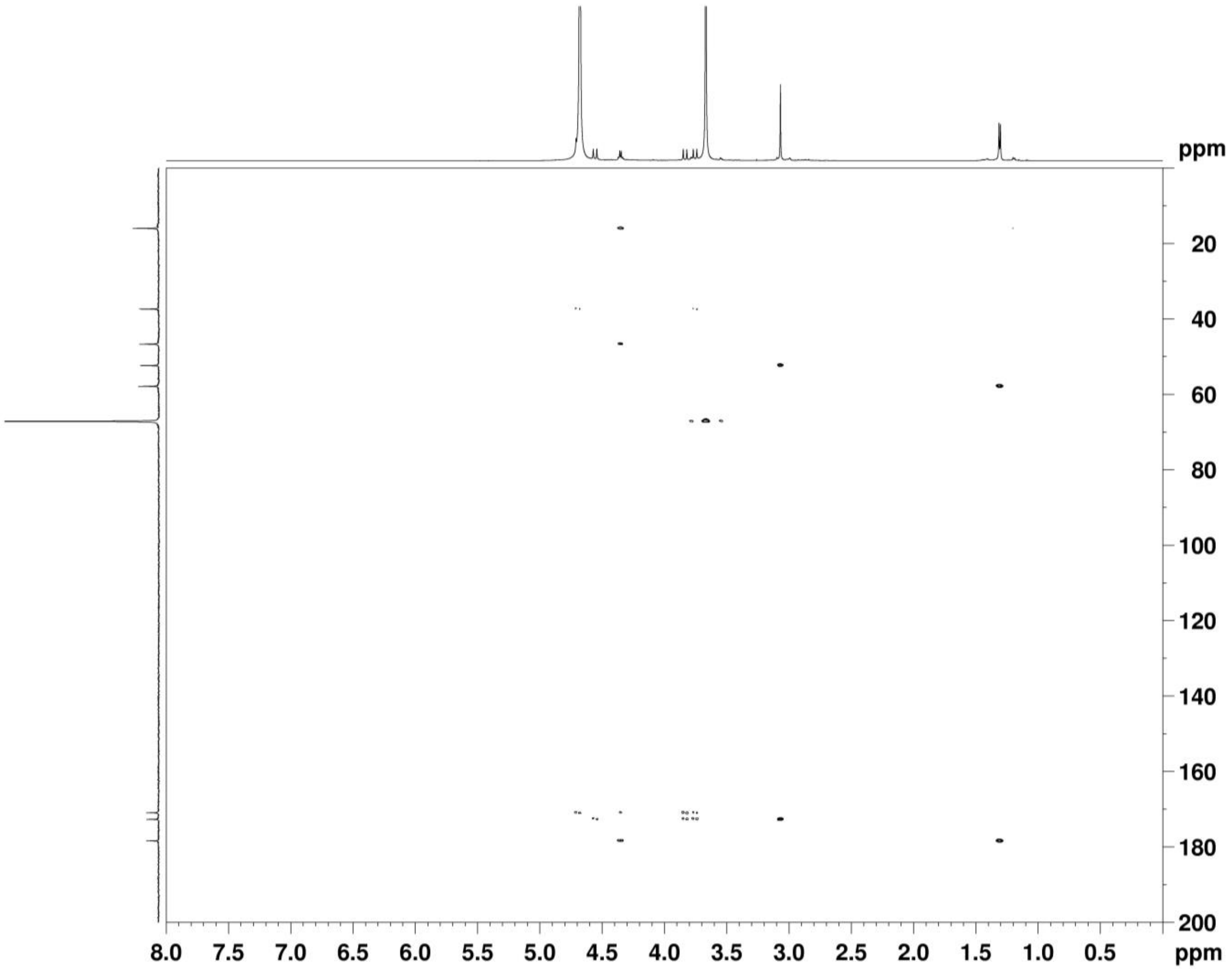

[**2**·4Na]$^{+}$: HMBC SPECTRUM (600 MHz, pH 8 deuterated phosphate buffer). The sample contains 1,4-dioxane.

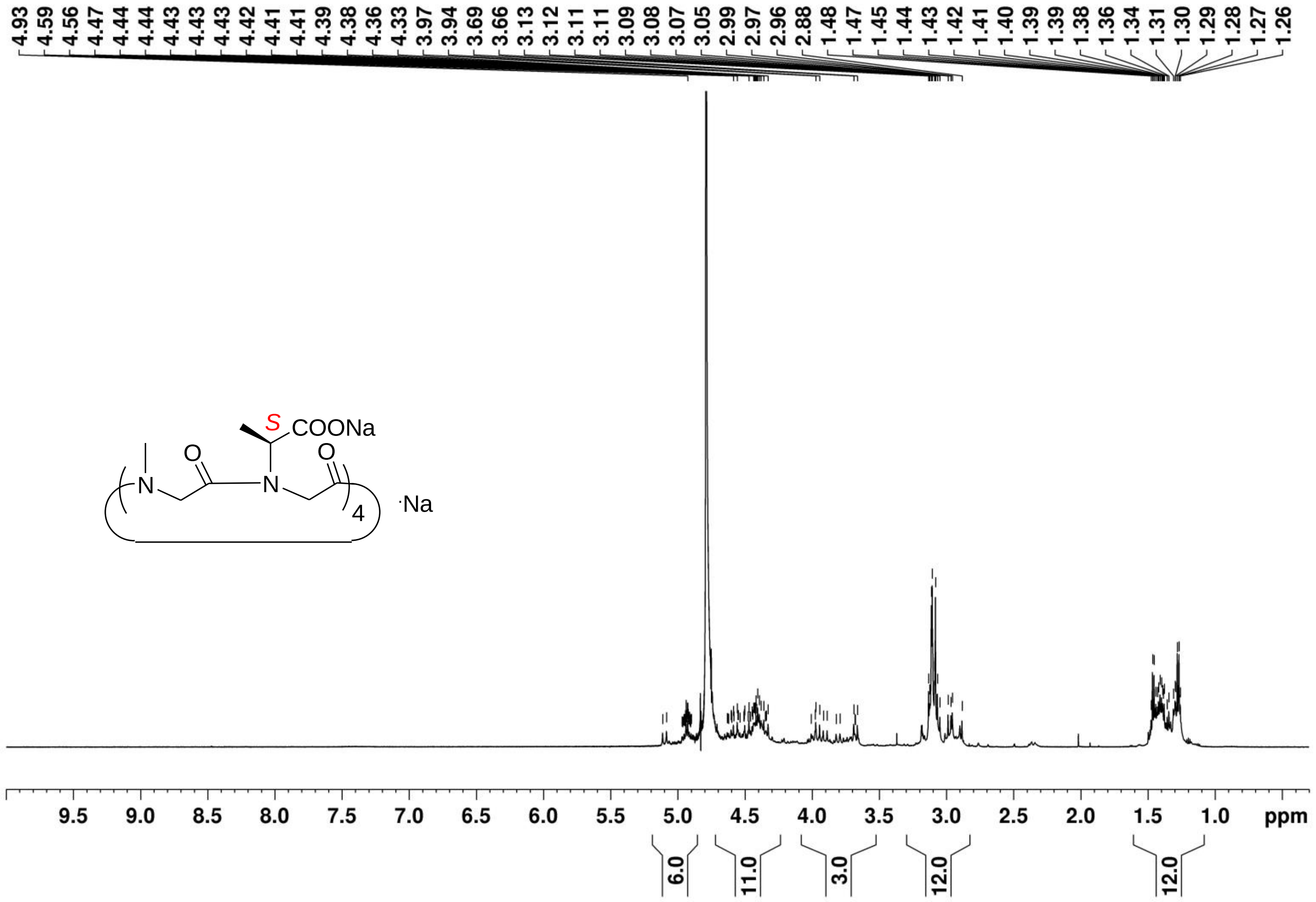

[**3**·5Na]$^{+}$: ^{1}H NMR (600 MHz, pH 8 deuterated phosphate buffer)

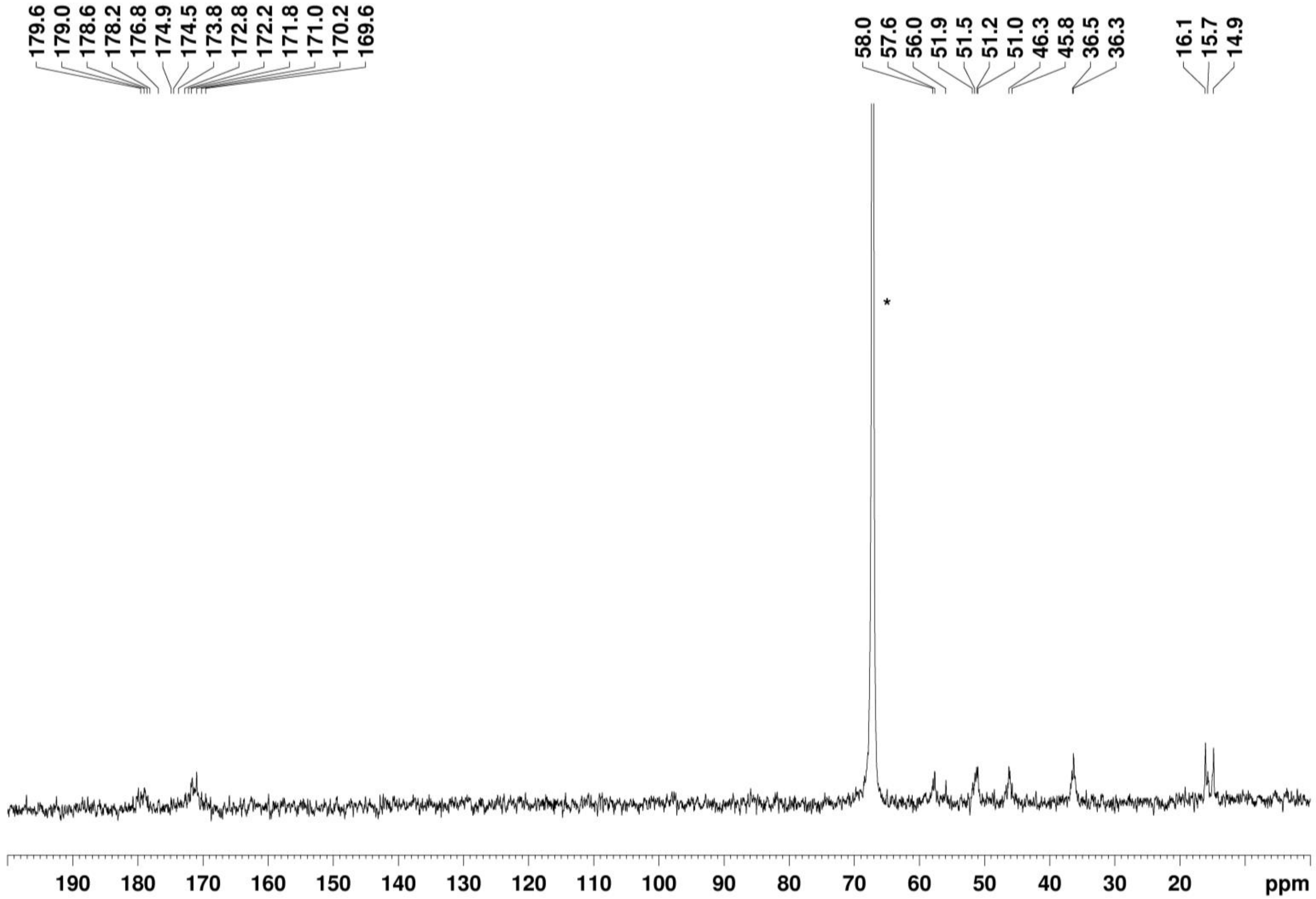

[**3**·5Na]+: 13C{1H} NMR (150 MHz, pH 8 deuterated phosphate buffer). 1,4-dioxane signal is marked with a black asterisk.

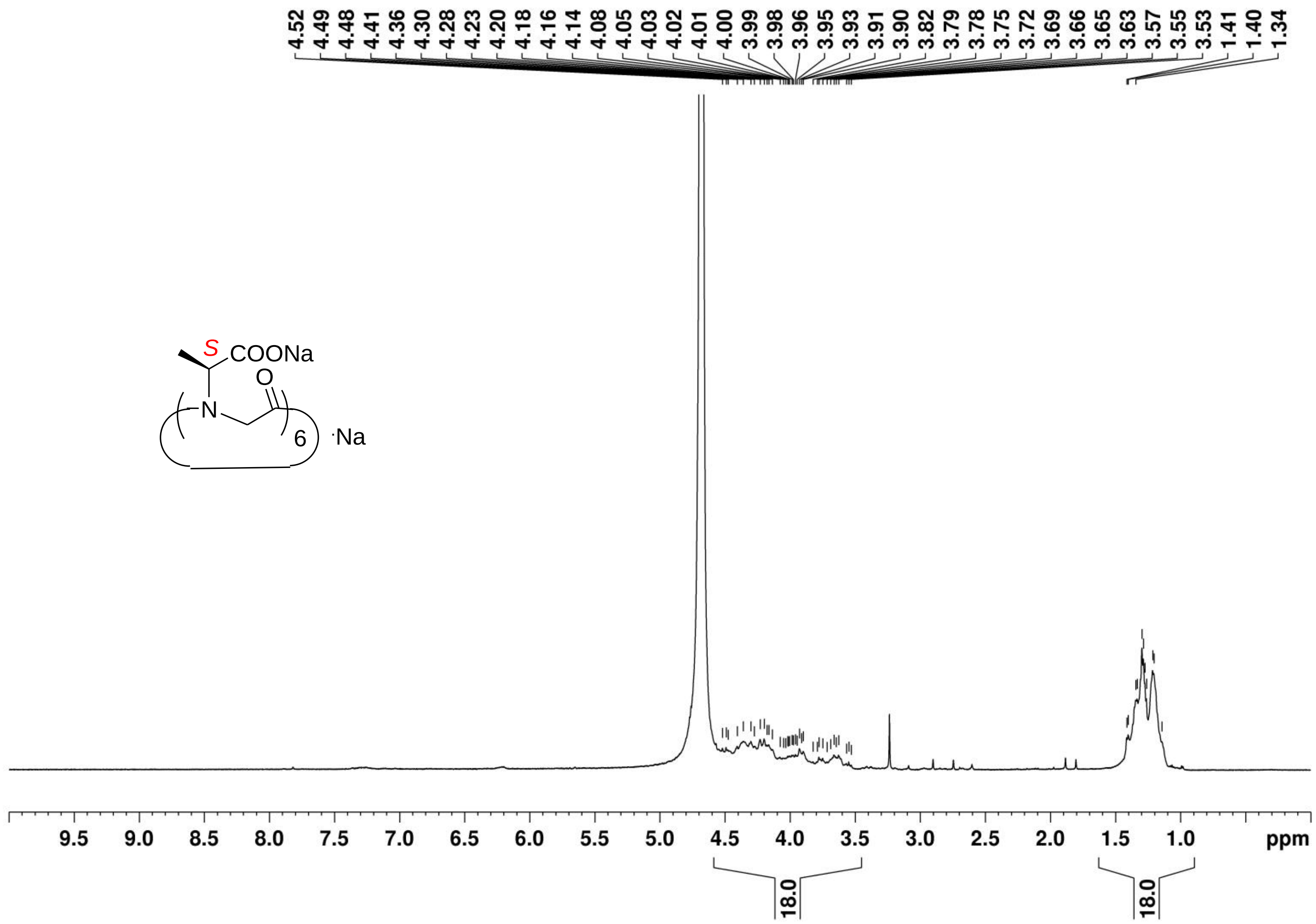

[**4**·7Na]$^+$: ^{1}H NMR (600 MHz, pH 8 deuterated phosphate buffer)

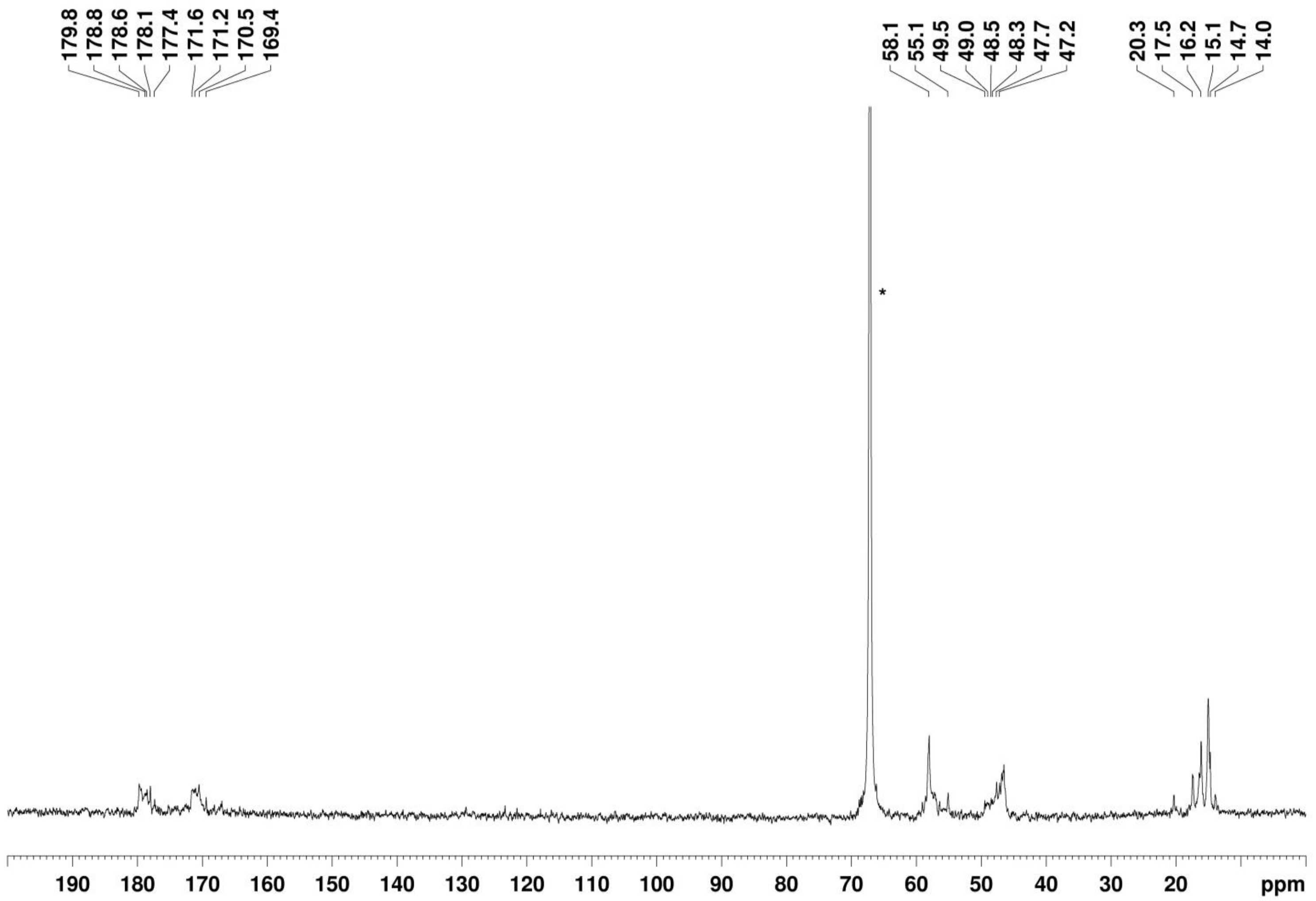

[**4**·7Na]$^{+}$: 13C{1H} NMR (150 MHz, pH 8 deuterated phosphate buffer). 1,4-dioxane signal is marked with a black asterisk.

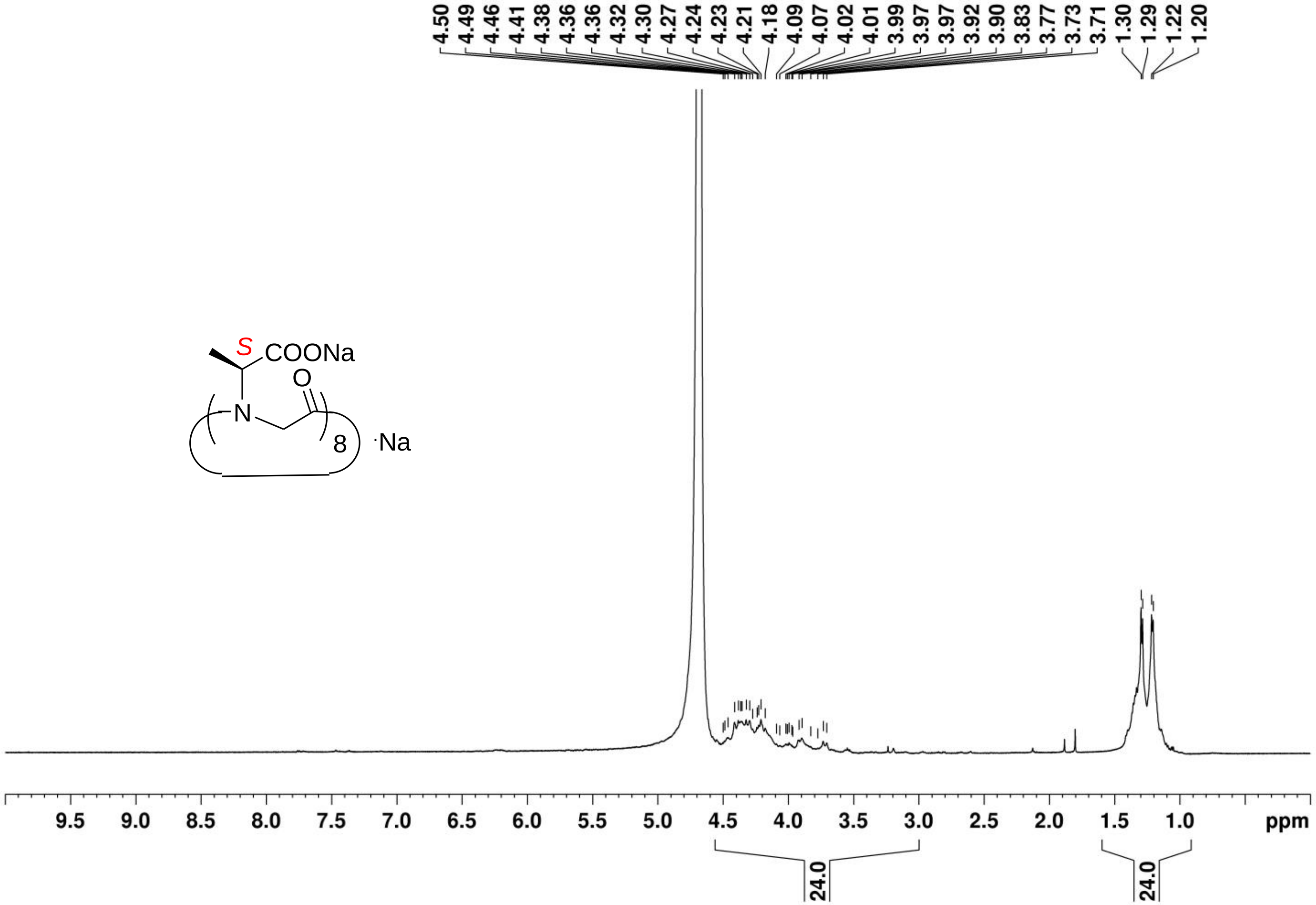

[**5**·9Na]$^{+}$: ^{1}H NMR (600 MHz, pH 8 deuterated phosphate buffer)

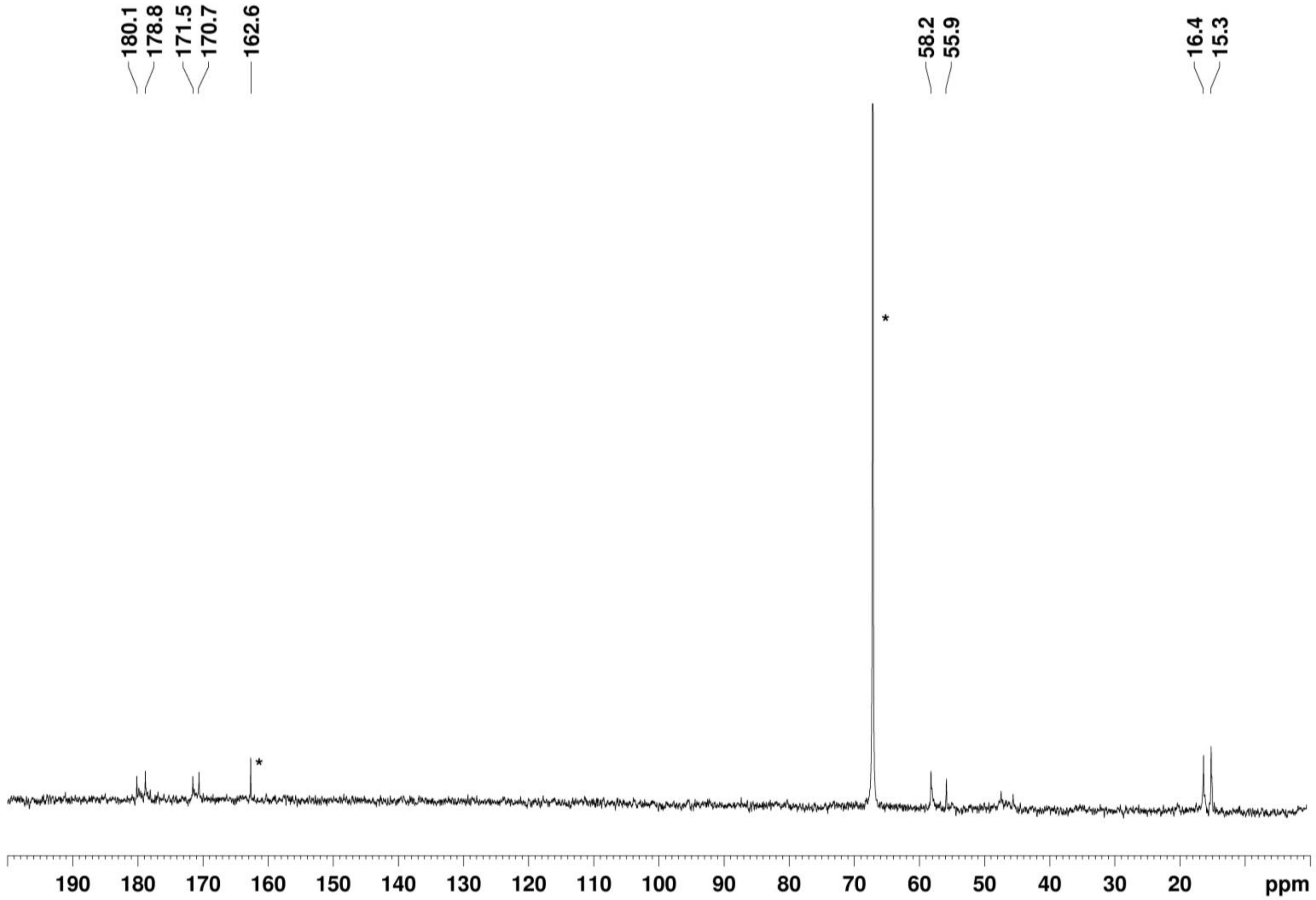

[**5**·9Na]$^+$: 13C{1H} NMR (150 MHz, pH 8 deuterated phosphate buffer). 1,4-dioxane (67.15 ppm) and HCO_3^- (162.6 ppm) impurities signals are marked with a black asterisk.

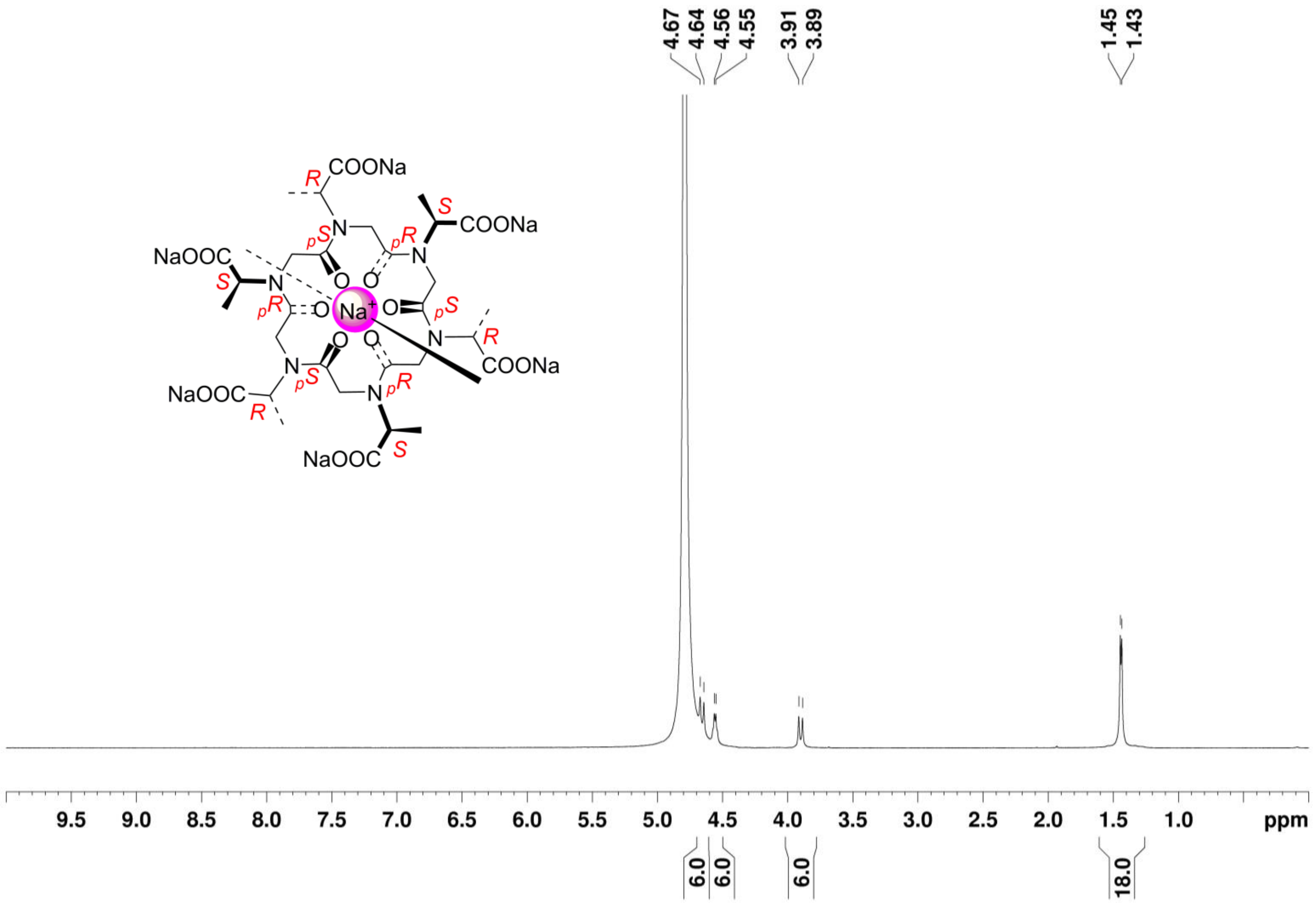

[**6**·7Na]$^{+}$: ^{1}H NMR (600 MHz, pH 8 deuterated phosphate buffer)

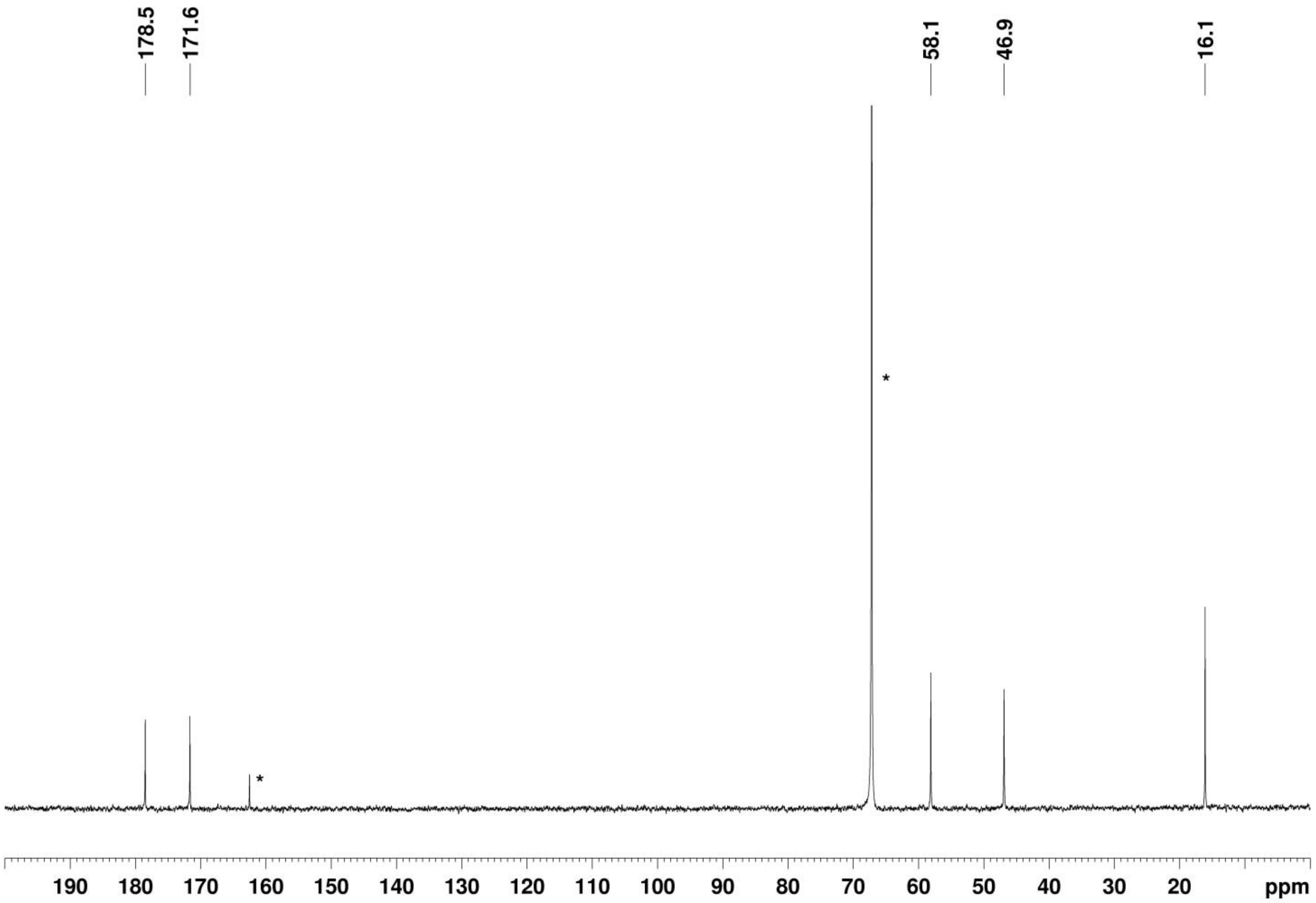

[**6**·7Na]$^+$: 13C{1H} NMR (150 MHz, pH 8 deuterated phosphate buffer). 1,4-dioxane (67.15 ppm) and HCO_3^- (162.6 ppm) impurities signals are marked with a black asterisk.

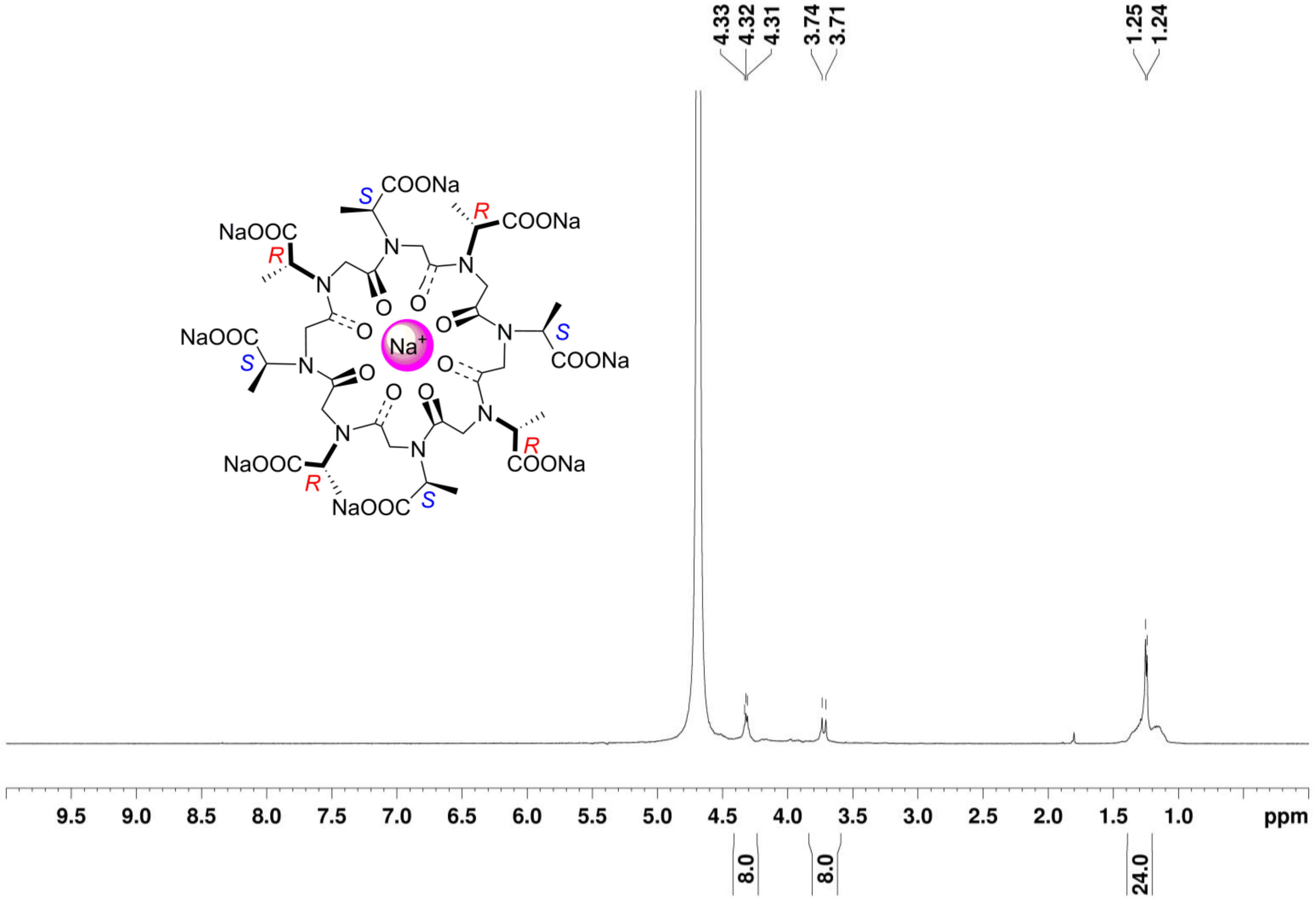

[**7**·9Na]$^+$: ^{1}H NMR (600 MHz, pH 8 deuterated phosphate buffer)

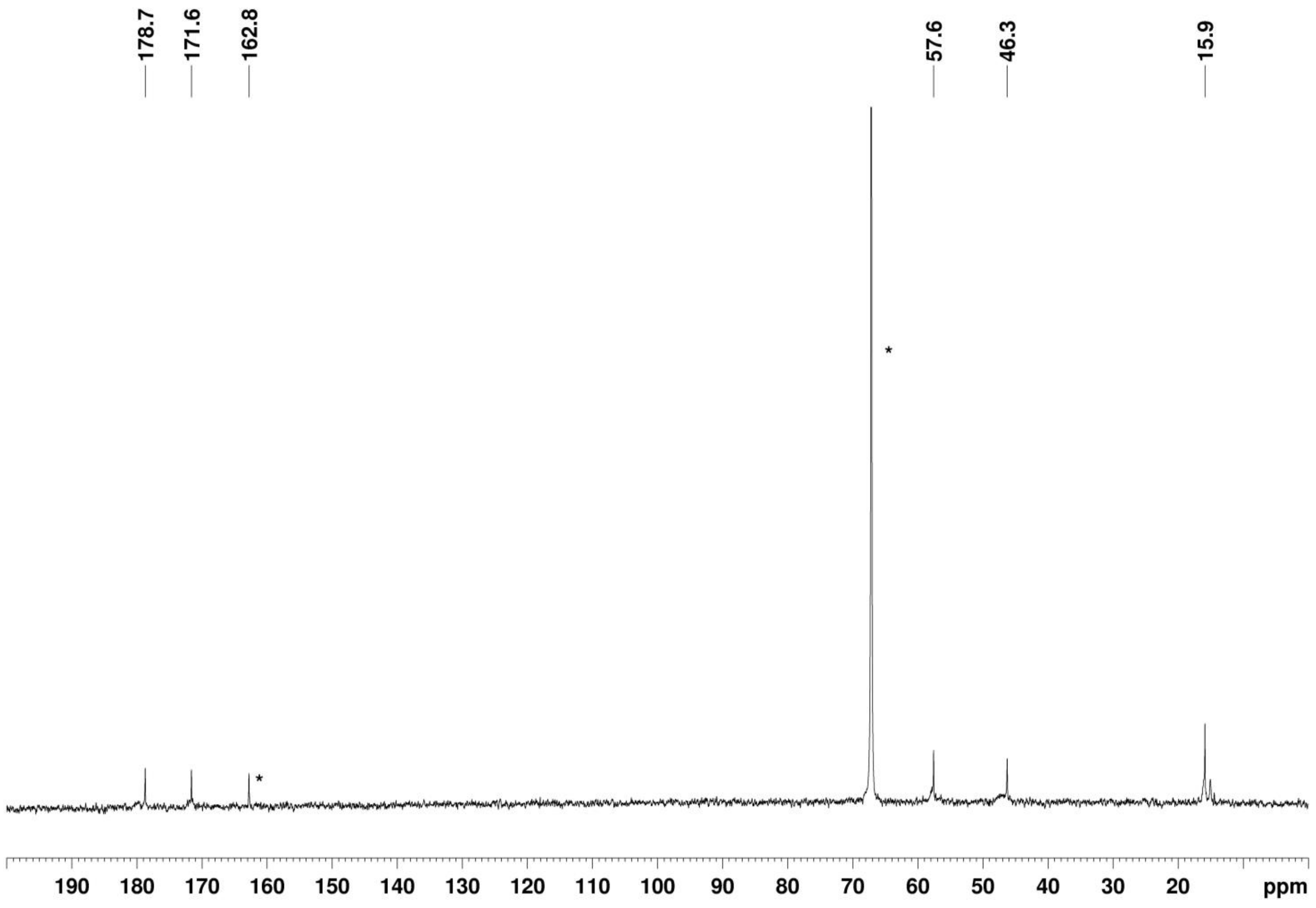

[**7**·9Na]$^+$: 13C{1H} NMR (150 MHz, pH 8 deuterated phosphate buffer). 1,4-dioxane (67.15 ppm) and HCO_3^- (162.6 ppm) impurities signals are marked with a black asterisk.

3.0 HPLC Chromatograms

3.1 HPLC chromatograms of linear peptoids **8-16** as crude mixtures (Figures S1-S9). Partial insolubility of the analytes hampered quantitative evaluation of the impurities.

Conditions: 5 → 100% A in 30 min (A, 0.1% TFA in acetonitrile, B, 0.1% TFA in water); flow: 1 mL min^{-1}, 220 nm.

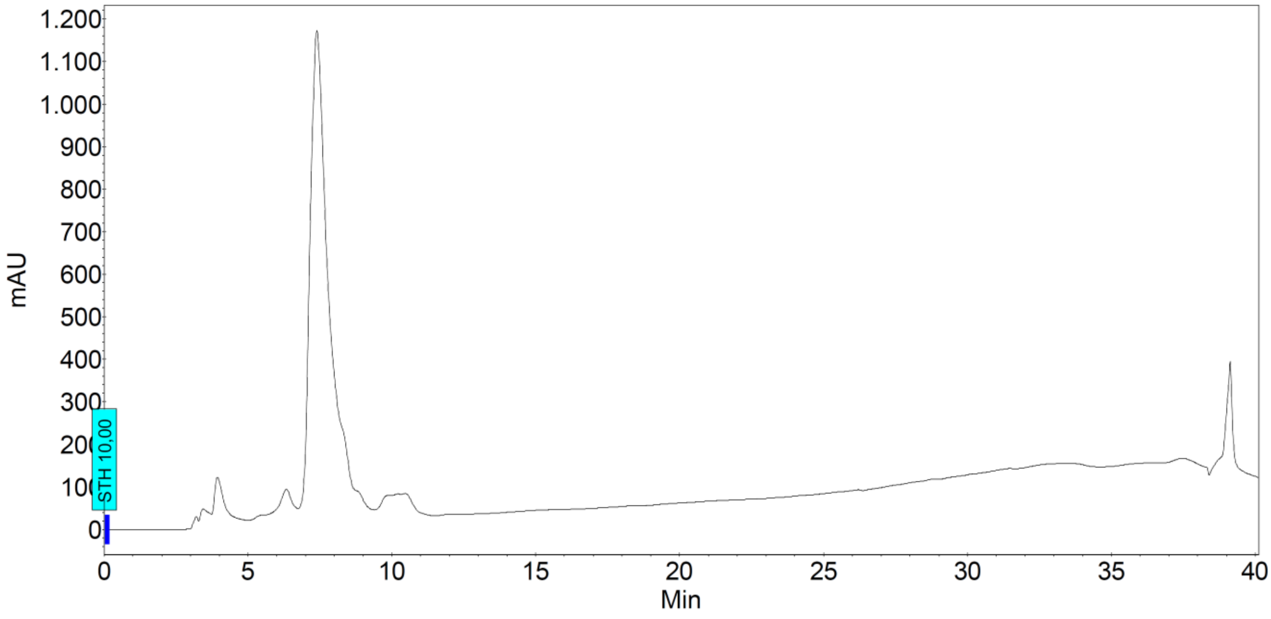

Figure S1. HPLC analysis of **8**

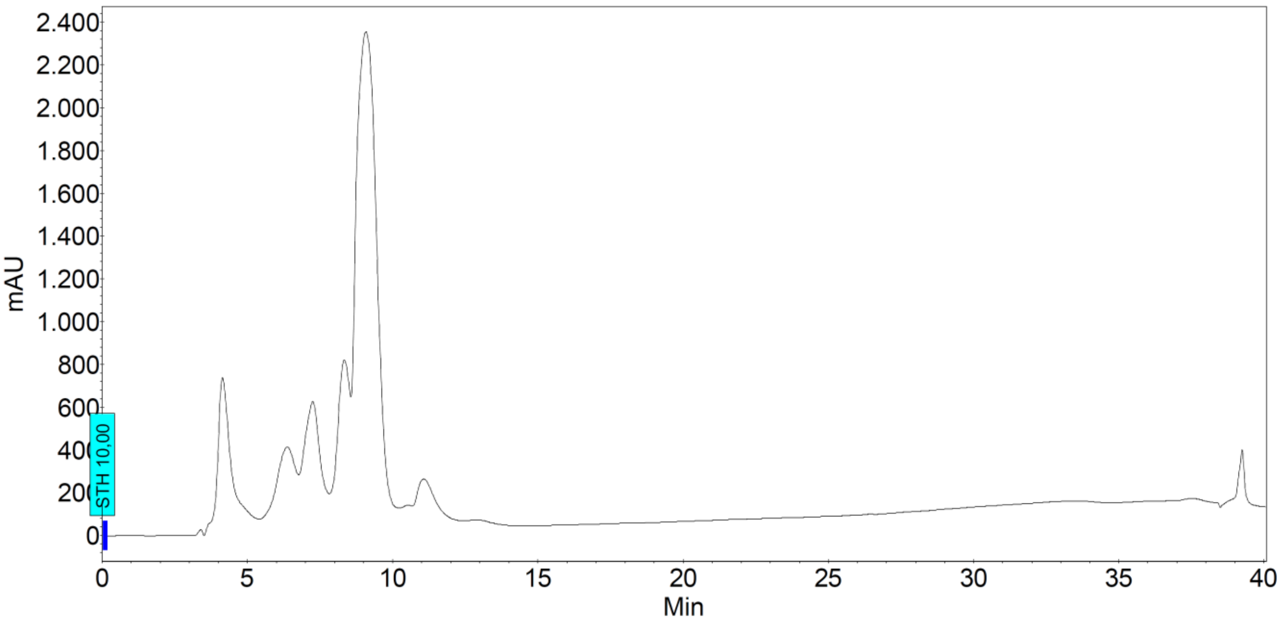

Figure S2. HPLC analysis of **9**

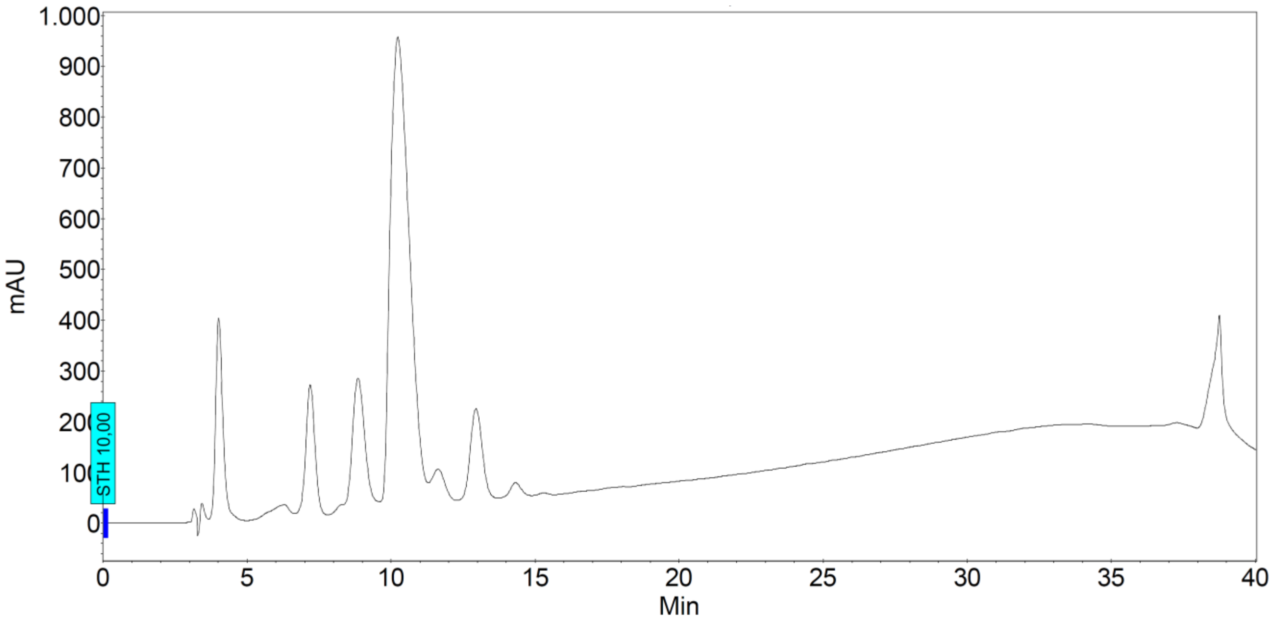

Figure S3. HPLC analysis of **10**

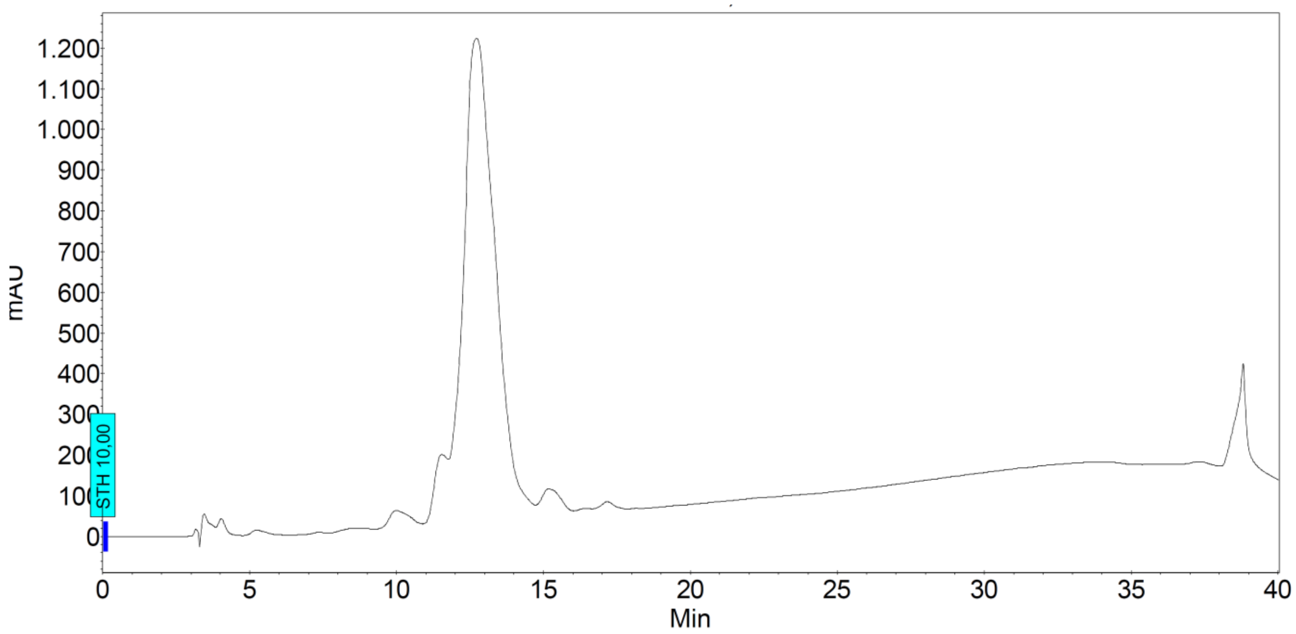

Figure S4. HPLC analysis of **11**

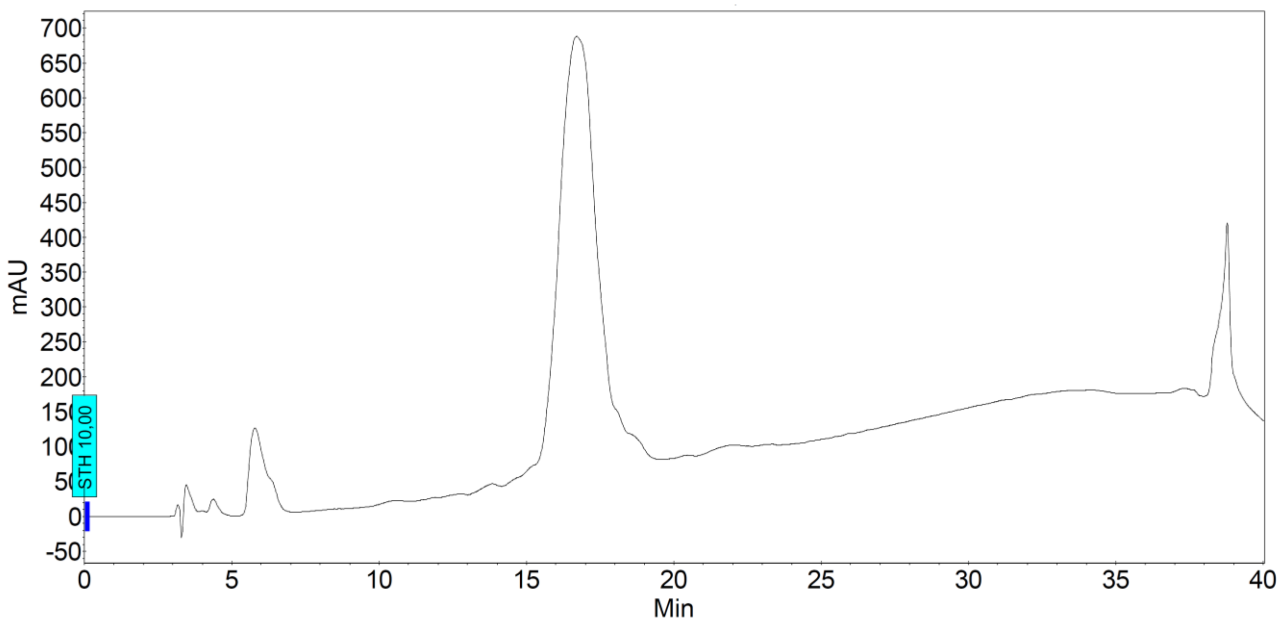

Figure S5. HPLC analysis of **12**

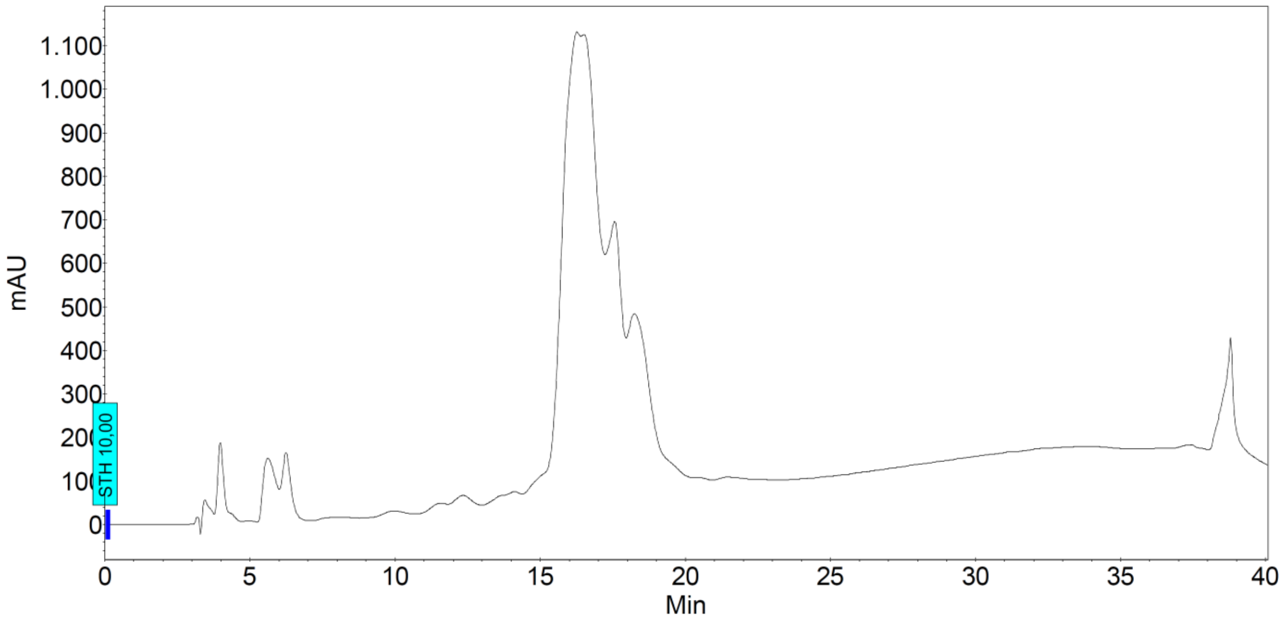

Figure S6. HPLC analysis of **13**

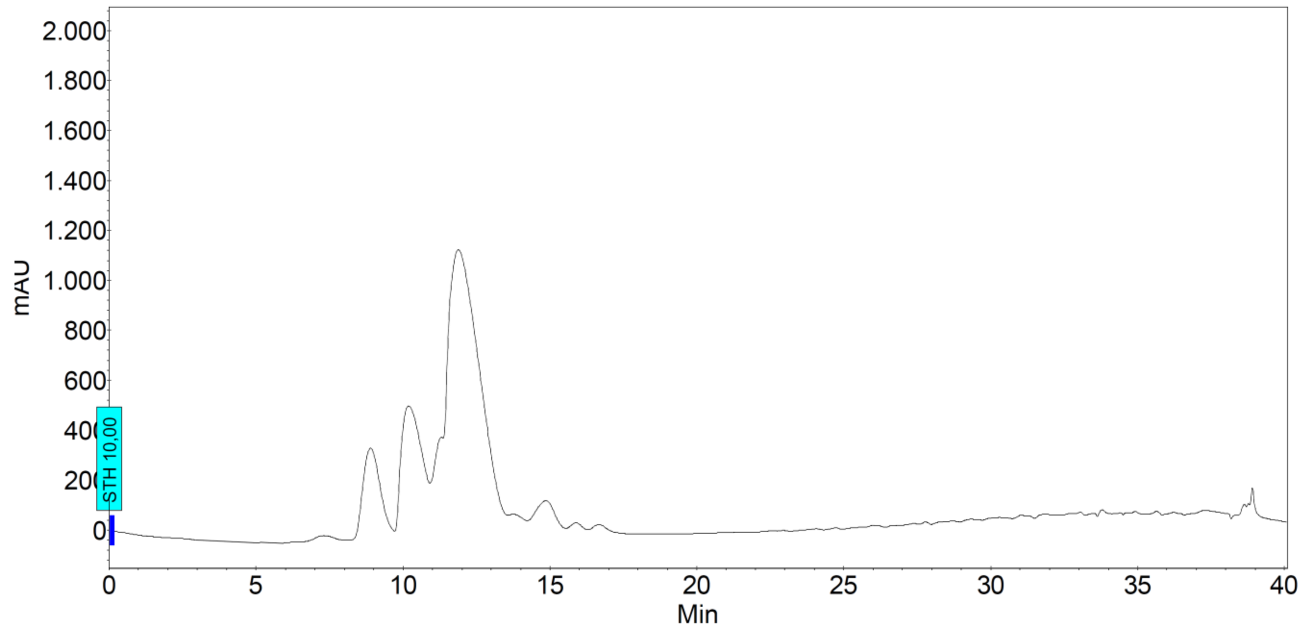

Figure S7. HPLC analysis of **14**

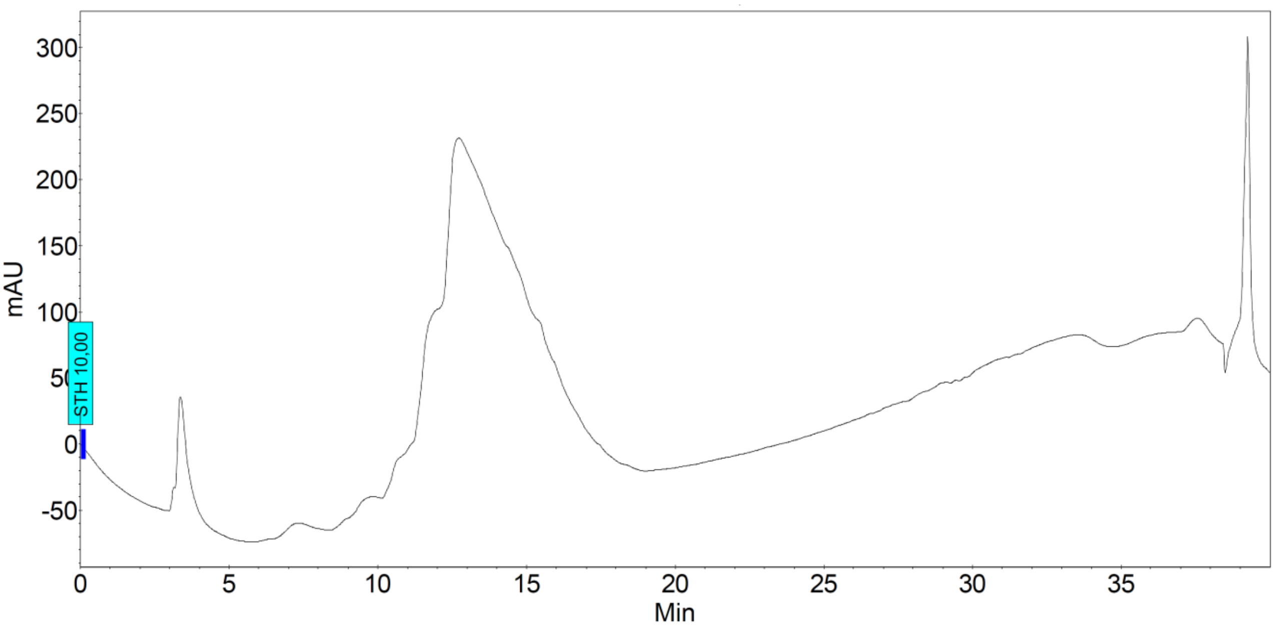

Figure S8. HPLC analysis of **15**

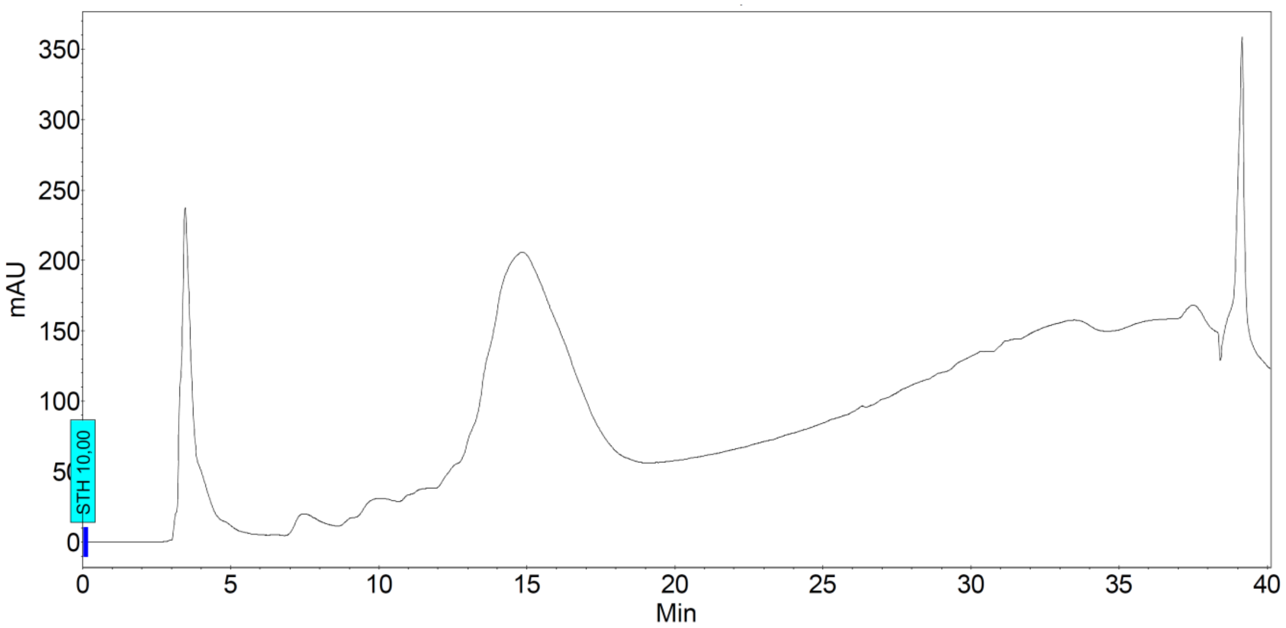

Figure S9. HPLC analysis of **16**

3.2 HPLC chromatograms of cyclic peptoids **17-23**, **1-7** (Figures S10-S23). Partial insolubility of the analytes hampered quantitative evaluation of the impurities.

Conditions: 5 → 100% A in 30 min (A, 0.1% TFA in acetonitrile, B, 0.1% TFA in water); flow: 1 mL min^{-1}, 220 nm.

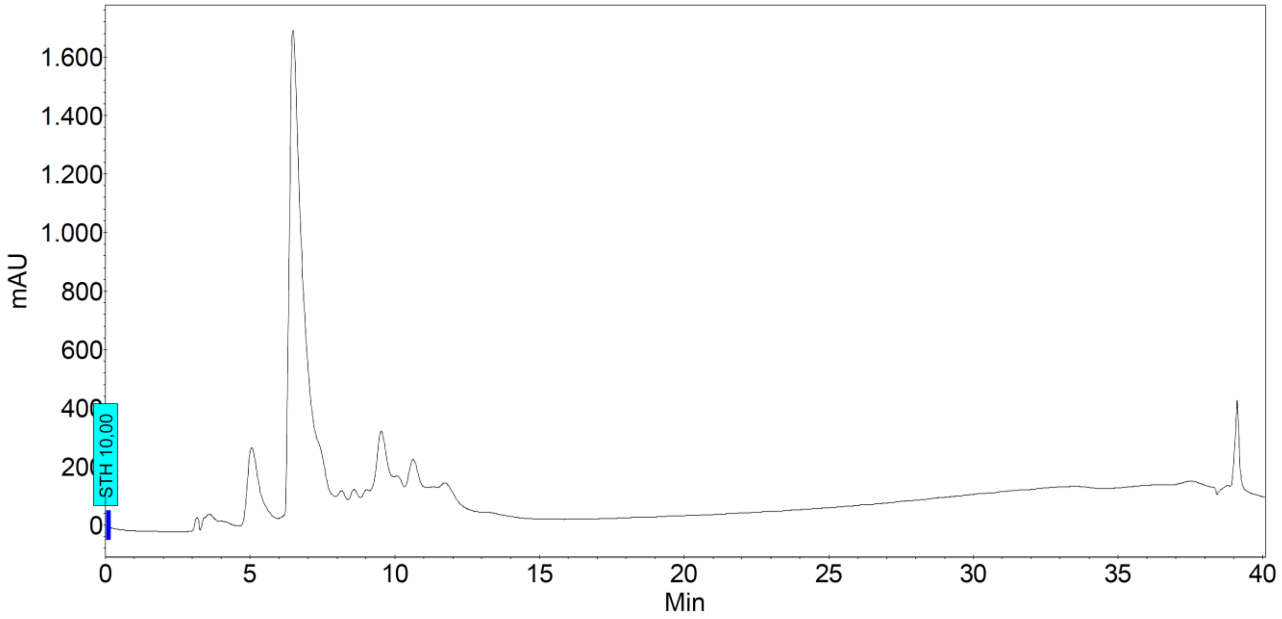

Figure S10. HPLC analysis of **17**

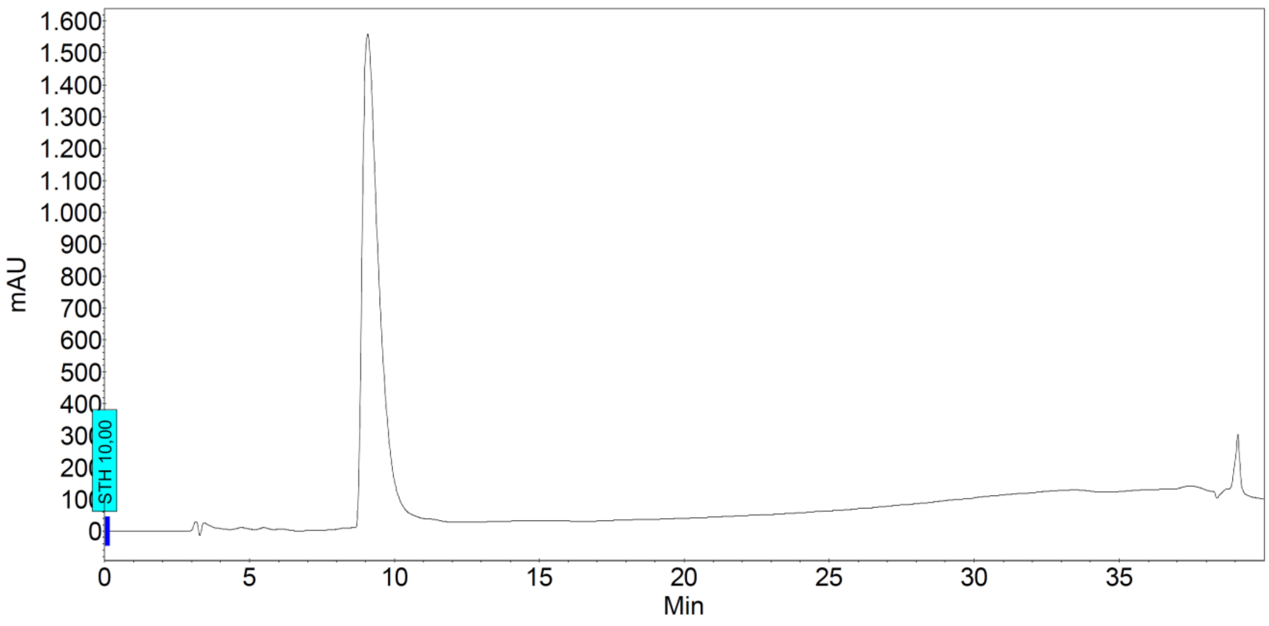

Figure S11. HPLC analysis of **18**

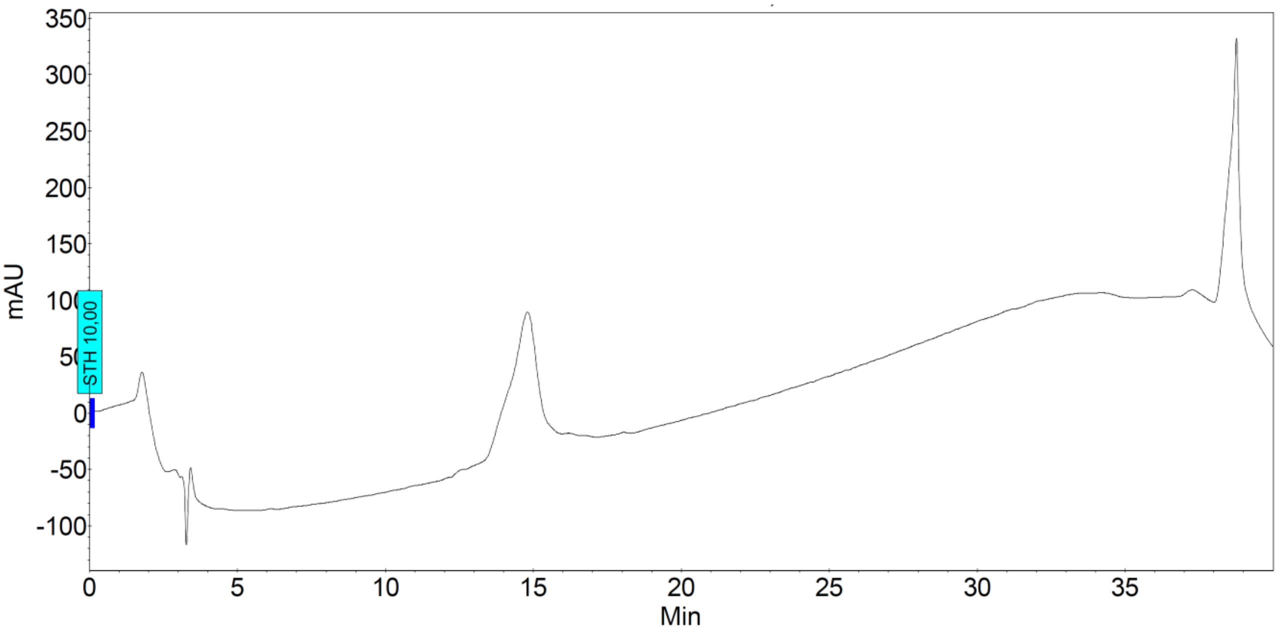

Figure S12. HPLC analysis of **19**

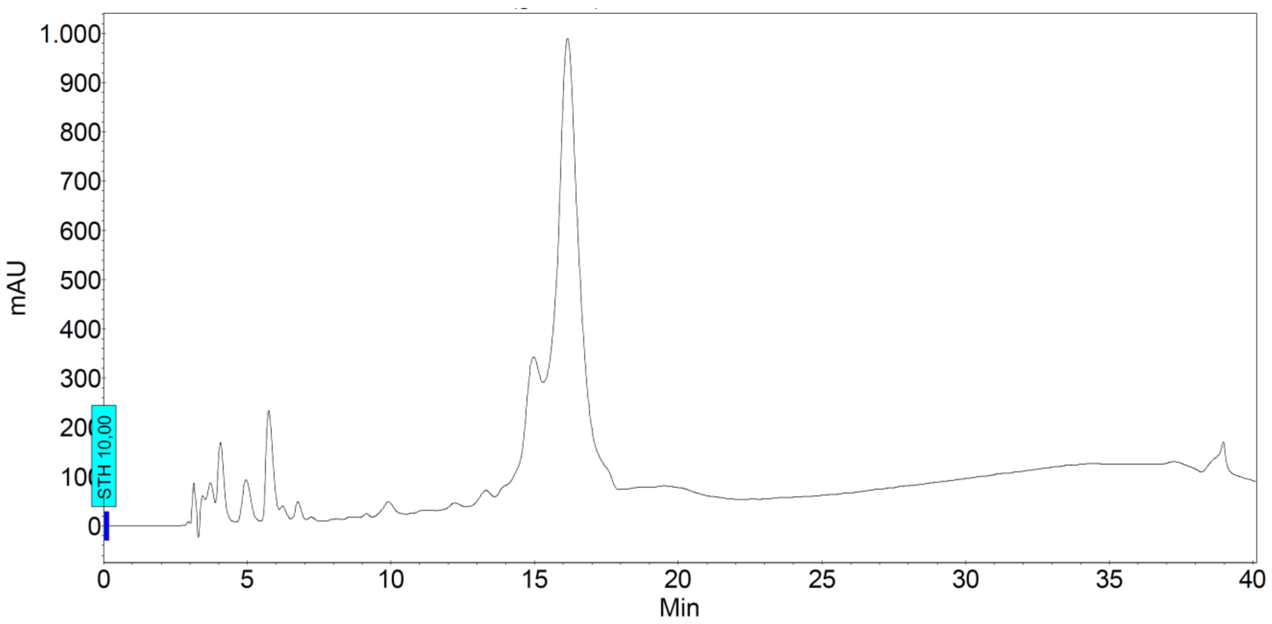

Figure S13. HPLC analysis of **20**

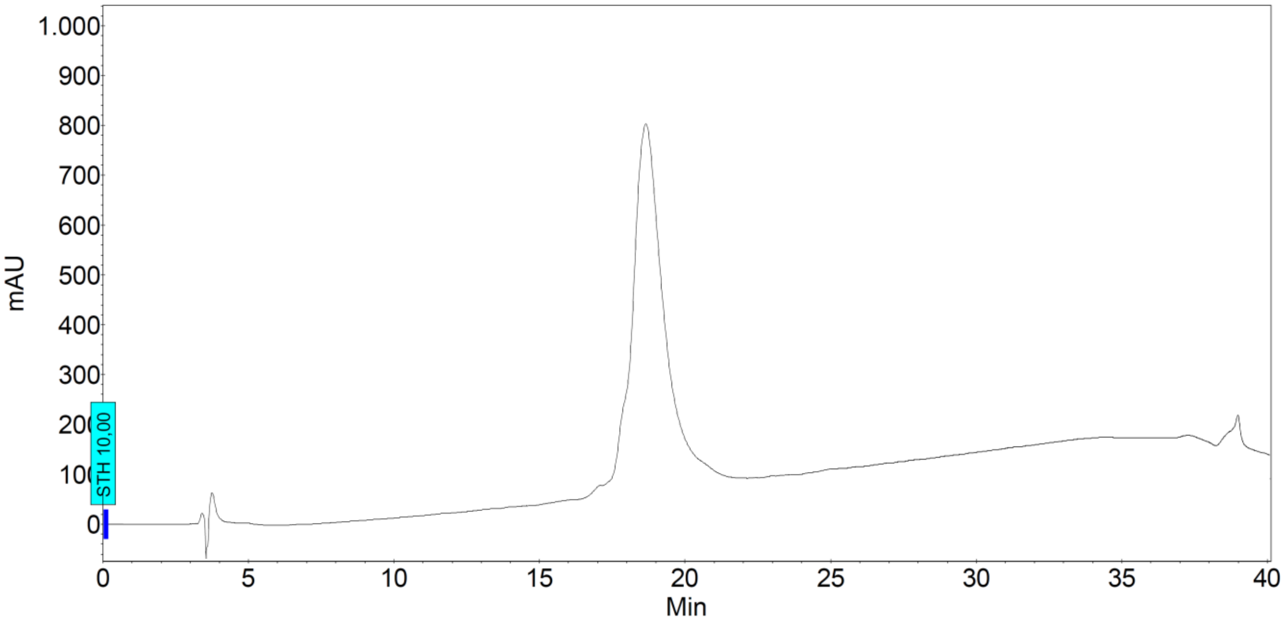

Figure S14. HPLC analysis of **21**

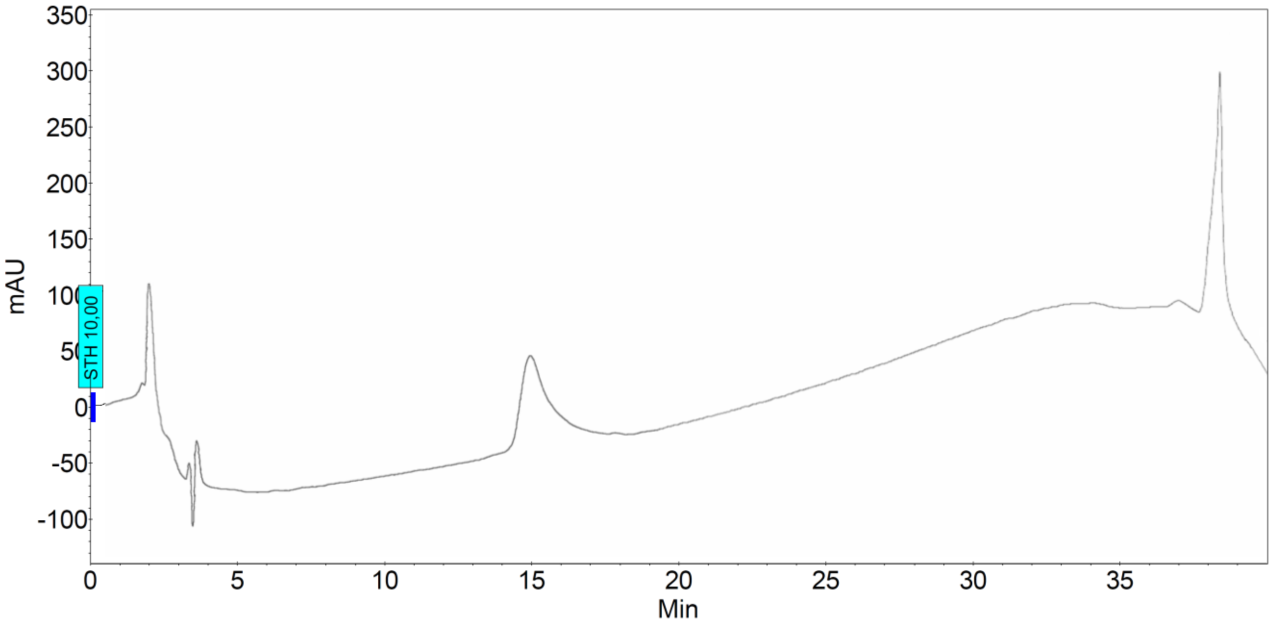

Figure S15. HPLC analysis of **22**

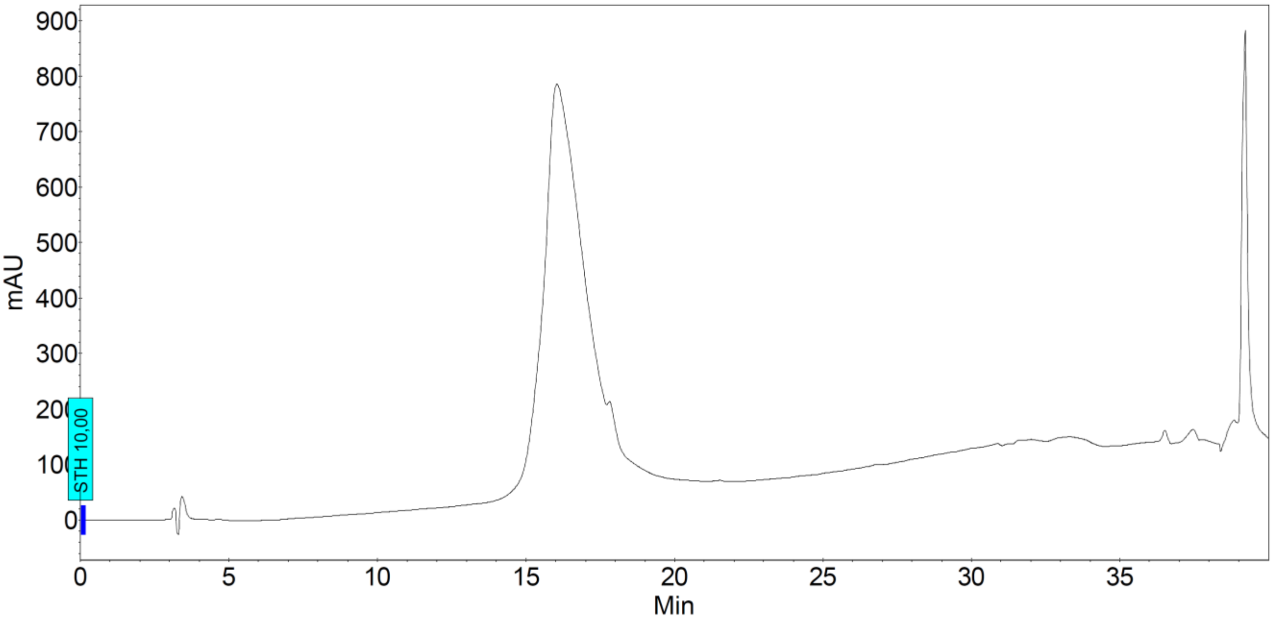

Figure S16. HPLC analysis of **23**

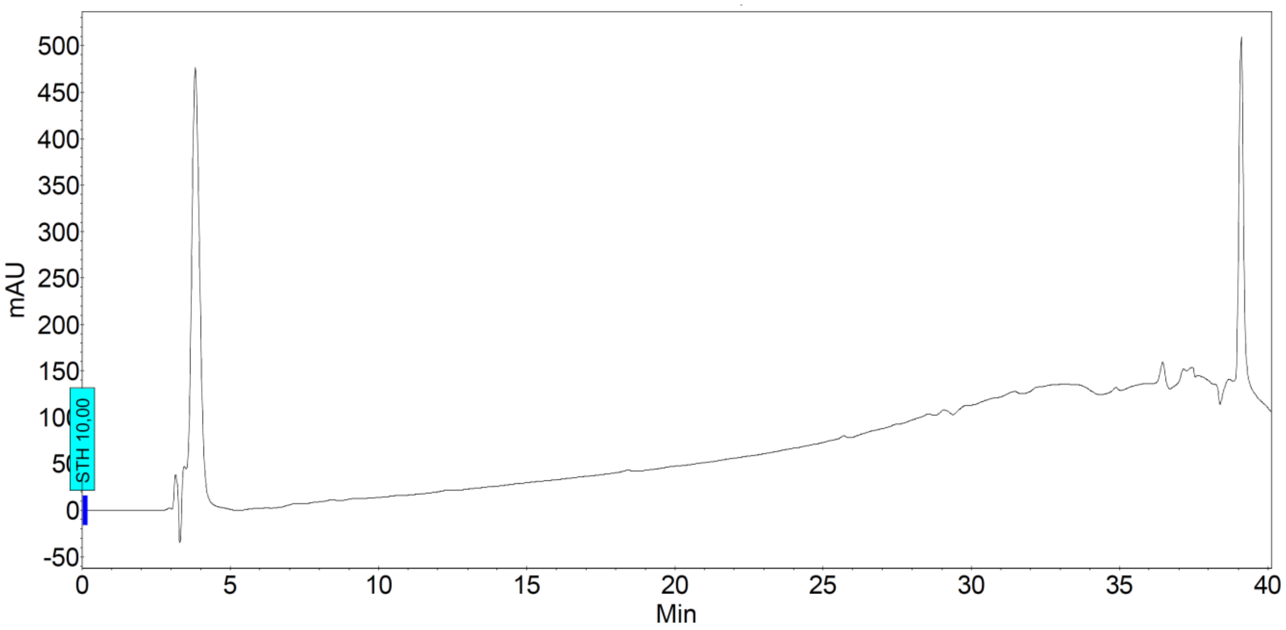

Figure S17. HPLC analysis of **1**

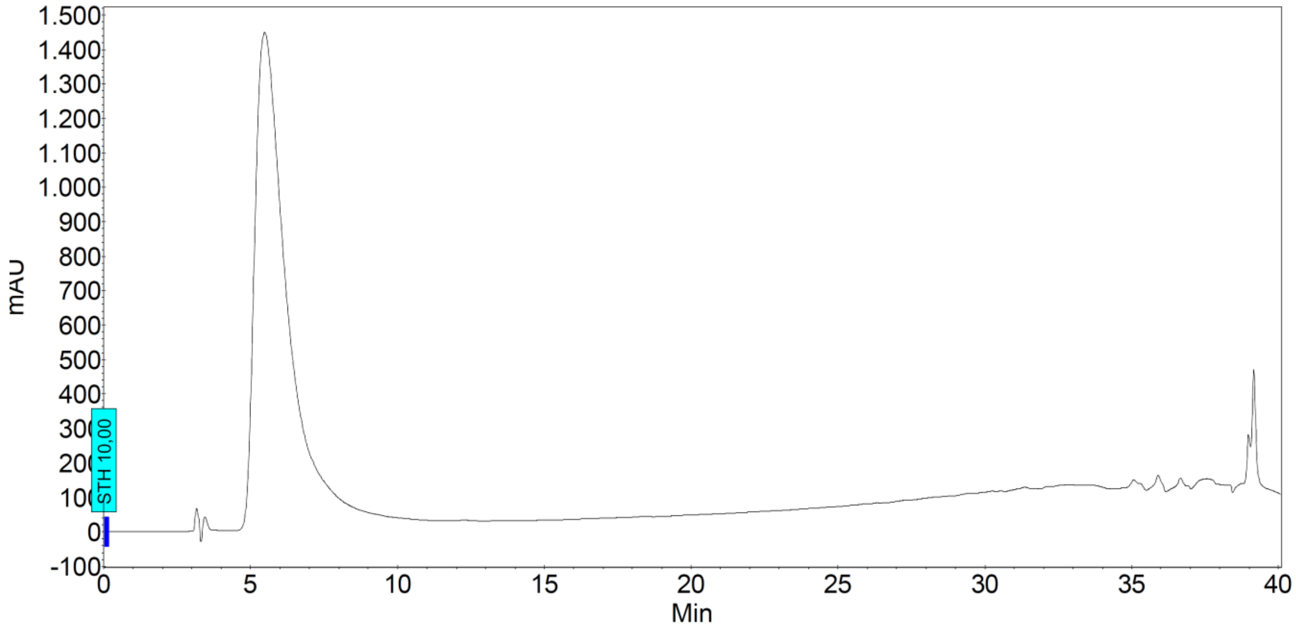

Figure S18. HPLC analysis of **2**

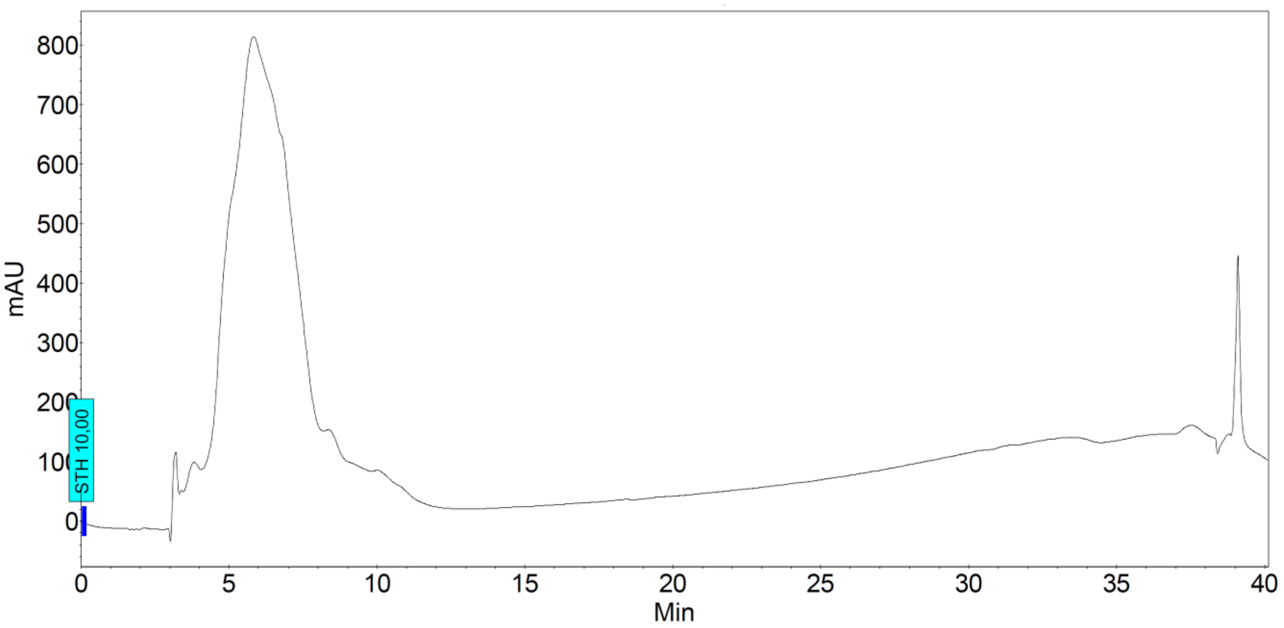

Figure S19. HPLC analysis of **3**

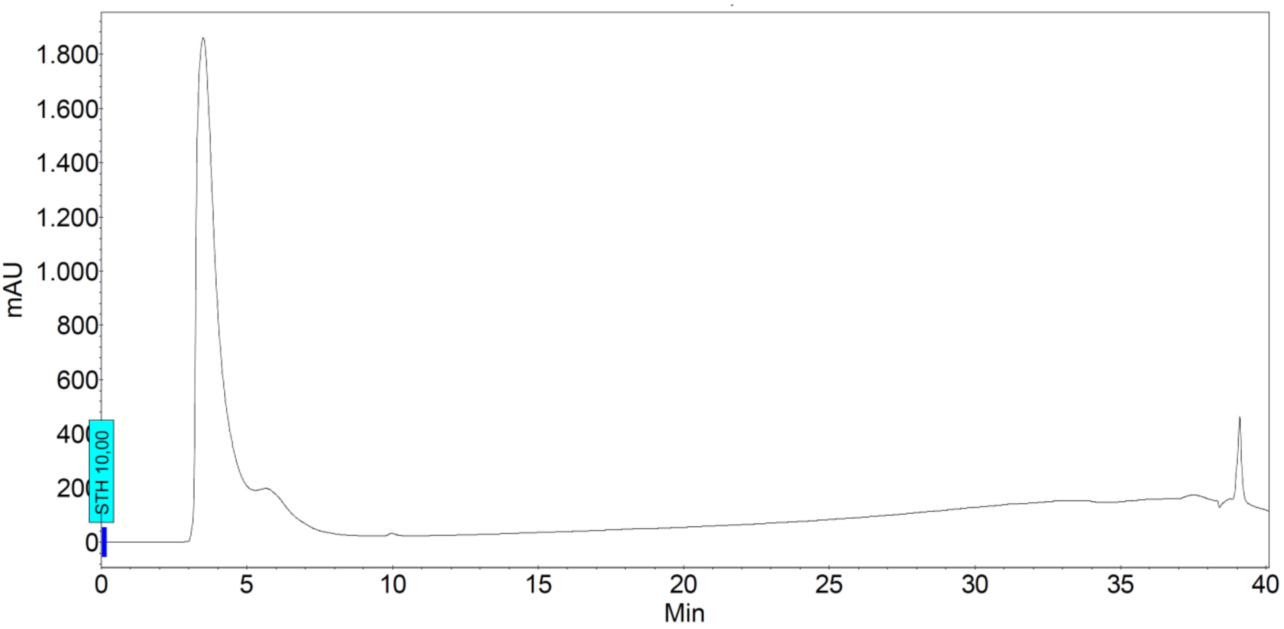

Figure S20. HPLC analysis of **4**

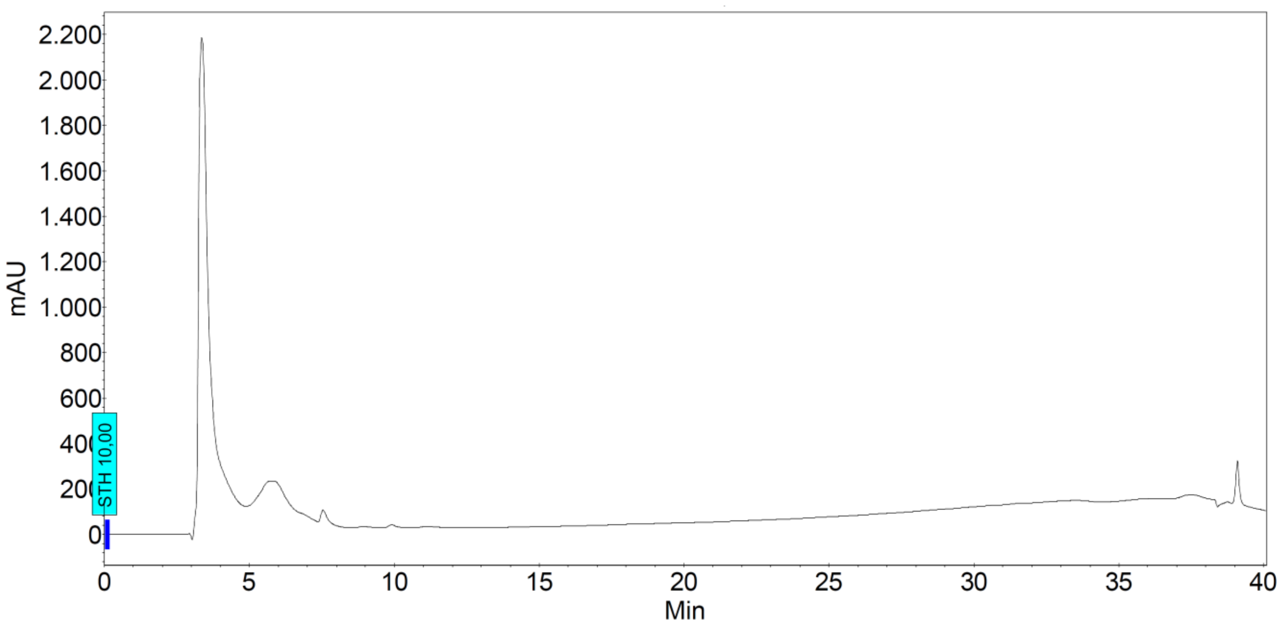

Figure S21. HPLC analysis of **5**

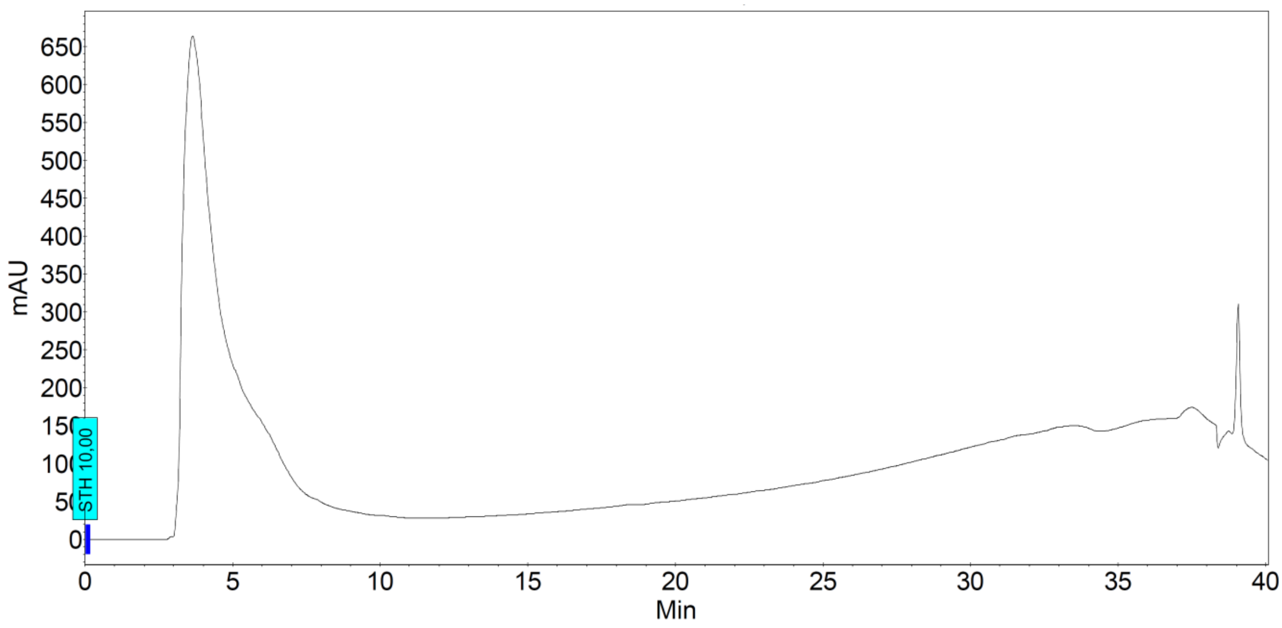

Figure S22. HPLC analysis of **6**

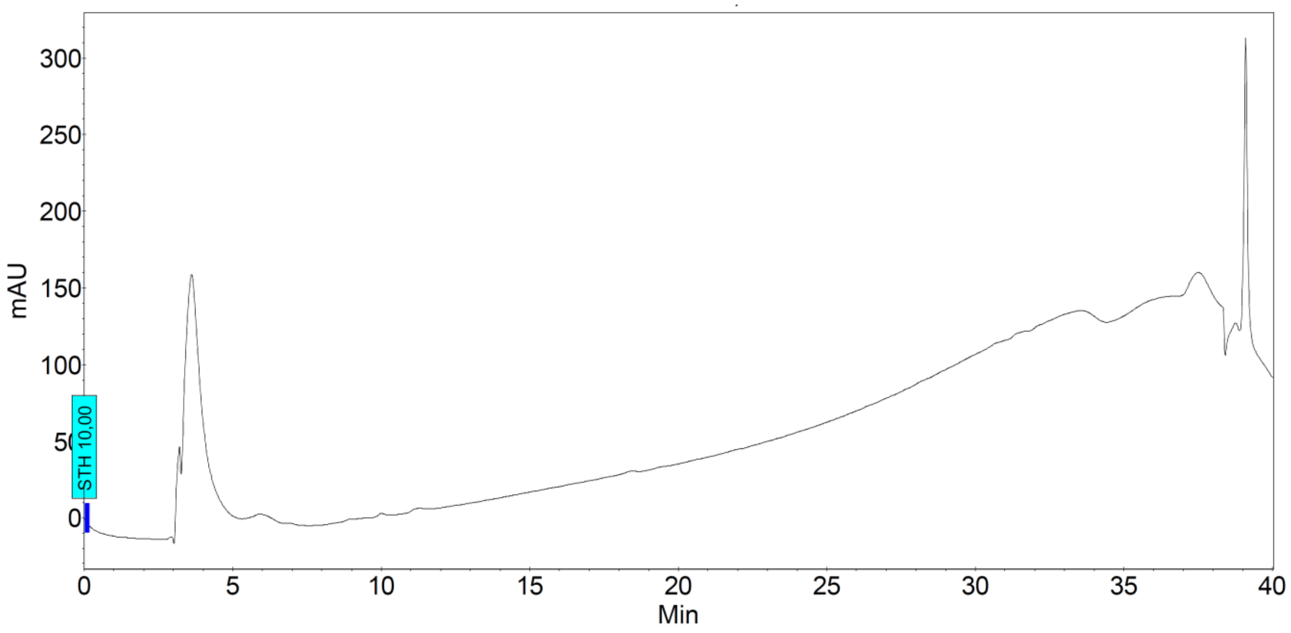

Figure S23. HPLC analysis of **7**

4.0 X-ray crystallographic studies of [1·2K]

The single crystals were prepared as follows: (1) The lyophilized powder of **1** was directly dissolved in proper amount of KOH/H_2O solution (600 mM) to achieve a final concentration ~300 mM and a final pH ~7.5. (2) 0.01 mL of the peptoid solution was placed inside a 20 mL scintillation vial and a parafilm cover (3) The solvent was allowed to slowly evaporate away through a small hole punched on the parafilm cover (4) Plate-like single crystals were obtained within a week.

A colorless and plate-like specimen, approximate dimensions 0.010 mm x 0.230 mm x 0.320 mm, was used for the X-ray crystallographic analysis. The X-ray intensity data were measured at 100(2) K on a Bruker D8 SMART APEXII three-circle diffractometer system equipped with a Incotec microfocus sealed X-ray tube (MoKα, λ = 0.71073 Å) and a multilayer optics monochromator.
A total of 1620 frames were collected. The total exposure time was 16.50 hours. The frames were integrated with the Bruker SAINT software package using a narrow-frame algorithm. The integration of the data using a monoclinic unit cell yielded a total of 40645 reflections to a maximum θ angle of 28.32° (0.75 Å resolution), of which 12468 were independent (average redundancy 3.260, completeness = 99.7%, Rint = 4.23%, Rsig = 4.98%) and 11288 (90.54%) were greater than 2σ(F2). The final cell constants of a = 9.1516(6) Å, b = 8.9718(6) Å, c = 30.934(2) Å, β = 96.102(2)°, volume = 2525.5(3) $Å^3$, are based upon the refinement of the XYZ-centroids of 9954 reflections above 20 σ(I) with 4.531° < 2θ < 56.52°. Data were corrected for absorption effects using the Multi-Scan method (SADABS). The ratio of minimum to maximum apparent transmission was 0.931. The calculated minimum and maximum transmission coefficients (based on crystal size) are 0.8690 and 0.9950.

The structure was solved and refined using the Bruker SHELXTL Software Package, using the space group P 1 21 1, with Z = 4 for the formula unit, $C_{16}H_{34.63}K_2N_4O_{14.41}$. The final anisotropic full-matrix least-squares refinement on F2 with 757 variables converged at R1 = 4.85%, for the observed data and wR2 = 10.71% for all data. The goodness-of-fit was 1.054. The largest peak in the final difference electron density synthesis was 0.705 $e^-/Å^3$ and the largest hole was -0.472 $e^-/Å^3$ with an RMS deviation of 0.069 $e^-/Å^3$. On the basis of the final model, the calculated density was 1.556 g/cm^3 and F(000), 1248 e^-.

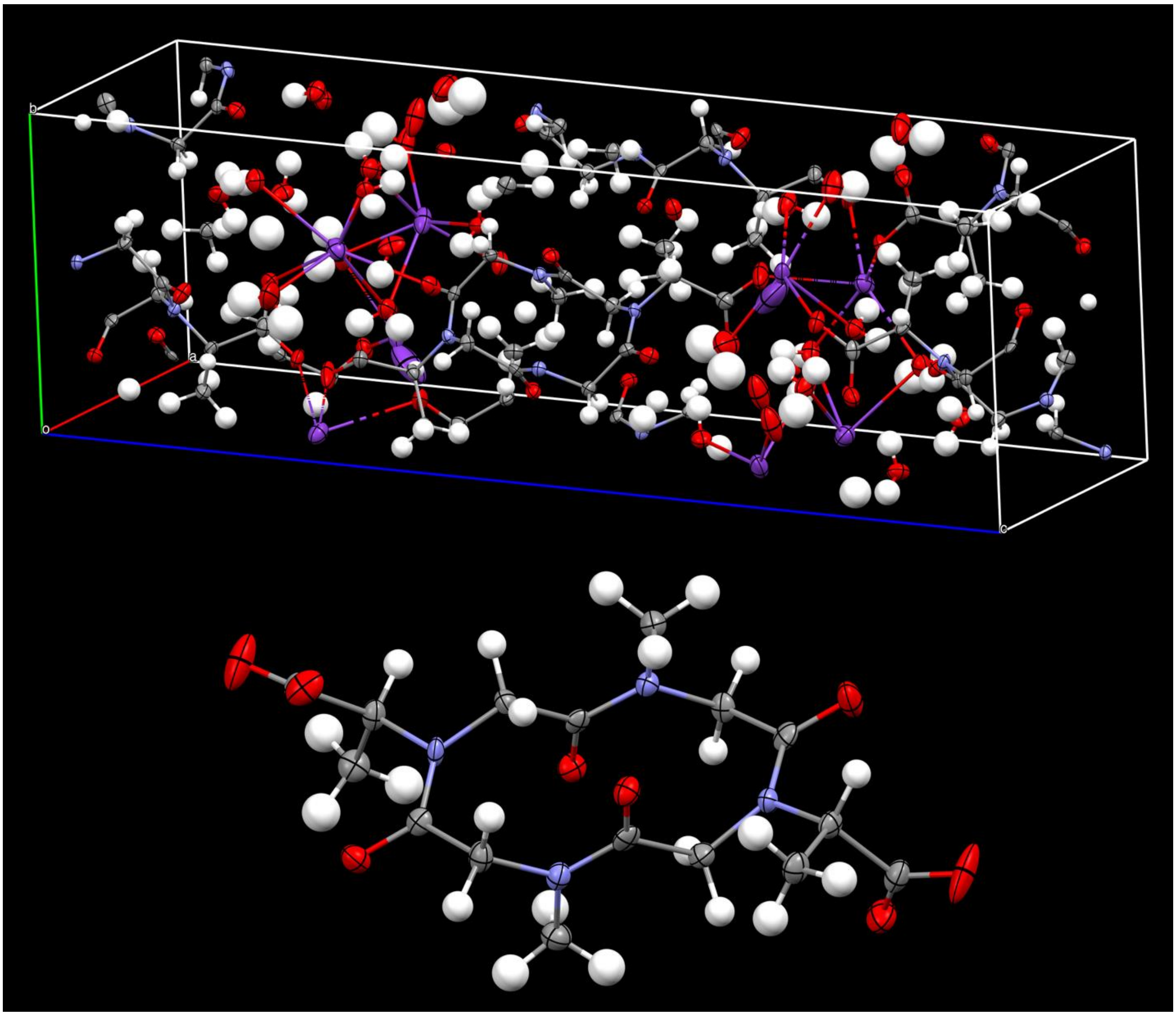

Figure S24. Thermal ellipsoid plots of the potassium complex of **1**: (top) unit cell and (bottom) a single peptoid macrocycle. Ellipsoid contour probability levels were set as 75%. Color code: hydrogen in white, carbon in grey, nitrogen in light blue, oxygen in red and potassium in purple.

Table 1. Crystal data of the potassium complex of **1**.

CCDC number	2218132	
Chemical formula	$C_{16}H_{34.63}K_2N_4O_{14.41}$	
Temperature	100(2) K	
Wavelength	0.71073 Å	
Crystal system	monoclinic	
Space group	P 1 21 1	
Unit cell dimensions	a = 9.1516(6) Å	α = 90°
	b = 8.9718(6) Å	β = 96.102(2)°
	c = 30.934(2) Å	γ = 90°
Structure solution technique	direct methods	
Structure solution program	SHELXT (Sheldrick 2015)	
Refinement method	Full-matrix least-squares on F^2	
Refinement program	SHELXL-2018/3 (Sheldrick, 2018)	
Function minimized	$\Sigma w(F_o^2 - F_c^2)^2$	
Goodness-of-fit on F^2	1.054	
R1 (all data)	0.0551	

5.0 Relaxometric competition with EDTA (Fig. S25)

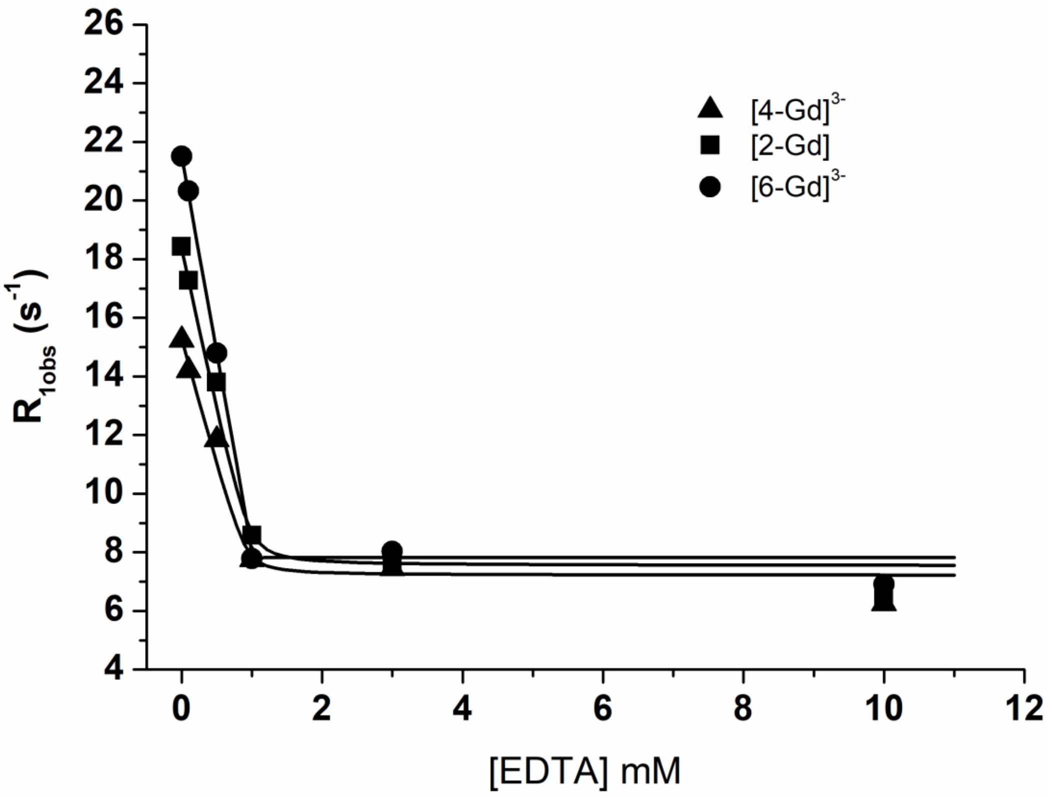

Figure S25: Variation of the observed longitudinal relaxation rate of solutions of [2-Gd] 0.981 mM (■), $[4\text{-Gd}]^{3-}$ 0.936 mM (▲) and $[6\text{-Gd}]^{3-}$ 1.02 mM (●) as a function of the addition of increasing concentrations of EDTA. Measurements were carried out at 25 °C, 21.5 MHz and neutral pH.

6.0 Computational details

The DFT calculations were performed with the Gaussian09 set of programs.[1] For Na^+ complexes we used the BP86 functional of Becke and Perdew[2] and the electronic configuration of the molecular systems was described with the standard triple zeta valence basis set with a polarization function of Ahlrichs and co-workers for H, C, N, O, Na (TZVP keyword in Gaussian).[3] For Gd^{3+} complexes we used PBE0 model[4] and the electronic configuration of the molecular systems was described with 6-31G(d,p) basis set for H, C, N, O [5] and standard SDD basis set for Gd. Scalar relativistic effects in gadolinium have been accounted for using the quasi-relativistic 28-electron effective core potential (MWB28 ECP).[6] The geometry optimizations were performed without symmetry constraints, and the characterization of the located stationary points was performed by analytical frequency calculations. Solvent effects including contributions of non electrostatic terms have been estimated in single-point calculations on the gas phase optimized structures, based on the polarizable continuous solvation model PCM using H_2O as a solvent.[7]

[1] Gaussian 09, Revision A.02, Frisch, M. J.; Trucks, G. W.; Schlegel, H. B.; Scuseria, G. E.; Robb, M. A.; Cheeseman, J. R.; Scalmani, G.; Barone, V.; Mennucci, B.; Petersson, G. A.; Nakatsuji, H.; Caricato, M.; Li, X.; Hratchian, H. P.; Izmaylov, A. F.; Bloino, J.; Zheng, G.; Sonnenberg, J. L.; Hada, M.; Ehara, M.; Toyota, K.; Fukuda, R.; Hasegawa, J.; Ishida, M.; Nakajima, T.; Honda, Y.; Kitao, O.; Nakai, H.; Vreven, T.; Montgomery, J. A., Jr.; Peralta, J. E.; Ogliaro, F.; Bearpark, M.; Heyd, J. J.; Brothers, E.; N. Kudin, K.; Staroverov, V. N.; Kobayashi, R.; Normand, J.; Raghavachari, K.; Rendell, A.; Burant, J. C.; Iyengar, S. S.; Tomasi, J.; Cossi, M.; Rega, N.; Millam, J. M.; Klene, M.; Knox, J. E.; Cross, J. B.; Bakken, V.; Adamo, C.; Jaramillo, J.; Gomperts, R.; Stratmann, R. E.; Yazyev, O.; Austin, A. J.; Cammi, R.; Pomelli, C.; Ochterski, J. W.; Martin, R. L.; Morokuma, K.; Zakrzewski, V. G.; Voth, G. A.; Salvador, P.; Dannenberg, J. J.; Dapprich, S.; Daniels, A. D.; Farkas, O.; Foresman, J. B.; Ortiz, J. V.; Cioslowski, J.; Fox, D. J. Gaussian, Inc., Wallingford CT, **2009**.

[2] a) Becke, A. Phys. Rev. A 1988, 38, 3098– 3100. b) Perdew, J. P. Phys. Rev. B 1986, 33, 8822– 8824. c) Perdew, J. P. *Phys. Rev. B* **1986**, *34*, 7406– 7406.

[3] Schaefer, A., Horn, H. and Ahlrichs, R. *J. Chem. Phys.* **1994**, *100*, 5829– 5835.

[4] C. Adamo and V. Barone, "Toward reliable density functional methods without adjustable parameters: The PBE0 model," *J. Chem. Phys.*, **110** (1999) 6158-69.

[5] a) R. Ditchfield, W. J. Hehre, and J. A. Pople, "Self-Consistent Molecular Orbital Methods. 9. Extended Gaussian-type basis for molecular-orbital studies of organic molecules," *J. Chem. Phys.*, **54** (1971) 724. b) W. J. Hehre, R. Ditchfield, and J. A. Pople, "Self-Consistent Molecular Orbital Methods. 12. Further extensions of Gaussian-type basis sets for use in molecular-orbital studies of organic-molecules," *J. Chem. Phys.*, **56** (1972) 2257. c) M. M. Francl, W. J. Pietro, W. J. Hehre, J. S. Binkley, D. J. DeFrees, J. A. Pople, and M. S. Gordon, "Self-Consistent Molecular Orbital Methods. 23. A polarization-type basis set for 2nd-row elements," *J. Chem. Phys.*, **77** (1982) 3654-65.

[6]a) M. Dolg, H. Stoll, H. Preuss "Energy-Adjusted Ab initio Pseudopotentials for the Rare Earth Elements." The Journal of Chemical Physics *J. Chem. Phys.* **1989**, *90*, 1730-1734. b) X. Cao, M. Dolg, "Segmented contraction scheme for small-core lanthanide pseudopotential basis sets" *J. Mol. Struc-THEOCHEM* **2002**, *581*, 139-147.

[7] a) Barone, V. and Cossi, M. *J. Phys. Chem. A* **1998**, *102*, 1995– 2001. b) Tomasi, J. and Persico, M. *Chem. Rev.* **1994**, *94*, 2027– 2094.

6.1 Cartesian Coordinates and energies of calculated structures

76

[**2**·Na]$^{2-}$ E(gas)= -2329.02161101 E(H_2O)= -2329.25676293 G(H_2O)= -2328.76483793

C	-2.461027	-1.727079	-0.477001
N	-3.557822	-1.059929	-0.042884
C	-3.730738	0.305529	-0.539431
C	-2.739412	1.282307	0.112797
C	-2.156708	-3.131105	0.109065
C	2.726602	-1.267548	-0.474951
N	2.696492	-2.551344	-0.041937
C	1.600826	-3.383093	-0.540606
C	0.258889	-3.014017	0.111536
N	-0.869545	-3.640614	-0.363548
O	1.898716	-0.830478	-1.302072
O	0.180686	-2.187334	1.034760
O	-1.668777	-1.227270	-1.303489
O	-1.984186	0.935872	1.035584
C	3.700001	-3.170825	0.863114
C	-0.870900	-4.426897	-1.595676
C	-4.595533	-1.620307	0.862232
C	-0.265109	2.994768	-0.475944
N	0.861306	3.611042	-0.042473
C	2.130016	3.077933	-0.539529
C	2.480509	1.731558	0.113685
C	-1.632835	3.432757	0.111413
N	3.587959	1.067247	-0.359501
C	3.789160	-0.302179	0.113803
N	-2.717896	2.573207	-0.361294
O	-0.228925	2.058843	-1.302519
O	1.802591	1.251193	1.036461
C	4.271019	1.458075	-1.591181
C	-3.399572	2.968952	-1.592178
C	0.895585	4.790115	0.862291
H	-4.794132	0.563308	-0.356709
H	-3.564325	0.308426	-1.627246
H	-2.939918	-3.851109	-0.169420
H	-2.127375	-3.056741	1.205285
H	1.515507	-3.238150	-1.628140
H	1.909079	-4.433251	-0.359926
H	2.884881	3.870270	-0.358204
H	2.048554	2.931228	-1.627125
H	-1.864964	4.471289	-0.165902
H	-1.582182	3.368954	1.207531
H	3.707077	-0.313849	1.209812
H	4.805017	-0.620475	-0.162023
Na	-0.000060	0.000035	-0.082336
H	-3.585558	4.052852	-1.562944
H	-2.789357	2.737038	-2.482488
H	-4.369190	2.462713	-1.672341
C	1.554732	6.049248	0.142326
H	-0.144883	5.064166	1.066947

C	1.613340	4.464783	2.177094
H	5.302522	1.076733	-1.562028
H	3.764298	1.044483	-2.480538
H	4.317933	2.550808	-1.672840
C	4.461400	-4.370949	0.143372
H	4.457283	-2.406652	1.068386
C	3.058773	-3.630231	2.177516
H	0.052141	-5.013769	-1.676229
H	-1.716823	-5.129690	-1.568098
H	-0.974412	-3.780917	-2.484932
C	-6.016833	-1.677204	0.144736
H	-4.313290	-2.658954	1.064978
C	-4.670508	-0.837766	2.178276
H	1.527735	5.329097	2.854633
H	1.189942	3.573963	2.665284
H	2.684697	4.295710	1.994899
O	0.795167	7.039621	-0.004671
O	2.774246	5.911102	-0.179294
H	3.849659	-3.988700	2.855301
H	2.498880	-2.818197	2.665627
H	2.376552	-4.473209	1.994538
O	5.699090	-4.208556	-0.002029
O	3.732214	-5.357757	-0.179449
H	-5.377071	-1.343453	2.855371
H	-3.686989	-0.761698	2.666216
H	-5.057409	0.175703	1.997454
O	-6.496912	-2.829539	-0.000216
O	-6.506004	-0.551513	-0.176629

76

[**2a**·Na]$^{2-}$ E(gas) -2329.00771758 E(H_2O)= -2329.24389436 G(H_2O)= -2328.75371236

C	2.121389	2.168775	0.108785
N	1.599225	3.333191	-0.390558
C	0.296925	3.761659	0.124611
C	-0.835592	2.888981	-0.449146
C	3.381860	1.596684	-0.592348
C	0.817433	-2.921268	0.108758
N	2.087338	-3.051693	-0.389363
C	3.109075	-2.138065	0.126662
C	2.919648	-0.721243	-0.447801
N	3.758516	0.283129	-0.074985
O	0.573600	-2.198019	1.086771
O	1.957922	-0.497148	-1.214536
O	1.616439	1.596169	1.086665
O	-0.549244	1.943717	-1.215631
C	2.362451	-3.681309	-1.682210
C	5.002966	0.160412	0.733333
C	2.007872	3.885672	-1.683410
C	-2.939327	0.752927	0.110132
N	-3.687196	-0.281663	-0.388036
C	-3.406160	-1.623532	0.127184
C	-2.084680	-2.167542	-0.448258

C	-3.074208	2.130164	-0.591766
N	-1.633995	-3.396321	-0.076070
C	-0.308008	-3.726133	-0.593588
N	-2.124596	3.113193	-0.075310
O	-2.190448	0.602413	1.087688
O	-1.410079	-1.446060	-1.214665
C	-2.362101	-4.413040	0.732130
C	-2.639870	4.252020	0.733723
C	-4.370610	-0.204966	-1.680622
H	0.099959	4.808863	-0.151964
H	0.306251	3.660054	1.217353
H	4.231677	2.281172	-0.451335
H	3.173701	1.520691	-1.670948
H	3.015512	-2.095069	1.219341
H	4.114713	-2.490967	-0.149173
H	-4.214774	-2.317922	-0.148279
H	-3.321155	-1.564356	1.219803
H	-4.091805	2.524171	-0.450809
H	-2.904604	1.987214	-1.670331
H	-0.269717	-3.506278	-1.671887
H	-0.140089	-4.804551	-0.454133
Na	-0.000256	0.000435	0.053700
C	-2.380822	5.714230	0.129845
H	-3.733716	4.142894	0.683389
C	-2.261070	4.171237	2.222398
H	-3.655661	-0.295170	-2.516001
H	-5.084988	-1.036414	-1.742914
H	-4.927849	0.737252	-1.774402
C	-3.758817	-4.918545	0.129207
H	-1.721226	-5.306054	0.679504
C	-2.479611	-4.047054	2.221602
H	2.083665	-3.016368	-2.517247
H	3.439586	-3.884843	-1.744431
H	1.824472	-4.634605	-1.776564
C	4.744630	-0.126284	2.222407
C	6.139469	-0.795494	0.129686
H	5.455521	1.162166	0.682132
H	1.572685	3.310828	-2.518467
H	1.644921	4.919968	-1.746671
H	3.102526	3.897191	-1.776767
H	-2.856214	-4.931341	2.753063
H	-1.506875	-3.740646	2.635768
H	-3.197884	-3.232432	2.396233
O	-4.225064	-5.893258	0.773617
O	-4.215589	-4.322530	-0.883867
O	7.217092	-0.711032	0.773377
O	5.851493	-1.489445	-0.883091
H	5.698510	-0.009802	2.754186
H	3.992123	0.561700	2.637204
H	4.399437	-1.156242	2.395868
O	-2.991159	6.605485	0.774748

O	-1.637850	5.811468	-0.884416
H	-2.838406	4.938936	2.754888
H	-2.480153	3.175336	2.636880
H	-1.196342	4.387184	2.395100

91

[**4**·Na]$^{5-}$ E(gas)=-3010.78585472 E(H_2O)= -3011.78910364 G(H_2O)= -3011.23303264

C	1.411107	-2.692372	0.235775
N	2.664277	-2.635240	-0.296423
C	3.523830	-1.555824	0.206958
C	3.052760	-0.166684	-0.290648
C	0.406038	-3.742405	-0.318743
C	-3.037621	0.123787	0.234837
N	-3.614622	-0.990032	-0.297547
C	-3.109763	-2.274094	0.205967
C	-1.670957	-2.560482	-0.291080
N	-0.946543	-3.595909	0.237635
O	-2.170140	0.042897	1.128152
O	-1.161953	-1.827665	-1.164756
O	1.047244	-1.900466	1.128828
O	2.163355	-0.092353	-1.164071
C	-4.692594	-0.975618	-1.311401
C	-1.374255	-4.595893	1.254083
C	3.191059	-3.576071	-1.310071
C	1.625875	2.567875	0.235451
N	0.949639	3.624539	-0.296728
C	-0.414823	3.829241	0.206796
C	-1.382259	2.726788	-0.290987
C	3.037995	2.222991	-0.318878
N	-2.641546	2.617412	0.236917
C	-3.444469	1.519227	-0.319634
N	3.587780	0.978497	0.237370
O	1.122095	1.856611	1.128418
O	-1.001849	1.919657	-1.164637
C	-3.294026	3.487717	1.253267
C	4.668858	1.108314	1.252497
C	1.501096	4.551328	-1.310169
H	4.561984	-1.720816	-0.109101
H	3.499183	-1.577904	1.302364
H	0.763592	-4.757626	-0.096269
H	0.352568	-3.625425	-1.411736
H	-3.116950	-2.241738	1.301359
H	-3.771502	-3.090748	-0.110420
H	-0.791148	4.810805	-0.109132
H	-0.383267	3.818859	1.302196
H	3.738080	3.040429	-0.095987
H	2.963800	2.118550	-1.411926
H	-3.316242	1.507146	-1.412600
H	-4.502508	1.717166	-0.097343
Na	-0.000227	-0.000212	-0.001677
C	6.139296	0.689003	0.787580
H	4.749041	2.195046	1.403787

C	4.311609	0.529426	2.635175
C	2.004331	5.965778	-0.749565
H	2.416303	4.078874	-1.694797
C	0.546099	4.713960	-2.503928
C	-3.666191	4.971309	0.790139
H	-4.275197	3.013686	1.404518
C	-2.613222	3.466182	2.635567
C	-6.169353	-1.247098	-0.751401
H	-4.740925	0.053349	-1.695690
C	-4.355348	-1.883411	-2.505439
C	-1.696480	-3.995292	2.636165
C	-2.472846	-5.660086	0.790823
H	-0.473161	-5.208520	1.405752
C	4.166057	-4.717971	-0.750117
H	2.324442	-4.133399	-1.693632
C	3.807414	-2.830053	-2.504738
H	1.038359	5.371758	-3.234197
H	0.307829	3.738182	-2.954808
H	-0.400598	5.189528	-2.204839
O	2.432865	6.737690	-1.663640
O	1.971719	6.153270	0.492895
H	-5.170622	-1.785451	-3.236206
H	-3.390794	-1.601701	-2.955413
H	-4.294192	-2.941252	-2.207016
O	-7.051629	-1.263063	-1.665920
O	-6.316003	-1.368058	0.491097
H	4.131023	-3.585070	-3.235181
H	3.080265	-2.136728	-2.955019
H	4.692226	-2.246841	-2.206744
O	4.619508	-5.475234	-1.664410
O	4.346480	-4.782694	0.492138
H	-3.323812	3.887187	3.361404
H	-2.344100	2.436702	2.916481
H	-1.707810	4.088826	2.671518
O	-4.372817	5.584902	1.648104
O	-3.269315	5.363742	-0.336826
H	5.033207	0.933452	3.359789
H	3.286288	0.812027	2.918041
H	4.397182	-0.566092	2.670635
O	7.025080	0.994995	1.644135
O	6.279363	0.148285	-0.339170
H	-1.705808	-4.821056	3.362131
H	-0.939766	-3.247234	2.917173
H	-2.688535	-3.522725	2.671779
O	-2.650989	-6.578847	1.648767
O	-3.011033	-5.512573	-0.336188

91

[**6**·Na]$^{5-}$ E(gas)-3010.81225377 E(H_2O)= -3011.8054958 G(H_2O)= -3011.2459818

C	-1.983270	2.306316	0.282617
N	-3.204159	1.945195	-0.214777
C	-3.751792	0.672731	0.269515

C	-2.989094	-0.564386	-0.282653
C	-1.293166	3.585315	-0.269626
C	2.989060	0.564361	0.282687
N	3.286786	1.802228	-0.214745
C	2.458606	2.912754	0.269470
C	1.005894	2.870700	-0.282694
N	0.082594	3.747289	0.214790
O	2.102965	0.420691	1.148623
O	0.687422	2.031543	-1.148750
O	-1.415791	1.610758	1.148535
O	-2.102999	-0.420732	-1.148601
C	4.333792	2.083698	-1.223862
C	0.362113	4.794688	1.224043
C	-3.971444	2.711137	-1.223935
C	-1.005900	-2.870668	0.282721
N	-0.082593	-3.747283	-0.214733
C	1.293164	-3.585293	0.269697
C	1.983300	-2.306339	-0.282584
C	-2.458636	-2.912771	-0.269405
N	3.204165	-1.945210	0.214835
C	3.751809	-0.672752	-0.269448
N	-3.286804	-1.802237	0.214802
O	-0.687441	-2.031504	1.148767
O	1.415834	-1.610774	-1.148515
C	3.971453	-2.711170	1.223965
C	-4.333828	-2.083697	1.223892
C	-0.362090	-4.794687	-1.223999
H	-4.817610	0.652811	0.005957
H	-3.691313	0.662599	1.367122
H	-1.843292	4.498456	-0.006259
H	-1.271538	3.527677	-1.367217
H	2.419616	2.865530	1.367077
H	2.974267	3.845710	0.005823
H	1.843250	-4.498489	0.006438
H	1.271501	-3.527548	1.367283
H	-2.974228	-3.845736	-0.005656
H	-2.419720	-2.865621	-1.367015
H	3.691433	-0.662645	-1.367059
H	4.817599	-0.652766	-0.005786
Na	-0.000002	-0.000002	-0.000423
C	-5.480943	-3.101166	0.762386
C	-3.713801	-2.521691	2.562418
H	-4.866760	-1.138223	1.391537
C	-0.054780	-6.296899	-0.762575
H	-1.447283	-4.783364	-1.392111
C	0.327919	-4.476742	-2.562194
C	5.426129	-3.196007	0.762482
C	4.040926	-1.954986	2.562364
H	3.419049	-3.645378	1.391790
C	5.480960	3.101101	-0.762406
H	4.866666	1.138217	-1.391620

C	3.713734	2.521857	-2.562325
H	1.447328	4.783408	1.392047
C	0.054734	6.296897	0.762593
C	-0.327745	4.476672	2.562295
C	-5.426083	3.196070	-0.762504
H	-3.418994	3.645291	-1.391881
C	-4.041059	1.954837	-2.562267
H	0.058111	-5.265251	-3.279288
H	0.031474	-3.485999	-2.938523
H	1.424006	-4.475051	-2.451480
O	-0.321344	-7.152774	-1.660251
O	0.386961	-6.486040	0.401354
H	4.531719	2.682579	-3.279148
H	3.004333	1.769450	-2.938778
H	3.163886	3.470096	-2.451983
O	6.355352	3.298481	-1.660117
O	5.423879	3.578048	0.401594
H	-4.588946	2.582906	-3.279281
H	-3.034817	1.716195	-2.938567
H	-4.587728	1.004778	-2.451757
O	-6.034046	3.854801	-1.660201
O	-5.810717	2.908129	0.401443
O	-6.355071	-3.299040	1.660245
O	-5.424174	-3.577490	-0.401877
H	-4.531814	-2.682500	3.279187
H	-3.163818	-3.469863	2.452167
H	-3.004528	-1.769165	2.938875
O	6.034451	-3.854146	1.660369
O	5.810388	-2.908607	-0.401715
H	4.588921	-2.583028	3.279317
H	4.587447	-1.004830	2.451950
H	3.034641	-1.716540	2.938675
O	0.320712	7.152772	1.660442
O	-0.386322	6.486020	-0.401593
H	-0.057994	5.265236	3.279347
H	-1.423841	4.474841	2.451668
H	-0.031142	3.485972	2.938615

91

[**6a**·Na]$^{5-}$ E(gas)=-3010.77924421 E(H_2O)= -3011.7804778 G(H_2O)= -3011.2215908

C	2.572080	1.635542	-0.258409
N	3.616410	0.940335	0.285687
C	3.793936	-0.427685	-0.229857
C	2.702643	-1.409435	0.258150
C	2.267201	3.071330	0.229935
C	-2.702675	1.409353	-0.258218
N	-2.622480	2.661311	0.285993
C	-1.526410	3.498932	-0.229630
C	-0.130530	3.044854	0.258475
N	0.993717	3.601541	-0.285758
O	-1.903084	1.055571	-1.150048
O	-0.036917	2.175486	1.150263

O	1.865999	1.119911	-1.150274
O	1.902894	-1.055664	1.149833
C	-3.462775	3.222177	1.380923
C	1.059312	4.609522	-1.380831
C	4.522084	1.387698	1.380732
C	0.130661	-3.044910	-0.258509
N	-0.993630	-3.601417	0.285799
C	-2.267079	-3.071238	-0.229992
C	-2.572082	-1.635474	0.258347
C	1.526511	-3.499079	0.229585
N	-3.616516	-0.940371	-0.285708
C	-3.794039	0.427681	0.229772
N	2.622620	-2.661454	-0.285972
O	0.037132	-2.175653	-1.150418
O	-1.865976	-1.119712	1.150115
C	-4.522222	-1.387745	-1.380733
C	3.463010	-3.222355	-1.380817
C	-1.059293	-4.609336	1.380918
H	4.766483	-0.827849	0.090210
H	3.785543	-0.399310	-1.327600
H	3.099960	3.713791	-0.089694
H	2.238158	3.077842	1.327671
H	-1.546690	3.477257	-1.327368
H	-1.666114	4.541353	0.090164
H	-3.099860	-3.713733	0.089521
H	-2.237891	-3.077705	-1.327724
H	1.666192	-4.541468	-0.090320
H	1.546788	-3.477536	1.327324
H	-3.785800	0.399385	1.327516
H	-4.766535	0.827856	-0.090429
Na	0.000007	-0.000012	-0.000345
C	5.036315	-3.342440	-1.118188
H	3.153266	-4.278091	-1.423596
C	3.124940	-2.625144	-2.760358
H	-2.128466	-4.868968	1.423625
C	-0.376585	-6.031991	1.118792
C	-0.711428	-4.017528	2.760354
C	-5.412526	-2.690520	-1.118485
H	-5.281850	-0.591834	-1.423469
C	-3.835938	-1.393065	-2.760266
H	-3.153006	4.277911	1.423733
C	-5.036099	3.342370	1.118353
C	-3.124617	2.624853	2.760382
C	0.711408	4.017798	-2.760302
C	0.376528	6.032125	-1.118640
H	2.128468	4.869200	-1.423583
H	5.281881	0.591928	1.423312
C	5.412201	2.690649	1.118553
C	3.835818	1.392707	2.760259
H	-4.609326	-1.567775	-3.521917
H	-3.321517	-0.436475	-2.935614

H	-3.087983	-2.194924	-2.850914
O	-6.152285	-2.987174	-2.106219
O	-5.350188	-3.246787	0.009790
O	0.489132	6.820847	-2.106608
O	-0.135832	6.256592	0.009822
H	0.946172	4.775127	-3.521954
H	1.283030	3.094300	-2.935941
H	-0.356910	3.770433	-2.850604
O	5.663315	-3.834343	-2.105991
O	5.486619	-3.010910	0.010369
H	3.663058	-3.207531	-3.521947
H	2.039336	-2.657705	-2.935974
H	3.445606	-1.576511	-2.850736
O	-5.663129	3.833792	2.106372
O	-5.486354	3.011469	-0.010407
H	-3.662909	3.206991	3.522035
H	-3.445005	1.576118	2.850568
H	-2.039029	2.657641	2.936058
O	6.151430	2.987795	2.106529
O	5.350262	3.246518	-0.009939
H	4.609077	1.568150	3.521872
H	3.087207	2.193972	2.850742
H	3.322121	0.435761	2.935790
O	-0.488822	-6.820529	2.106946
O	0.135288	-6.256706	-0.009843
H	-0.946163	-4.774816	3.522051
H	0.356880	-3.770117	2.850641
H	-1.283077	-3.094037	2.935949

82

[**2**·Gd]+2H_2O E(gas)= -3081.84232811 A.U.

C	0.081975	2.812120	-0.606729
N	1.247937	3.384715	-0.363097
C	2.452434	2.678169	-0.788936
C	2.661256	1.376062	-0.113114
C	-1.233736	3.528079	-0.282242
C	-2.588561	-1.392927	-0.569067
N	-3.671135	-0.648365	-0.411301
C	-3.640855	0.713649	-0.932345
C	-2.643931	1.588683	-0.268477
N	-2.372303	2.789161	-0.798882
O	-1.526126	-0.914647	-1.047579
O	-2.046994	1.232583	0.789639
O	0.019320	1.644328	-1.085183
O	1.992214	1.046871	0.916537
C	-4.897299	-1.106754	0.278886
C	-2.978484	3.238405	-2.043922
C	1.405342	4.679890	0.336121
C	2.369200	-1.588087	-0.291382
N	2.240148	-2.845736	0.146418
C	1.113896	-3.577591	-0.429270
C	-0.230723	-3.102262	0.088030

C	3.660167	-0.816389	-0.067304
N	-1.343505	-3.513091	-0.524281
C	-2.591555	-2.867198	-0.147744
N	3.580678	0.531835	-0.596759
O	1.408530	-1.039150	-0.887948
O	-0.306956	-2.316752	1.065794
C	-1.335963	-4.324575	-1.734169
C	4.372013	0.846173	-1.781384
C	3.412177	-3.646417	0.580747
H	3.271108	3.482525	-0.605310
H	2.415534	2.530806	-1.872326
H	-1.246368	4.528559	-0.719109
H	-1.319447	3.642982	0.803080
H	-3.439731	0.680068	-2.007405
H	-4.748589	1.033818	-0.819568
H	1.273364	-4.642100	-0.238017
H	1.134715	-3.454074	-1.516974
H	4.436726	-1.401096	-0.590265
H	3.921347	-0.801849	0.997706
H	-2.733294	-2.936893	0.935023
H	-3.414213	-3.403879	-0.625072
H	5.287602	0.254679	-1.757395
H	3.823050	0.602500	-2.697631
H	4.644357	1.902511	-1.784508
C	4.022669	-4.153445	-0.791042
H	4.110156	-2.949937	1.049702
C	3.030898	-4.740223	1.555466
H	-2.243360	-4.930778	-1.759526
H	-1.301191	-3.691049	-2.627246
H	-0.484423	-5.004338	-1.740215
C	-6.237604	-0.602487	-0.378814
H	-4.935842	-2.194103	0.209847
C	-4.864641	-0.664230	1.738018
H	-4.011483	2.894447	-2.105078
H	-2.989160	4.329676	-2.059764
H	-2.418556	2.874377	-2.913188
C	2.558772	5.584302	-0.237735
H	0.488943	5.252521	0.191201
C	1.656684	4.437494	1.821157
H	3.936137	-5.084111	2.062611
H	2.318323	-4.413066	2.323095
H	2.637912	-5.602486	1.009854
O	4.745448	-3.299103	-1.348918
O	3.593464	-5.255842	-1.164056
H	-5.761591	-1.037621	2.238331
H	-3.980876	-1.041998	2.267438
H	-4.872785	0.427841	1.800330
O	-7.133126	-1.441911	-0.378732
O	-6.250208	0.610681	-0.744647
H	1.733339	5.404672	2.324264
H	0.856994	3.852846	2.291290

H	2.602767	3.907325	1.961901
O	2.282980	6.779827	-0.268172
O	3.642079	4.986794	-0.515911
Gd	-0.074983	0.011768	0.570318
O	-0.670437	0.640146	2.846763
O	1.360348	-0.903862	2.612947
H	-0.277332	0.101102	3.542234
H	-1.573473	0.889019	3.066152
H	2.159115	-0.391171	2.428679
H	1.517312	-1.816893	2.338067

97

[**4**·Gd]$^{3-}$+2H_2O E(gas)=-3763.04716224 A.U.

C	2.185508	-2.194612	0.024171
N	3.289763	-1.688747	-0.504725
C	3.788363	-0.452145	0.079434
C	2.917258	0.737911	-0.296602
C	1.548273	-3.444343	-0.559337
C	-2.923518	-0.744090	-0.053576
N	-3.025315	-1.921347	-0.648669
C	-2.203249	-3.003266	-0.127291
C	-0.733815	-2.811361	-0.465735
N	0.189034	-3.696395	-0.089463
O	-2.108961	-0.588967	0.919250
O	-0.379543	-1.777221	-1.102997
O	1.599805	-1.589199	0.978205
O	1.882589	0.538330	-1.002387
C	-4.165990	-2.222403	-1.524580
C	-0.056642	-4.911895	0.713251
C	4.119916	-2.458922	-1.443278
C	0.869356	2.930517	0.067811
N	-0.073853	3.634152	-0.545468
C	-1.432522	3.468812	-0.055263
C	-2.008369	2.110580	-0.426377
C	2.315250	3.025463	-0.391164
N	-3.239707	1.742846	-0.060178
C	-3.695695	0.455681	-0.576423
N	3.185280	1.971786	0.124803
O	0.560728	2.124286	0.999397
O	-1.292852	1.290964	-1.068308
C	-4.213506	2.587115	0.657575
C	4.335552	2.368024	0.963116
C	0.249914	4.739465	-1.461678
H	4.793543	-0.219541	-0.282785
H	3.828531	-0.549426	1.167464
H	2.200283	-4.278064	-0.285874
H	1.535485	-3.348388	-1.650239
H	-2.335862	-3.086270	0.955167
H	-2.483871	-3.964063	-0.571010
H	-2.098572	4.210804	-0.502275
H	-1.453329	3.601032	1.030407
H	2.672488	4.004452	-0.060125

H	2.336958	2.996430	-1.485598
H	-3.622917	0.463502	-1.669465
H	-4.737729	0.294987	-0.290078
C	5.765563	1.986928	0.396242
H	4.330651	3.460807	0.926397
C	4.154066	1.977238	2.425240
C	0.841026	5.991480	-0.677892
H	1.083112	4.385559	-2.081259
C	-0.880328	5.093967	-2.411370
C	-4.509781	4.008806	0.012012
H	-5.167885	2.067395	0.533644
C	-3.946029	2.660630	2.156206
C	-5.517353	-2.285952	-0.680722
H	-4.290257	-1.350557	-2.178698
C	-3.955422	-3.426458	-2.423873
C	-0.243569	-4.593476	2.192276
C	-1.144264	-5.918411	0.150003
H	0.872517	-5.482645	0.628009
C	4.842513	-3.677346	-0.715670
H	3.424713	-2.933513	-2.147572
C	5.063902	-1.602187	-2.267696
H	-0.490393	5.877712	-3.062517
H	-1.203846	4.234072	-3.007841
H	-1.758783	5.506372	-1.908636
O	0.790116	7.054473	-1.319108
O	1.341518	5.723916	0.436821
H	-4.853749	-3.502559	-3.038232
H	-3.071511	-3.316630	-3.061112
H	-3.863247	-4.366629	-1.874125
O	-6.447956	-2.859337	-1.268216
O	-5.476738	-1.688533	0.418740
H	5.575395	-2.288347	-2.944267
H	4.533690	-0.832902	-2.839167
H	5.834329	-1.107472	-1.670965
O	5.803704	-4.140341	-1.351802
O	4.303858	-4.049381	0.350429
H	-4.651473	3.390559	2.558254
H	-4.108024	1.690733	2.642504
H	-2.932163	3.009255	2.382094
O	-5.250657	4.704313	0.727771
O	-4.026460	4.215635	-1.120523
H	5.017349	2.372144	2.963448
H	3.226195	2.385031	2.838946
H	4.145231	0.892397	2.570673
O	6.678086	2.322449	1.170615
O	5.802130	1.454258	-0.734574
H	-0.406000	-5.545173	2.700959
H	0.634398	-4.086815	2.607868
H	-1.122139	-3.965503	2.375524
O	-1.349613	-6.867154	0.925531
O	-1.605249	-5.673367	-0.986801

Gd	0.069050	0.007472	0.331196
O	0.148503	-0.256610	2.731078
H	-0.723065	-0.303368	3.201403
H	0.612598	-1.094379	2.845586
O	-2.348102	-0.253962	3.614322
H	-2.598256	-0.527965	2.710890
H	-2.577691	0.681789	3.629544

97

[**6**·Gd]$^{3-}$+2H_2O E(gas)=-3763.05176604 A.U.

C	2.937413	-0.695458	0.039897
N	3.618153	0.298260	-0.503685
C	3.353243	1.651068	-0.028664
C	1.958518	2.129929	-0.406686
C	3.056335	-2.098384	-0.502096
C	-2.142678	-2.228557	0.145231
N	-1.632896	-3.323837	-0.399811
C	-0.318858	-3.758471	0.059230
C	0.783917	-2.798840	-0.353423
N	2.047070	-3.022523	0.006913
O	-1.516355	-1.645540	1.089671
O	0.497729	-1.764395	-1.025172
O	2.146192	-0.459028	1.021406
O	1.193412	1.360181	-1.056370
C	-2.266174	-4.040008	-1.529307
C	2.432674	-4.101488	0.945334
C	4.551218	0.125209	-1.637864
C	-0.908436	2.925616	0.112848
N	-2.145169	3.026882	-0.354773
C	-3.148852	2.114153	0.182235
C	-2.909501	0.677360	-0.252453
C	0.212914	3.747926	-0.488479
N	-3.721422	-0.300925	0.138220
C	-3.438606	-1.642332	-0.368428
N	1.540119	3.337088	-0.036223
O	-0.649853	2.107590	1.050882
O	-1.896432	0.404718	-0.966642
C	-4.805601	-0.098306	1.126616
C	2.302582	4.192738	0.899184
C	-2.524265	3.924147	-1.467254
H	4.169409	2.271336	-0.419659
H	3.468448	1.688027	1.057664
H	4.023526	-2.602241	-0.294932
H	2.967056	-2.049552	-1.590877
H	-0.325792	-3.848572	1.149120
H	-0.206021	-4.787381	-0.320281
H	-4.119450	2.537525	-0.117753
H	-3.129283	2.159095	1.275184
H	0.157950	4.829352	-0.285743
H	0.169812	3.640491	-1.575858
H	-3.384010	-1.595756	-1.459309
H	-4.346008	-2.229801	-0.132185

C	2.233338	5.753992	0.642567
C	1.902429	3.888332	2.339500
H	3.358666	3.950816	0.757647
C	-3.884136	4.708389	-1.268186
H	-1.764589	4.706678	-1.513828
C	-2.550719	3.163646	-2.789210
C	-6.131536	-0.929941	0.902805
C	-4.282331	-0.348485	2.539151
H	-5.120251	0.943718	1.037097
C	-2.220850	-5.619008	-1.443858
H	-3.330119	-3.797564	-1.496683
C	-1.676947	-3.573804	-2.856883
H	1.702071	-4.903023	0.817732
C	3.826912	-4.805032	0.696820
C	2.380504	-3.594592	2.384650
C	5.922319	0.902124	-1.481055
H	4.834473	-0.928867	-1.654386
C	3.874294	0.493371	-2.952715
H	-2.830121	3.878938	-3.564984
H	-1.586426	2.702326	-3.028591
H	-3.305237	2.369785	-2.769559
O	-4.063471	5.545311	-2.167910
O	-4.596546	4.402314	-0.285392
H	-2.184004	-4.129953	-3.647484
H	-1.797771	-2.496461	-3.014090
H	-0.606525	-3.799700	-2.910407
O	-2.901678	-6.142915	-2.339721
O	-1.526404	-6.138664	-0.540006
H	4.617420	0.359266	-3.740863
H	2.988649	-0.118152	-3.156227
H	3.562446	1.543514	-2.954677
O	6.695915	0.660853	-2.421364
O	6.055599	1.636537	-0.476779
O	2.932149	6.378114	1.458121
O	1.525454	6.160607	-0.304274
H	2.481620	4.551157	2.984471
H	0.836739	4.079456	2.498107
H	2.085882	2.839360	2.610847
O	-7.017806	-0.593377	1.704193
O	-6.141557	-1.802274	0.003703
H	-5.115061	-0.186278	3.225766
H	-3.939426	-1.383304	2.645212
H	-3.446645	0.311057	2.801538
O	3.977236	-5.783987	1.443982
O	4.598060	-4.317899	-0.162788
H	2.647199	-4.429254	3.035107
H	3.106017	-2.789529	2.541883
H	1.389773	-3.212239	2.660456
Gd	-0.062125	0.008493	0.383969
O	-0.164632	-0.155471	2.792083
O	2.315029	0.048216	3.692839

H	-0.592917	-1.000890	2.966472
H	0.691123	-0.111698	3.288673
H	2.560785	-0.191576	2.777918
H	2.534567	0.982822	3.768278
